# Physiological and Comparative Proteomic Analysis Reveals Different Drought Responses in Roots and Leaves of Drought-Tolerant Wild Wheat (*Triticum boeoticum*)

**DOI:** 10.1371/journal.pone.0121852

**Published:** 2015-04-10

**Authors:** Hui Liu, Muhammad Abdul Rab Faisal Sultan, Xiang li Liu, Jin Zhang, Fei Yu, Hui xian Zhao

**Affiliations:** 1 College of Life Sciences, Northwest A&F University, Yangling, Shaanxi 712100, P.R. China; 2 State Key Laboratory of Crop Stress Biology in Arid Areas, Northwest A&F University, Yangling, Shaanxi 712100, China; National Taiwan University, TAIWAN

## Abstract

To determine the proteomic-level responses of drought tolerant wild wheat (*Triticum boeoticum*), physiological and comparative proteomic analyses were conducted using the roots and the leaves of control and short term drought-stressed plants. Drought stress was imposed by transferring hydroponically grown seedlings at the 3-leaf stage into 1/2 Hoagland solution containing 20% PEG-6000 for 48 h. Root and leaf samples were separately collected at 0 (control), 24, and 48 h of drought treatment for analysis. Physiological analysis indicated that abscisic acid (ABA) level was greatly increased in the drought-treated plants, but the increase was greater and more rapid in the leaves than in the roots. The net photosynthetic rate of the wild wheat leaves was significantly decreased under short-term drought stress. The deleterious effects of drought on the studied traits mainly targeted photosynthesis. Comparative proteomic analysis identified 98 and 85 differently changed protein spots (DEPs) (corresponding to 87 and 80 unique proteins, respectively) in the leaves and the roots, respectively, with only 6 mutual unique proteins in the both organs. An impressive 86% of the DEPs were implicated in detoxification and defense, carbon metabolism, amino acid and nitrogen metabolism, proteins metabolism, chaperones, transcription and translation, photosynthesis, nucleotide metabolism, and signal transduction. Further analysis revealed some mutual and tissue-specific responses to short-term drought in the leaves and the roots. The differences of drought-response between the roots and the leaves mainly included that signal sensing and transduction-associated proteins were greatly up-regulated in the roots. Photosynthesis and carbon fixation ability were decreased in the leaves. Glycolysis was down-regulated but PPP pathway enhanced in the roots, resulting in occurrence of complex changes in energy metabolism and establishment of a new homeostasis. Protein metabolism was down-regulated in the roots, but enhanced in the leaves. These results will contribute to the existing knowledge on the complexity of root and leaf protein changes that occur in response to drought, and also provide a framework for further functional studies on the identified proteins.

## Introduction

Wheat (*Triticum aestivum* L.) is among the major staple crops in the world. Its yield is significantly affected by global climatic change and scarcity of water resources in the environment [1]. Drought is one of the environmental stresses that seriously limit crop production in the majority of agriculture fields in the world [2]. Recent global climate change has exacerbated this situation [3]. Therefore, studies on wheat drought response mechanisms are needed to develop wheat varieties that can tolerate water stress.

Plants are equipped with sophisticated and elaborate mechanisms to cope with environmental stresses, to which plants are constantly subjected to [4]. Of these environmental stresses, drought occurs most frequently. Complex drought responses are initiated by a massive transcriptional reprogramming upon the perception of water scarcity; these responses are preceded by diverse anatomical and physiological alterations, such as stomatal closure and synthesis of compatible osmolytes and antioxidants [4,5]. Some good agronomic traits such as drought tolerance are exhibited in common wheat progenitors, including *Aegilops tauschii* (2n = 2x = 14, DD), *T*. *urartu* (2n = 2x = 14, AA), *T*.*boeoticum* (2n = 2x = 14, A^b^A^b^) [6,7,8]. Wild wheat species have great potential as a source of genetic traits to improve the drought resistance of wheat cultivars because wild wheat species are highly tolerant to drought stress [9]. The wild wheat species, *T*. *boeoticum*, is tolerant to different kinds of environmental stresses, such as salt [10] and pathogenic infection [11]. *T*.*boeoticum* is more tolerant to drought than other wheat relatives, such as *T*.*dicoccoides*, *T*.*araraticum* and common wheat cultivars [8]. Results of the above mentioned studies indicate that *T*. *boeoticum* is a suitable and promising gene source for improving modern wheat.

Domestication of crop species and centuries of cultivation have improved production yields at the expense of crop germplasm diversity. Several genes associated with stress tolerance have been eradicated. Consequently, genebanks and landraces have recently gained prominence because of the identification of novel alleles for stress resistance [12]. Studies on molecular changes in wheat in response to abiotic stress have focused on differentially expressed genes and have identified several tolerance genes unique to wild emmer wheat species by comparative transcriptomic approaches [5]. However, identification of differentially expressed genes is generally insufficient if the aim is to determine the underlying molecular mechanism of drought stress because transcripts undergo translational and post-translational modifications to form functional proteins, i.e. the differential expressions of the two macromolecules are not always well correlated [13]. Proteomic approaches provide the missing information in DNA or mRNA analysis methods by focusing on the actively translated portion of the genome. Stress resistance is conferred by proteins that function in stress signaling, transcription regulation, cellular detoxification, protection of macromolecules, and other processes. Therefore, comparative proteomic approaches are powerful and promising tools for investigating plant stress responses. Several studies focused on proteomic changes in different plant organs, including roots and leaves, in response to salt stress in soybean (*Glycine max*), [14] and in wild tomato (*Solanum chilense*) [15]. Proteomic changes in leaves of wild and modern wheat under drought stress were studied to determine the differentially expressed proteins; the results revealed the differences in leaf tissue protein levels between modern and wild wheat genotypes in response to drought [16]. Until now, to the best of the authors’ knowledge, few studies focused on the proteomic changes in roots and leaves of tolerant wild wheat plants in response to drought stress. In two available proteomics studies on wheat grown under drought conditions, leaf tissue was used only [16, 17].

Our long term goal is to uncover the molecular mechanisms underlying drought tolerance in wild wheat (*T*. *boeoticum*), which was the most drought-tolerant species among the selected wild relatives of wheat in our laboratory [8]. The objective of this study is to elucidate proteomic responses to short-term drought stress in this species. Physiological responses were initially analyzed. Comparative proteomic analysis was then conducted on the roots and leaves of the control and the drought-treated wild wheat (*T*. *boeoticum*) plants. Drought stress (a moderate water deficit regime) was induced by using 20% PEG-6000, which can create water stress in plants hydroponically grown by modifying the osmotic potential of nutrient solution but no other side-effects [18], in hydroponically grown three-leaf stage seedlings. The proteomic analysis first identified, on large scale, the different drought responsive proteins in the roots and the leaves of *T*. *boeoticum* seedlings exposed to short-term drought stress, thereby elucidating the different responses of functional pathways to short-term drought condition between the two tissues.

## Material and Methods

### Ethics Statement

No specific permissions were required for the described field studies and for the location and activities. The location is not privately-owned or protected in any way. The field studies did not involve endangered or protected species.

### Plant materials and drought treatment

The wild wheat species *T*. *boeoticum* (2n = 14, A^b^A^b^) from Erebuni (Armenia) was used as an experimental material in this study.

Seeds of *T*. *boeoticum* were germinated under hydroponic conditions and were grown in a greenhouse with a day/night temperature regime of 20–22°C/15–18°C, 65–75% relative humidity and a light period of 16 h/day (regulated with supplementary light). A hydroponic nutrient solution (1/2 Hoagland solution) that contained all necessary nutrients for normal plant growth was supplied for wheat growth, and aerated using an air compressor. The Hoagland solution was developed by Hoagland & Snyder [19] Eight trays containing 80～100 plants were used for each experiment. At three-leaf stage, plants were subjected to drought stress by exposing the plants to 1/2 Hoagland solution containing 20% (g/ml) PEG 6000 (corresponding to -0.6 Mpa water stress) for 48 h.

The leaf and the root samples were randomly collected at 0, 24, and 48 h of drought treatment, rapidly frozen in liquid nitrogen, and then stored at -80°C for the extraction of proline, malondialdehyde (MDA), abscisic acid (ABA), soluble sugar, chlorophyll a/b, and protein. Fresh leaf and root samples were used to measure relative water content (RWC). Three biological replicates were included in each measurement, and this experiment was performed in triplicate independent repeat, in order to ascertain reproducibility.

### Estimation of RWC of leaves and roots

Wheat leaf and root RWCs were estimated according to the method of Smart and Bingham [20].

### Estimation of MDA in leaves and roots

The level of lipid peroxidation was measured in terms of MDA content. The MDA contents in wheat leaves and roots were determined according to the protocol of Hodges et al. [21].

### Determination of proline and soluble sugar content in leaves and roots

The proline content in wheat leaves and roots were measured according to the method of Bates et al. [22].

Total soluble sugars in wheat leaves and roots were determined according to the protocol reported by Farhad et al. [23].

### Measurement of ABA level in wheat leaves and roots

ABA in wheat leaves and roots was extracted according to the methods of Nehemia et al. [24], and ABA contents in the root and leaf tissues were measured by using the ABA ELISA quantification kit (Agrisera, Sweden) according to the included instructions.

### Leaf pigment estimation

The contents of leaf chlorophyll a and b were estimated based on the protocol reported by Farhad et al. [23].

### Measurement of photosynthesis

Net photosynthetic rate (Pn), stomatal conductance, intercellular CO_2_ concentration, and transpiration speed of the second wheat leaves were measured at 0, 24, and 48 h of drought treatment by using a portable gas analysis system (LI-COR 6400), with a light-emitting diode light source (LI-COR Inc., Lincoln, NE). At least five replicates for each treatment were measured.

### Protein extraction

Proteins of the leaf and the root samples were prepared according to the previously reported protocol [25], with slight modifications. Frozen leaves and roots (0.5 g) were ground to a fine powder with pre-chilled mortar and pestle in liquid nitrogen. The powder was mixed in a cuvette with 10 ml cold acetone, containing 10% (g/ml) TCA and 0.07% (v/v) β-mercaptoethanol, to precipitate proteins overnight at -20°C. Precipitated proteins were centrifuged at 19,000 *g* for 1 h at 4°C and were washed thrice with ice-cold acetone containing 0.07% (v/v) β-mercaptoethanol. To eliminate acetone, the collected protein pellets were dried with liquid nitrogen (N_2_).

The dried powder was thoroughly re-suspended in 1 ml of lysis buffer [7 M urea, 2 M thiourea, 4% (g/ml) CHAPS, 65 mM DTT, and 0.01% (v/v) protein inhibitor]. The suspension was incubated at 4°C for 1.5 h and then centrifuged at 19,000 g for 1h at 4°C to remove any insoluble material. The supernatant containing soluble proteins was quantified by a 2-D Quant Kit (GE Healthcare Life Sciences) using bovine serum albumin (BSA; 1mg/ml) as a standard. The quantified protein samples were stored in aliquots at—80°C until analysis by two-dimensional gel electrophoresis (2-DE) and Western blot.

### Two-dimensional electrophoresis (2-DE), gel staining, and image analysis

2-DE was performed according to the modified method of O'Farrell [26]. The immobilized pH gradient (IPG) strips of pH 4 to 7 (17 cm, linear, Bio-Rad Laboratories), which was confirmed to be optimal for separation of the prepared proteins by our lab (data not shown), were used for isoelectric focusing (IEF), and the second dimension SDS-PAGE was used with 12% and 11.25% gels for the leaf and root tissues, respectively.

For the first dimension electrophoresis, 350 μl IEF rehydration buffer [7 M urea, 2 M thiourea, 4% (g/ml) CHAPS, 65 mM DTT, 0.2% (g/ml) bio-lyte, and 0.01% (g/ml) bromophenol blue] containing 600 μg (leaf) or 250 μg (root) protein was loaded onto the IPG strips and actively rehydrated for 14 h at 50 V and 20°C. IEF was performed on the PROTAIN IEF Cell system (Bio-Rad, USA) by applying a voltage of 200 V for 1 h, 500 V for 1 h, 1000 V for 1 h, and 10,000 V for longer than 5 h. A voltage of 10,000 V was maintained until a total of 80 kVh was reached. After IEF, the strips were equilibrated in 10 ml of reducing equilibration buffer [6 M urea, 1.5 M Tris-HCl (pH 8.8), 20% (v/v) glycerol, 2% (g/ml) SDS, a trace of bromophenol blue, and 2% (g/ml) dithiothreitol (DTT)] for 15 min, and then in an equilibration buffer containing 2.5% (g/ml) iodoacetamide for another 15 min. The second dimension SDS-PAGE was performed using the PROTEAN II xi Cell system (Bio-Rad，USA) at 14°C and 5 mA/gel for 45 min and then in 20 mA/gel until the bromophenol blue reached the end of the gel. Protein ladders were also loaded onto the gels.

Protein spots in 2-DE gels for leaf samples were detected by staining with Coomassie Brilliant Blue (CBB) G-250 [27], and de-staining with double-distilled H_2_O. Protein spots in 2-DE gels for root samples were detected by silver staining according to the method of Mireille Chevallet [28]. Three biological repeats were performed for each sample. Each gel was scanned by UMAX PowerLook 2100XL image scanner (UMAX Systems GmbH, Willich, Germany) at 300 dpi (dot per inches) resolution. Image analysis was performed by using PDQuest 8.0.1 software (Bio-Rad Laboratories, Hercules, CA). After automated detection, matching, and normalization, further editing was preformed manually to prevent the occurrence of discrepancy during spot selection. Three independent biological replicates were used to create “replicate groups”. Statistical, quantitative, and qualitative “analysis sets” were developed between the control (0 h of drought-treatment) and the stress-treated (24 or 48 h of drought treatment) samples. In the statistical sets, the Student’s t-test and a significance level of 95% were selected. In the quantitative sets, the upper and lower limits were set to 1.5 and 0.66, respectively. Then, Boolean analysis sets were created between the statistical and quantitative or qualitative sets. The protein spots from the Boolean sets were compared amongst three biological replicates. Only spots displaying reproducible change patterns were considered to be differentially changed proteins for further mass spectrum identification.

### In-gel digestion and matrix-assisted laser desorption/ionization-tandem time-of-flight (MALDI-TOF/TOF) analysis

The differentially changed protein spots (DEPs) were manually excised from the gels. In-gel digestion of DEPs was performed according to a previously described protocol [29]. The excised gel spots were destained twice with 100 mM NH_4_HCO_3_ in 30% acetonitrile (ACN). The protein spots were digested overnight (16 h) with 20 μl of 50mM NH_4_HCO_3_ containing 0.01 mg/ml sequencing-grade modified trypsin (Promega, Madison, WI, USA) at 37°C. The peptides were extracted thrice with 0.1% trifluoroacetic acid (TFA) in 60% ACN. The extracts were pooled together and lyophilized. The lypholized tryptic peptides were dissolved in 5 mg/ml cyano-4- hydroxycinnamic acid consisting of 50% ACN and 0.1%TFA.

Tryptic peptide analysis was performed on 4800 Plus MALDI TOF/TOF Analyzer (Applied Biosystems, USA). Peptide mass maps were generated in positive ion reflector mode (2 kV accelerating voltage) with 355 nm laser shots per spectrum. The signal to noise ratio of 50 was the minimal criterion to define mass peaks, and a peptide mass fingerprint (PMF) scan area of 800 Da to 4000 Da was selected. Up to 10 of the most intense ions were selected as precursors for MS/MS acquisition. MS/MS-positive ion mode was operated with 2 kV collision energy. Using the individual PMF spectra, peptides that exceeded a signal-to-noise ratio of 20 and passed through a mass exclusion filter were subjected to fragmentation analysis. The parameters for peak matching were as follows: Min S/N: 20; mass tolerance: 0.2 Da; min peaks to match reference masses, 4; and max outlier error, 100 ppm. Total shots for each MS spectrum were 2000, whereas 3000 shots were used for MS/MS. The data were calibrated using the ABI 4700 Calibration Mixture (Applied Biosystems, Foster City, CA). MS and the MS/MS spectra were searched against the NCBI database (Viridiplantae, 20140911) using the software GPS Explorer (Applied Biosystems) and MASCOT version 2.1 (Matrix Science, London, UK) with the following parameters: National Center for Biotechnology Information (NCBI) non-redundant protein database (released data Sep.11, 2014; including 45166402 proteins), and species restriction to Viridiplantae (green plants). The other parameters were as follows: trypsin cleavage; one missed cleavages allowed; peptide mass tolerance set to ±100 ppm; and fragment tolerance set to ± 0.4 Da. Credible results for the MALDI-TOF/TOF MS were the hits with high protein scores, similar molecular mass (*Mr*) and isoelectric point (pI) as experimental *Mr* and pI, and protein score confidence interval (*C*. *I*.*%*) of above 95%.

### Western blot analysis

Western blot analysis using the horseradish peroxidase (HRP)-conjugated rabbit-anti-X (X indicates the polyclonal antibodies of each selected protein) was performed according to the protocol previously reported [30]. About 16 μl (25 μg) protein sample was boiled in 4 μl of sample buffer [250 mM Tris-HCl (pH 6.8), 10% (g/ml) SDS, 0.5% (v/v) bromophenol blue, 50% (v/v) Glycerol and 5% (v/v) β-mercaptoethanol] and then separated by 12% SDS-PAGE. After the separated proteins were electro-blotted onto polyvinylidene (PVDF) membrane (Millipore) using Trans Blot system (Bio-Rad), the PVDF membrane was blocked for 1 h at 25°C with PBST solution [100 mM Tris HCl (pH 7.5), 0.9% (g/ml) NaCl, 0.1% (v/v) Tween-20] containing 4% (g/ml) skimmed milk powder. The membrane was washed thrice with PBST solution and then incubated overnight with the HRP-conjugated rabbit-polyclonal antibody (Beijing Protein Innovation Co.,Ltd., China, 1:1000) at 4°C. After washing thrice with PBST solution, the membranes were incubated with the secondary antibodies of HRP-conjugated Goat-anti-rabbit-IgG (Beijing Protein Innovation Co.,Ltd., China, 1:5000) for 1 h at room temperature. After washing thrice (10 min each time) with PBST solution, detection was performed using EasyBlot ECL kit (Thermo Scientific, USA). The membranes were scanned for the signal intensity of each band by using Gel Doc XR system (Bio-Rad, USA).

### Statistical analysis

All statistical analyses were performed by using SPSS 19.0 statistical software (SPSS Science, Chicago, IL, USA). Each data was presented as a mean ± standard deviation (SD). Comparisons were made by one-way analysis of variance (ANOVA). Significance was set at *P* < 0.05.

## Results and Discussion

### Physiological changes in roots and leaves of wild wheat plants under short-term drought treatment

**Fig 1:**
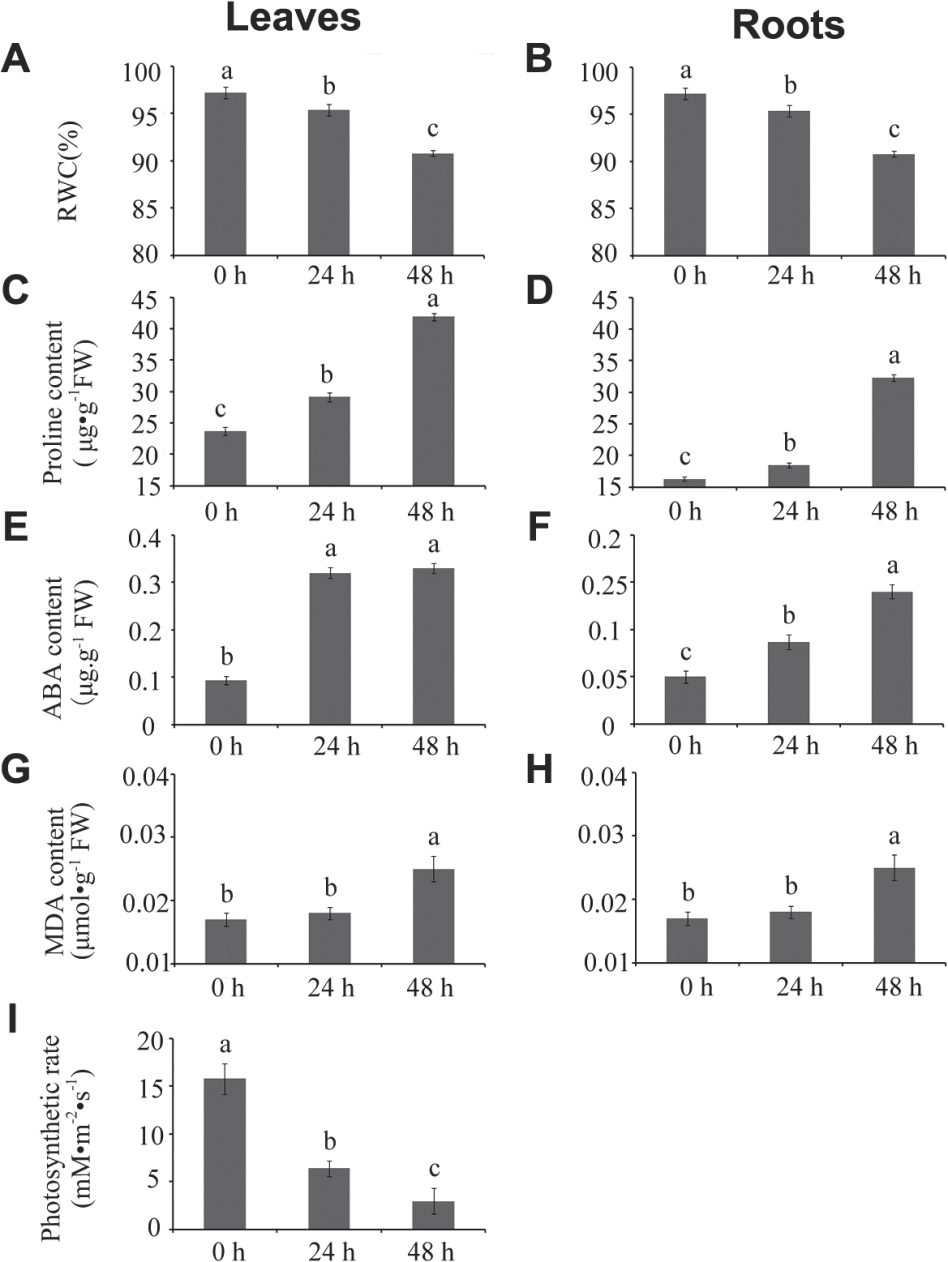
Relative water content, proline, ABA, MDA and chlorophyll a contents, and photosynthesis rate. Time course of the changes in RWC and proline, ABA and MDA contents in leaves (A, C, E, and G) and in roots (B, D, F, and H) and chlorophyll a (I) and photosynthesis rate (J) in leaves of *T*. *boeoticum*. Plants were grown in 1/2 Hoagland solution until the three-leaf stage and then were transferred to 1/2 Hoagland solution containing 20% PEG6000 (-0.6 Mpa) for 48 h to induce drought stress. Root and leaf samples were collected for analysis after 0, 24, and 48 h of drought stress. The data are the mean value±standard deviation (SD) of three independent biological repeats. The letters above the histogram indicate the statistical significance at the level of 0.05 (*p*≤0.05).

To investigate the responses of wild wheat (*T*. *boeoticum*) to short-term drought stress, plants were exposed to 1/2 Hoagland solution containing 20% PEG (6000) for 48 h during the three-leaf stage. The leaf apices of the drought-treated plants were slightly withered and were yellow in color (data not showed), thereby indicating that physiological changes occurred in the plants under drought stress. To determine the effects of short-term drought stress on the wild wheat plants, RWC and the contents of free proline, soluble sugar, MDA, and ABA in leaves and roots were detected after 0, 24, and 48 h of drought-treatment. Leaf chlorophyll content and photosynthesis rate were also determined. Results showed that the RWC in the leaves was reduced by 1.92% after 24 h of drought-treatment and by 6.64% after 48 h compared with that of 0 h (control). In roots, RWC was reduced by 9.47% after 24 h and by 13.66% after 48 h (Fig 1 and S1 Table), thereby indicating that the drought treatment caused mild to medium drought stress in the wheat plants [31]. Dattaet al. [30] also found that RWC decreased in short-term drought-stressed wheat plants under laboratory conditions. However, under long-term stress, the RWC initially declined and then remained relatively constant after 28 d, thereby indicating that long-term drought stress induces structural and functional reorganization, thus, wheat plants showed good adaptation to long-term drought conditions [32]. Our results also indicated that the contents of free proline and soluble sugar were significantly increased in both the leaves and the roots of the wild wheat plants under drought treatment, such increase being greater with drought duration. Free proline content was increased by 23.0%～77.0% in the leaves and 13.35%～97.6% in the roots from 24 h to 48 h of drought treatment. Soluble sugar content was increased by 7.1%～46.7% in the leaves and by 121.2%～189.9% in the roots from 24 h to 48 h of drought treatment (Fig 1 and S1 Table). These substances are important and efficient osmolytes, a high content of these substances may cause a low water potential of cells [33], considering the osmotic regulation that occurs in plants under water stress [34]. In addition, ABA content was increased greatly in the roots and the leaves of the wild wheat plants under drought treatment, with an increase of 74%～180% in roots and 244%～255% in leaves after 24 h to 48 h of drought stress (Fig 1 and S1 Table). The increase of ABA content in the leaves was much more rapid and much greater than that in the roots. Similar results were also found by Rubin Nan in wheat [35]. These results could be explained by the rapidly invoked hydraulic message in root, such message was instantly transduced to the leaf, thereby inducing ABA accumulation instantaneously in the leaf [35].

As reported, plants under abiotic stress conditions can yield reactive oxygen species (ROS), thereby leading to membrane lipid peroxidation, which can disturb the metabolic system within organisms, hence, the level of MDA, a product of membrane lipid peroxidation, would be increased [36]. The level of MDA in plants indicates the degree of injury to the plasma membrane system. To evaluate the effects of short-term drought treatment on the plasma membrane system in wild wheat plants, MDA contents in the roots and the leaves were detected separately. The result indicated that MDA levels were not significantly changed in the both tissues (organs) after 24 h of drought treatment. However, MDA level was increased by 47.06% and 23.33% in the leaves and the roots, respectively, after 48 h of drought treatment(Fig 1 and S1 Table), thereby suggesting that plasma membrane damage was present to a certain extent at this time point.

Furthermore, analysis also indicated that chlorophyll *a* and *b* contents in the wild wheat leaves were significantly reduced after 24 h of drought treatment, and the depletion of the chlorophyll *a* and *b* was exacerbated after 48 h of drought treatment (Fig 1 and S1 Table). The Pn of the wild wheat leaves also decreased significantly from 15.77 mmol•m^-2^•s^-1^ (control plants) to 6.39 (plants under 24 h of drought treatment) and 2.97 (plants under 48 h of drought treatment). Transpiration rate (Tr), stomatal conductance (Gs), and intercellular CO_2_ concentration also decreased significantly in drought-stressed plants (Fig 1 and S1 Table). Our results were consistent with those of previous report in wheat [37].

Taken together, RWC was reduced by 9.47%～13.66% in the roots and 1.92%～6.64% in the leaves of the wild wheat seedlings exposed to 20% PEG-6000 for 24～48 h, thereby indicating the occurrence of mild to medium drought stress in the plants. The ABA level was greatly increased in the both organs, but the increase was much more rapid and greater in the leaves than in the roots. The osmolytes free proline and soluble sugars were significantly increased in the both organs. The deleterious effect of drought on the seedlings mainly targeted photosynthesis.

### Proteomic changes in the roots and the leaves of wild wheat plants under short-term drought-treatment

To understand the proteomic responses of wheat seedlings to short-term drought stress, the changes in leaf and root proteomes of the wild wheat plants at 0, 24, and 48 h of drought treatment were analyzed by 2-DE. Three independent biological replicates were performed in this 2-DE experiment. Representative gel images of wild wheat leaf and root proteomes at the three time points are shown in S1 Fig For leaf proteome, 648±21, 793±18, and 732±23 protein spots were detected at 0, 24, and 48h of drought treatment, respectively (S1A, S1B and S1C Fig). By spot-to-spot comparisons and statistical analysis, we identified a total of 115 protein spots that exhibited at least 1.5-fold change (*p≤*0.01) in abundance in at least one time point (S1 Fig and Table 1). Among the 115 DEPs, 56 were up-regulated after 24 and 48h of drought treatment, and 23 were down-regulated (Table 1). However, one spot (spot L85) was up-regulated at 24 h, but down-regulated at 48 h of drought-treatment. Three spots (spot L44, L51 and L64) showed the opposite trend (S2 Table). Although the majority of spots showed quantitative changes, some spots showed qualitative changes. For example, 17 spots were newly induced under drought treatment, whereas 3 spots were completely suppressed (Table 1).

**Table 1:** Number of differentially changed protein spots (DEPs) in the leaves and the roots of *T*. *boeoticum* plants at different time points of drought-treatment.

	Number of DEPs (compared with 0 h of drought-treatment)					
DEPs with different fold changes	leaf			root		
	24 h	48 h	Overlapped	24 h	48 h	Overlapped
**Up-regulated**						
1.5–2 times	26	22	18	6	6	6
2–5 times	26	18	16	20	18	17
≥5 times	5	5	5	7	7	7
Induced	17	17	17	16	16	16
**Total**	**74**	**62**	**56**	**49**	**47**	**46**
**Down-regulated**						
1.5–2 times	20	11	11	7	8	7
2–5 times	8	7	7	24	27	24
≥5 times	2	2	2	8	8	8
Suppressed	4	4	3	6	7	4
**Total**	**34**	**24**	**23**	**45**	**50**	**43**

For the root proteome, 862±17, 889±20, and 1066±22 protein spots were detected at 0, 24, and 48 h of drought treatment, respectively (S1D,S1E and S1F Fig), and a total of 102 spots exhibited at least 1.5-fold change (*p≤*0.01) in abundance in at least one time point (Table 1). Among the 102 protein spots, 46 were up-regulated at 24 and 48 h of drought treatment, whereas 43 were down-regulated (Table 1). Three protein spots (spot R68, R73, and R75) were up-regulated at 24 h, but down-regulated at 48 h of drought treatment (S3 Table). Among the 102 DEPs, 16 were newly induced and 4 were completely suppressed under drought treatment (Table 1).

All the above mentioned DEPs were analyzed by MALDI-TOF-TOF. A total of 98 out of the 115 DEPs in the leaves and 85 out of the 102 DEPs in the roots were successfully identified (S4 and S5 Tables). Among the 98 DEPs identified in the leaves and the 85 in the roots, 94 (96%) and 77 (91%) were documented in the current database as putative functional proteins, respectively (S4 and S5 Tables). However, four identities in leaves (spots L3, L11, L33, and L88) and 8 in roots (spots R39, R45, R51, R56, R58, R68, R70, R73) were annotated either as unknown or hypothetical proteins. To acquire functional information for these identities, their sequences were used as queries to search for homologs using BLASTP (NCBI). The corresponding homologs with the highest similarity are shown in Table 2. All hits shared at least 47% sequence similarity, thereby suggesting that the functions of these proteins may be similar to those of their homologs.

**Table 2:** Corresponding homologs of the unknown proteins.

**Spot ID**	**NCBI accession no** ^a^	**Homologs**				
		**NCBI accession no** ^b^	**Protein name**	**Organism**	**Identities** ^c^	**Positives** ^d^
**Leaf unknown proteins**						
					**%**	**%**
L3	gi|307110690	EIE24754.1	aminopeptidase N, partial	*Coccomyxa subellipsoidea* C-169	63	75
L11	gi|125580806	XP_003516570.1	uncharacterized LOC100777163	*Glycine max*	38	47
L33	gi|238014852	gb|ABF95120.1|	anthranilate synthase component I family protein	*Oryza sativa* Japonica Group	89	97
L88	gi|326523645	XP_003568528.1	PREDICTED: thylakoid lumenal 15 kDa protein 1, chloroplastic-like	*Brachypodium distachyon*	89	90
**Root unknown proteins**						
R39	gi|145349320	EHR76240.1	thioesterase superfamily protein	uncultured marine group II euryarchaeote	30	47
R45	gi|242049774	NP_001149850.1|	ribulose-phosphate 3-epimerase	*Zea mays*	99	99
R51	gi|302762607	gb|ADE88152.1|	caffeyl alcohol/5-hydroxyconiferyl alcohol 3/5-O-methyltransferase-like 1	*Selaginella moellendorffii*	99	99
R56	gi|242066452	gb|ACG45465.1|	phospholipid hydroperoxide glutathione peroxidase	*Z*. *mays*	92	93
R58	gi|125547139	dbj|BAJ99344.1|	predicted protein (chromosome segregation protein SMC)	*Hordeum vulgare* subsp. *vulgare*	37	57
R68	gi|242091237	gb|AFW78925.1|	putative TCP-1/cpn60 chaperonin family protein	*Zea mays*	97	98
R70	gi|255081574	gi|78708135	retrotransposon protein, putative, Ty3-gypsy subclass	*O*.*sativa* Japonica Group	50	67
R73	gi|51969886	XP_002870124.1|	transcription factor	*Arabidopsis lyrata* subsp. *lyrata*	95	97

BLASTP (NCBI) was used to search the homologs of the unknown proteins in S4 and S5 Tables. The homologs with the highest homology are shown.

a: protein accession number of the unknown proteins listed in S4 and S5 Tables

b: The accession number of the homologs

c: The extent to which two amino acid sequences are invariant

d: The similarities based on the scoring matrix used.

Generally, the apparent *M*r (molecular weight) value anticipated by SDS-PAGE has an error deviation of approximately ±10% compared with the theoretical value. However, some identified proteins appeared to be the partially degraded products of their intact proteins because their observed *M*r values were much smaller than the theoretical ones. An example is the Rubisco large subunit (RLS), which is the most abundant protein in leaves. The RLS was identified from multiple spots (S2 Fig). Five spots (L4, L5, L10, L52, and L69) were identified as the same protein: RLS, four (L4, L5, L10 and L69) of them had the observed *M*r of 51.8, 51.6, 49.7, and 29.43 kDa, respectively, which were smaller than the theoretical *M*r value (52.4 kDa), and the remaining one (spot L52) had the observed *M*r of 53.79 kDa, similar to the theoretical value (S4 Table). This implied the existence of the Rubisco large subunit fragmentation under drought stress. Moreover, two spots (spot L67 and L84) were identified as putative carbonic anhydrase, with the observed *M*r of 37.29 and 31.70 kDa, respectively. These values were greater than theoretical *M*r value (28.51 kDa) of the carbonic anhydrase, thereby indicating that these proteins may be products of post-translational modification (S2 Fig, S4 Table). These results suggested the existence of post-translational modification or degradation of proteins in responses to drought stress in wild wheat seedlings. Similar results were observed in *Synechocystis* under UV-B stress [38].

Furthermore, a general comparison was conducted between the leaf and the root DEPs. Results showed that only 18 identities representing 6 unipros were mutual (S6 Table), thereby indicating the tissue-specific responses to drought stress at protein level in wild wheat leaves and roots.

A total of 98 and 85 differentially changed protein spots representing 87 and 80 unipros in leaves and roots, respectively, were identified, and only 18 identities representing 6 unipros were common.

### Validation of differentially expressed proteins by Western blot analysis

**Fig 2:**
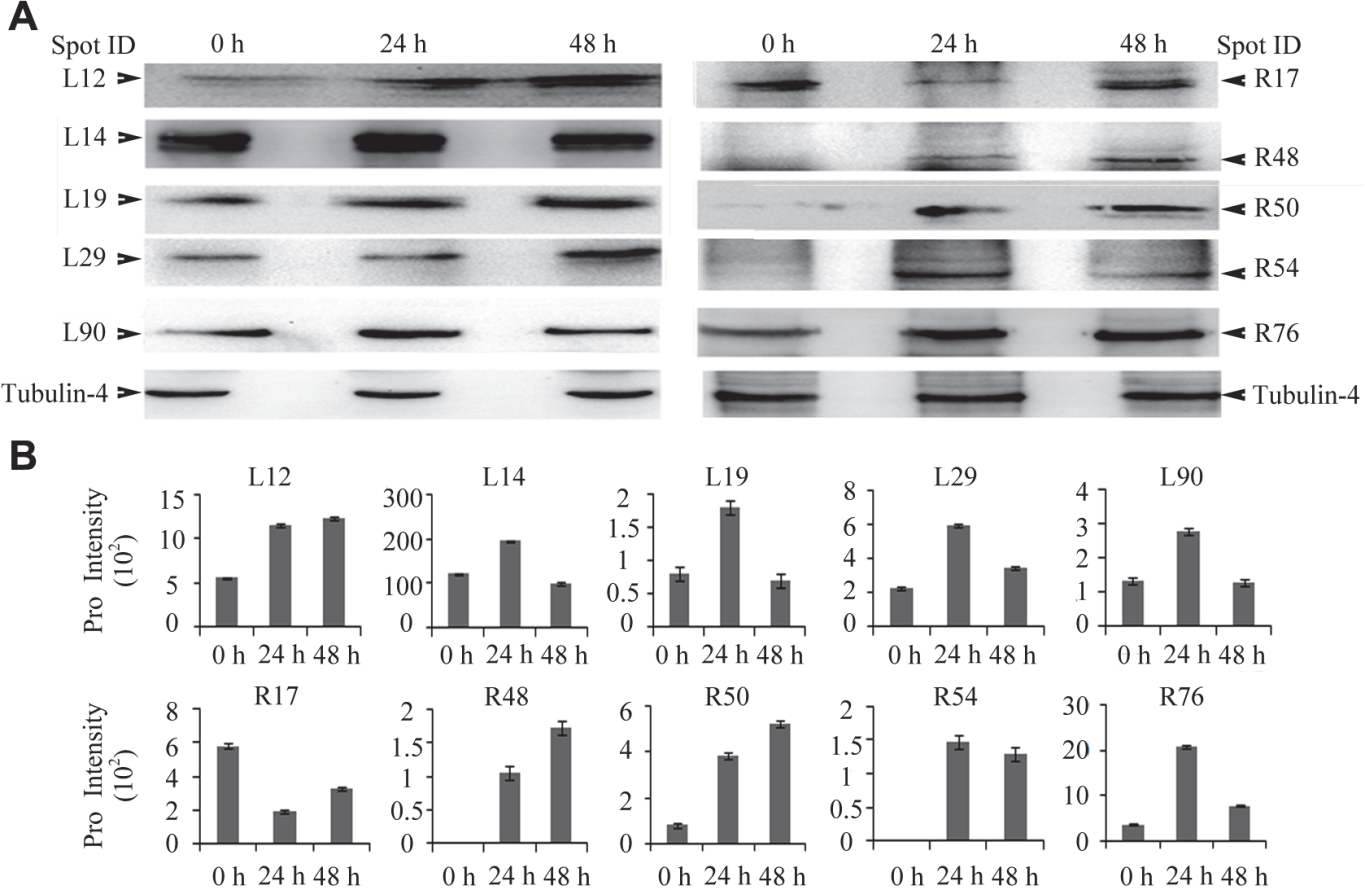
Comparison of the abundance of differentially changed proteins determined by Western blot analysis (A) and 2-dimensional electrophoresis analysis (B). 0 h, 24 h, and 48 h represent the time of drought treatment. Spot ID indicates spot identities; Spot ID with ‘L’ and ‘R’ represent the spot from leaf and root samples, respectively. The spot identities correspond to those in S2, S3, S4, and S5 Tables.

To confirm the changes of the DEPs identified in 2-DE analysis, the five DEPs in leaves, i.e., plastid glutamine synthetase 2 (spot L12), phosphoribulokinase (spot L14), fructose-1,6-bisphosphate aldolase (spot L19), carbonic anhydrase (spot L29), and heat shock protein 70 (spot L90), and the five DEPs in roots, i.e., plastid glutamine synthetase 2 (spot R17), fructose-bisphosphate aldolase (spot R48), 14-3-3-like protein GF14-B-like (spot R50), glutathione S-transferase-N (spot R54), and HSP70 (spot R76), were randomly selected for Western blot analysis. The protein samples collected at the three time points (0, 24 and 48 h) of drought stress were subjected to Western blot. Tubulin-4 was used as an internal protein reference in leaves and roots (Fig 2A). Results showed that the relative abundance of all these DEPs, as determined by Western blot analysis, followed similar trends as those tested by proteomic analysis (Fig 2B), thereby suggesting that the proteomic analysis results were reliable.

### Functional classification and subcellular localization of the DEPs in wild wheat leaves and roots

**Fig 3:**
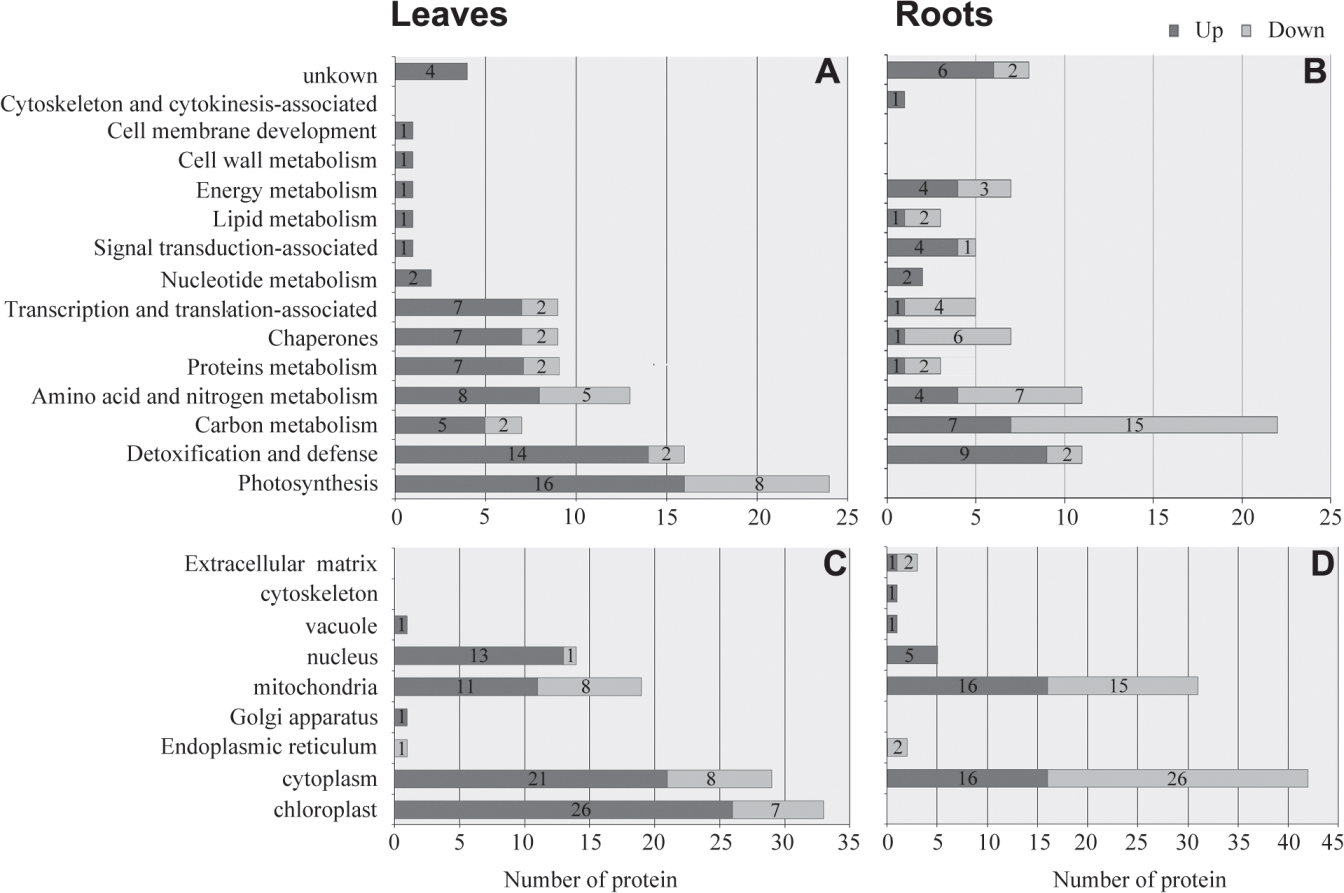
Functional classification (A and B) and subcellular localization (C and D) of the differentially changed proteins (DEPs) in leaves and roots of *T*. *boeoticum* plants treated with 20% PEG6000. DEPs were classified into 14 and 12 functional groups in the leaves (A) and the roots (B), respectively. The DEPs were also classified according to the subcellular localization predicted by WOLFPSORT (http://wolfpsort.org) and ESLpred (http://www.imtech.res.in/raghava/eslpred/) in the leaves (C) and the roots (D), respectively. Black and white bars represent the up- and down-regulated proteins, respectively.

Based on the metabolic and functional features, all the identified DEPs in leaves and roots were classified into 15 categories: detoxification and defense, carbon metabolism, amino acid and nitrogen metabolism, proteins metabolism, chaperones, transcription and translation-associated proteins, photosynthesis, nucleotide metabolism, signal transduction-associated proteins, lipid metabolism, energy metabolism, cell wall metabolism, cell membrane development, cytoskeleton, and cytokinesis-associated proteins (Fig 3A and 3B, S3 Fig). An impressive 86% of these identified proteins were implicated in the first eight functional groups, and the top four largest functional groups were proteins involved in carbon metabolism (15.8%), detoxification and defense (14.8%), amino acid and nitrogen metabolism (13.1%), and photosynthesis (13.1%) (S3 Fig). The numbers of DEPs belonging to the categories of transcription and translation-associated proteins, protein metabolism-related proteins, carbon metabolism-related proteins, energy metabolism, signal transduction-associated proteins, and unknown proteins were largely different between the leaves and the roots (Fig 3A and 3B). This finding implied the different responses of these metabolic pathways to short-term drought stress in the two tissues (organs). This would be analyzed further in the following Sections.

The subcellular localization of all the identified DEPs was predicted by WoLFPSORT prediction (http://wolfpsort.org) and ESLpred (http://www.imtech.res.in/raghava/eslpred/). Results revealed that the majority of the identified DEPs in the leaves were localized in the chloroplast, mitochondria, cytoplasm, and nucleus (Fig 3C), whereas most of the identified DEPs in the roots were localized in the cytoplasm and mitochondria (Fig 3D). The number of the DEPs localized in the nucleus of leaf cells was much higher than that of root cells, whereas the opposite trend was observed in the cytoplasm.

Photosynthesis was suppressed, but the number of up-regulated proteins localized in leaf chloroplasts was unexpectedly much larger than that of the down-regulated proteins in wild wheat plants under drought stress (Fig 3C). Similar results were also observed in drought-stressed soybean [39].

### Signal transduction-associated protein levels were highly enhanced in wild wheat roots in response to short-term drought stress

**Fig 4:**
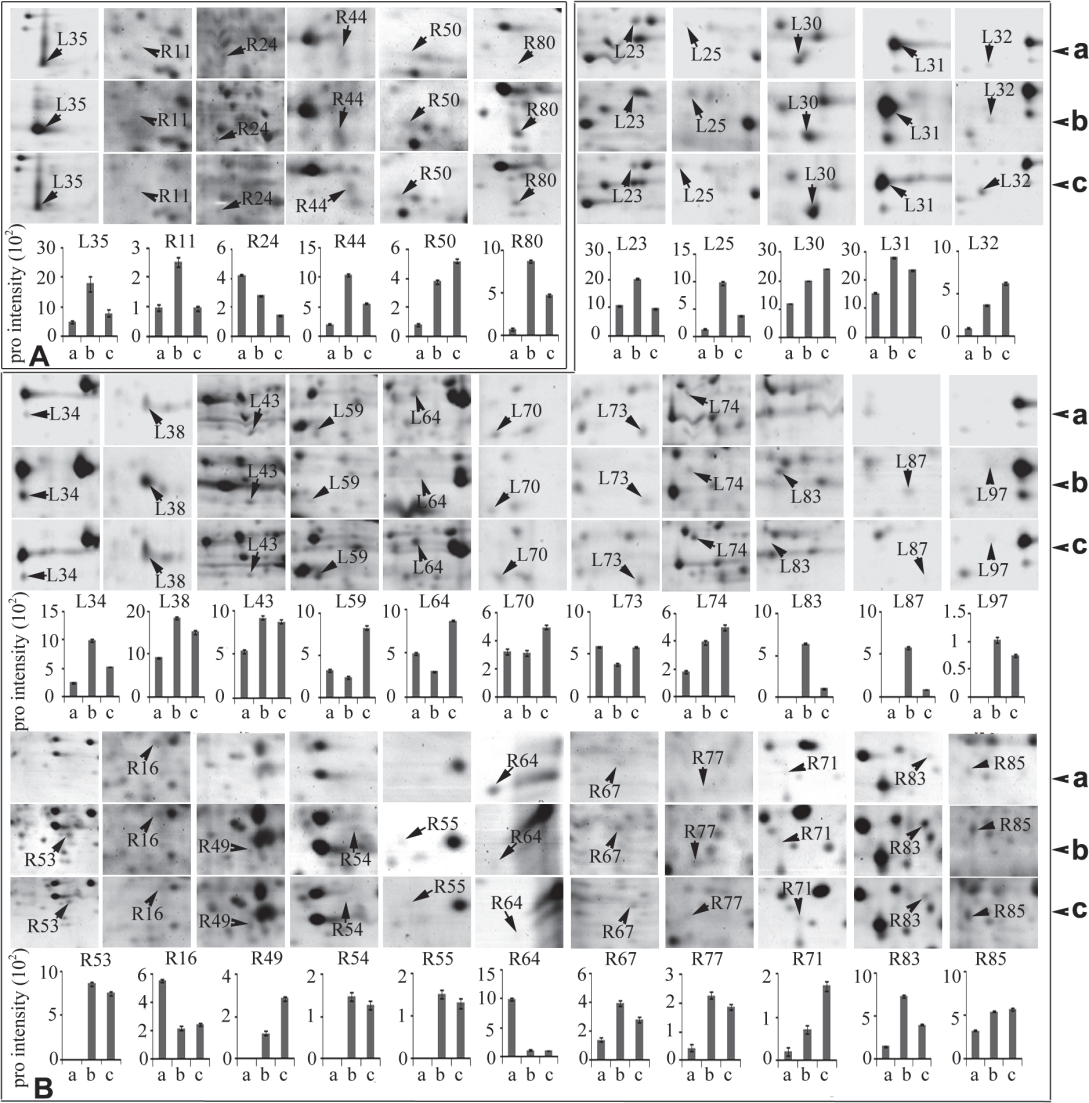
Differentially changed proteins associated with signal transduction (A) and antioxidation and defense (B) in leaves and roots of *T*. *boeoticum* plants exposed to 20% PEG6000 for 48 h to induce drought stress. All protein spots are enlarged from S1A–S1F Fig and protein intensity values are from S2 and S3 Tables. a, b, c represent 0, 24, and 48 h of drought treatment, respectively. Protein spot identities correspond to those in S1 Fig and S2, S3, S4, and S5 Tables. Error bars represent standard deviation (n = 3).

Plant stress response is a dynamic process that is dependent on stress intensity and duration and can be distinguished into several stages [40]: an initial alarm phase when stress causes a shock to a non-acclimated plant, and the level of plant stress tolerance decreases; an acclimation phase that lasts for several days and which leads to the establishment of a new homeostasis in plant metabolism under stress (the level of plant stress tolerance increases during acclimation phase); a maintenance phase, during which a newly established homeostasis is maintained under stress conditions (the level of plant stress tolerance remains stable during maintenance phase); and an exhaustion phase, which occurs if the stress duration is too long and if a plant fails to maintain stress-induced homeostasis (the level of plant stress tolerance decreases during the exhaustion phase). Each stage of plant stress response corresponds to a different proteome composition [41]. As the primary organ that perceives water deficit under drought stress, the root responds rapidly by adjusting its cellular processes and growth and metabolic pathways. Thus, the roots play an important role in the drought response and adaption of the plant. ABA, a plant hormone that inhibits growth, acts as an intercellular signal (a primary messenger) that is perceived by its receptor in the plasma membrane to trigger plant cellular responses to environmental stress. Physiological analysis results in the present study indicated that the ABA level was significantly increased in the roots and leaves of the wild wheat plants exposed to drought stress (Fig 1 and S1 Table). Comparative proteomic data also showed that the levels of several proteins involved in signal transduction were significantly enhanced in the roots of the wild wheat plants at 24 h of drought stress (Fig 4A). These proteins included pyrabactin resistance 1 (PYR1) (spot R44), two Ca^2+^ receptors, CML49-like isoform 1 (spot R11) and CML28-like (spot R80), and a 14-3-3 family protein GF14-B-like (spot R50). PYR1 was a newly found receptor for ABA that regulates plant stress responses. PYR1 level was increased by 2.7- and 5.2-fold change in the roots of wild wheat seedlings after 24 and 48 h of drought stress, respectively, compared with that of the control plants (S5 Table and Fig 4A). PYR1, PYR1-like protein (PYL), and a regulatory component of ABA receptor (RCAR) form a large family of ABA receptors. PYR1/PYL/RCAR1 binds ABA and interacts with type 2C protein phosphatases (PP2Cs) that negatively regulate ABA signaling, thereby blocking the phosphatase activity of PP2Cs in an ABA-dependent manner in vivo [42]. ABA signaling pathway plays a crucial role in plant response to various stresses, such as drought and salt stresses. We speculated that high abundance of cell-surface receptor PYRl along with increased ABA level in drought-stressed wild wheat roots resulted in the increase of cell sensitivity to turgor (water status) changes. This phenomenon regulated the plant’s rapid response and adaption to water deficiency. Cytosolic Ca^2+^ and proteinkinase/phosphorylase are the two major signal transduction pathways in plant cells, whereas Ca^2+^ and cAMP act as important secondary messengers, bind to their intracellular receptors, and activate protein kinase and protein phosphatase activities to conduct and amplify the signals through a series of cascade reactions. The levels of two Ca^2+^ receptors, namely, CML49-like isoform 1 (spot R11) and CML28-like (spot R80), were increased by 2.63- and 13.06-fold, respectively, in the roots of wild wheat seedlings at 24 h of drought stress (Fig 4A), thereby suggesting that the intracellular signal transduction system was greatly enhanced in the roots of wild wheat plants exposed to drought stress. 14-3-3 proteins can bind a large number of functionally diverse signaling proteins, including kinases, phosphatases, and transmembrane receptors, thereby indicating their pivotal roles in a wide range of vital regulatory processes, such as signal transduction regulation, apoptosis, and cell cycle control [43]. These proteins positively regulate H^+^-ATPase activity, which is known to initiate the stress responses by modulating the electro-chemical gradient across the plasma membrane [44]. The 14-3-3 protein family, including GF14a and GF14b in rice [44] and 14-3-3-like protein A in wheat, were abundant in roots exposed to high salinity [45]. In this study, the abundance of GF14-B-like protein (spot R50) was increased by 4.7- and 6.5-fold in roots of the plants exposed to drought stress for 24 and 48 h, respectively. This finding is consistent with the previous report on the effects of salt stress. By contrast, Rab GDP dissociation inhibitor (Rab GDI) (spot R24), was significantly decreased in abundance in the roots of drought-stressed plants (Fig 4A). The Rab GDIs constitute a family of small GTPases that serve a regulatory role in vesicular membrane traffic and play a critical role in the GTPase cycle of Rab/Ypt proteins by specifically binding GDP-bound Rabs and retaining them in the GDP-bound inactive state by inhibiting the release of GDP [46]. The down-regulation of RAB-GDI in the roots of drought-exposed plants suggested the induction of GTP, which triggered various transmembrane signaling systems.

However, only one signal transduction-related protein, the precursor of auxin-binding protein ABP20 (spot L35), was greatly up-regulated in the leaves of the wild wheat plants exposed to short-term drought stress (Fig 4A, S4 Table). The ABP20 contains a region that shared 40% amino acid identity with a putative auxin-binding site in ABP1, which is an auxin-binding protein isolated from maize coleoptiles [47]. ABP20 is localized within the cell wall and participates in signal transduction in the presence of abiotic stresses [48]. Increase in ABP20 gene expression level coincided with growth cessation [49]. The increased level of the precursor of auxin-binding protein ABP20 in this study might indicate active adaptation to water deficit conditions by leaf growth modulation, which requires sufficient water supplementation. Our physiological data showed that ABA was rapidly and significantly accumulated in the leaves of the wild wheat seedlings during the early stage of drought stress, whereas stomatal conductance was apparently decreased to reduce water evaporation (Fig 1 and S1 Table). These events also indicated active adaptation to drought stress. The significant increase in MDA contents was detected in the wild wheat seedlings at 48 h of drought stress. These findings provided new support to the point postulated in a recent review that the early stage of plant stress response (known as the alarm phase) is associated with the induction of stress-responsive signaling pathways and strong oxidative stress [41].

The proteins involved in signal transduction were greatly up-regulated at very early stages of plant stress response, thereby enhancing the activities of stress-responsive signaling pathways in the roots of the wild wheat plants exposed to drought stress. This very early protein-level response to water deficit in the roots of the wild wheat plants is consistent with the increase in ABA level and might contribute to its high drought tolerance.

### Antioxidation and defense-related protein levels were greatly increased in wild wheat leaves and roots in response to short-term drought stress

Drought stress activates the generation of ROS, which is required as a substrate and signal in cell metabolism, growth, and differentiation at low concentrations. However, ROS can also cause damage to macromolecules and membranes at high concentrations. To counteract the harmful effects of the ROS generated at early stages of plant stress response, various ROS scavengers were induced in wild wheat plants during drought stress. A total of 27 DEPs (corresponding to 26 unipros) involved in detoxification (antioxidation) and defense-related proteins, including 16 in the leaves and 11 in the roots, were detected in drought-stressed wild wheat seedlings (Fig 4B, S4 and S5 Tables). Nearly all of these proteins were significantly up-regulated with three exceptions, i.e., glyoxalase 1 (spot R16) and cytosolic glutathione peroxidase (spot R64) in roots, and universal stress protein (USP) family protein (spot L73) in leaves. These three proteins were greatly down-regulated in roots or leaves. It was unexpected that among the 26 DEP unipros, no common protein was observed in the leaves and the roots, thereby suggesting differences in detoxification and defense mechanisms underlying the plant’s response to drought in leaves and roots of the wild wheat. The glyoxalase 1 (spot R16) is a component of the glyoxalase system that carries out the detoxification of methylglyoxal and the other reactive aldehydes produced as a normal part of metabolism [50]. Glyoxalase 1 catalyzes the isomerization of the spontaneously formed hemithioacetal adduct between glutathione (GSH) and 2-oxoaldehydes (such as methylglyoxal) into S-2-hydroxyacyl glutathione [51]. Glyoxalase 1 was down-regulated by 2.5- and 2.2-fold in the roots of the wild wheat seedlings at 24 and 48 h of drought stress, respectively (Fig 4B), thereby suggesting the inhibition of normal metabolism and the initiation of the acclimation phase, which lasts several days and leads to the establishment of a new homeostasis in plant metabolism under stress [41]. Cytosolic glutathione peroxidase (spot R64) was down-regulated by 8.6- and 9.0-folds in the roots of the wild wheat seedlings at 24 and 48 h of drought stress, respectively (Fig 4B), thereby suggesting that GSH oxidation reaction was inhibited to maintain a high level of GSH in the cytoplasm of root cells. High levels of GSH are important for plants to protect macromolecules from ROS damage under stress conditions. The USP family protein (spot L73) in the leaves was down-regulated by 1.5-folds at 24 h of drought stress, but went back to the normal level at 48 h of drought stress (Fig 4B), its function remains unclear. Notably, all the up-regulated ROS scavengers described above mainly included two types, as follows: direct ROS scavenger, such as Cu/Zn superoxide dismutase (spot L38), heme-dependent peroxidases (spot L23), peroxiredoxin (spot L31, L32, L34 and L97), horseradish peroxidase (spot R49), and class III peroxidase (spot R85); and enzymes involved in non-enzymatic antioxidant generation, such as glutathione S-transferase (spot L25), thioredoxin H (spot L87), glutathione transferase F5 (spot L30), glutathione S-transferase-N (spot R54), thioredoxin-disulfide reductase (spot R67), ascorbate peroxidase (spot R77), GDP-mannose 3,5-epimerase 2-like (spot L59), and glutathione S-transferase (spot R83) (Fig 4B, S4 and S5 Tables). The level of ROS scavengers, such as ascorbate peroxidase 2-like protein and Cu/Zn superoxide dismutase in roots, increased in drought-tolerant sunflower (*Helianthus annuus L*.) inbred line, but decreased in the sensitive variant [52]. A pathogenesis-related protein (PRP10) (spot R55) was significantly induced in the roots of wild wheat plants under drought stress in the present study (Fig 4B). PRP10, which belongs to the SRPBCC (START/RHO_alpha_C/PITP/Bet_v1/CoxG/CalC) domain superfamily of proteins, binds the hydrophobic ligands and catalyzes the condensation of two emodin molecules to the bioactive naphthodianthrone hypericin. Furthermore, PRP10 performs enzymatic activities in plant secondary metabolism and plays a key role in abiotic stress response [53]. Another root protein, thaumatin-like protein TLP5 (spot R71), was over-accumulated under drought stress (Fig 4B). Plant TLPs are characterized as PRP family 5 (PRP5). TLP expression is induced by environmental stresses such as pathogen/pest attack [54] and drought [55] and cold stresses [56]. The up-regulation of these two proteins indicated that the pathogenesis defensive network was enhanced under drought stress.

Although differences in drought responses (antioxidation and defense) were observed in the leaves and roots of the wild wheat plants exposed to short-term drought stress, two common underlying mechanisms were effective in eliminating the over-accumulation ROS in the two organs. The first mechanism involved in non-enzymatic antioxidants, such as ascorbate and glutathione, whereas the second involved in the enzymatic pathways (superoxide dismutase, peroxidase, and ascorbate peroxidase). Additionally, in the roots, pathogenesis defense system was activated under drought stress.

### Photosynthesis-, carbon metabolism- and energy metabolism-related proteins greatly changed in wild wheat leaves and roots

**Fig 5:**
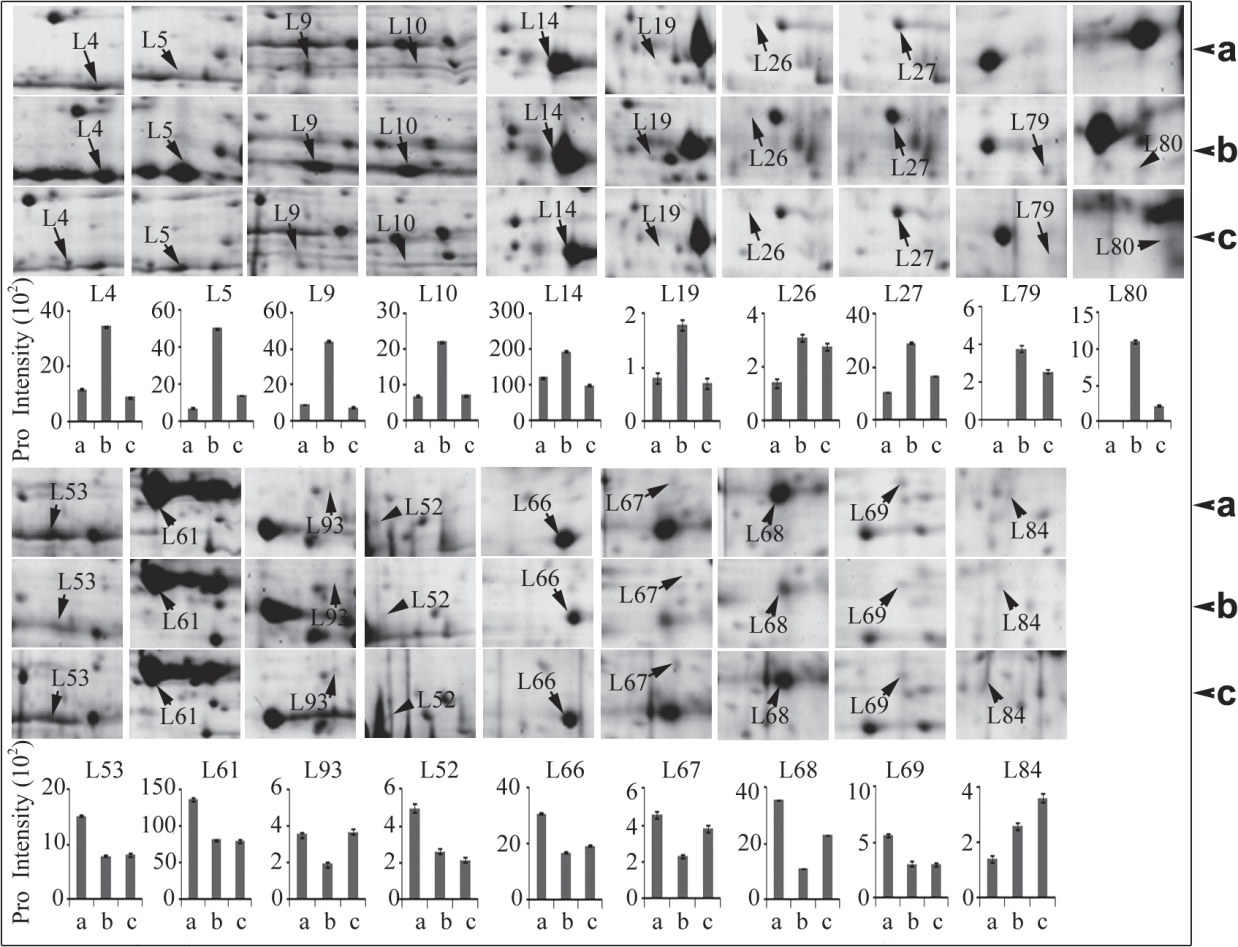
Differentially changed proteins involved carbon fixation in leaves of *T*. *boeoticum* plants exposed to 20% PEG6000 for 48 h to induce drought stress. All protein spots are enlarged from S1A–S1C Fig, and the protein intensity values are from S2 Table a, b, c represent 0, 24, and 48 h of drought treatment, respectively. Protein spot identities correspond to those in S1 Fig and S2 and S4 Tables. Error bars represent standard deviation (n = 3).

Photosynthesis is the primary pathway for the production of carbohydrates, which are essential for cell growth and proliferation. Photosynthesis is composed of two steps, namely, photoreaction (light dependent) and dark reaction (carbon fixation). The capability of a plant to maintain a stable photosynthetic rate is significant for sustaining plant growth under stress. In the present study, a total of 24 DEPs involved in photosynthesis were detected in wild wheat leaves, 16 of which were up-regulated and 8 were down-regulated (Fig 3A, S4 Table). Four DEPs (spots L39, L41, L80, and L96) were involved in photoreaction and were up-regulated by approximately 1.5- to 2.3-fold. These four DEPs were grouped into three types: ① thylakoid lumenal 16.5 kDa protein (TL16.5) (spot L39), and thylakoid membrane phosphoprotein 14 kDa (TMP14) (spot L41), which were increased in abundance by 1.8- and 2.3-fold at 24 h of drought stress, respectively. TMP14 was a novel phosphorylation subunit of photosystem I (PSI) reaction center complex and was probably involved in the interaction with light harvesting complexes [57]; ② a PSII oxygen-evolving enhancer protein 1 (spot L96), which was increased by 1.9-fold at 24 h of drought stress; ③ a Ferredoxin-NADP(H) oxidoreductase (FNR) (spot L80), which was localized in the thylakoid membrane, was detected after 24 and 48 h of drought stress (S4 Table). FNR catalyzes the reversible electron transfer between NADP (H) and ferredoxin (Fd), which is a rate-limiting step for photosynthesis, and resulting in the production of proton (H^+^) and electron. These results indicated that light-dependent reaction was enhanced in the leaves of wild wheat seedlings in response to short-term drought stress, thereby resulting in high amounts of ATP and NADPH. This result contradicts the point of a recent review [58], which postulated that photosynthetic electron transport chain was markedly suppressed during drought stress, and as a consequence, the excess excitation energy was driven toward the production of ROS, possibly revealing the strategy of the drought-tolerant wild wheat to actively cope with moderate drought stress at early stages. In addition, a total of 19 DEPs involved in carbon fixation (Calvin cycle) were detected in the leaves of the wild wheat seedlings under short-term drought stress (Fig 5). Eight of these 19 DEPs were down-regulated, including Rubisco large subunits (spot L52, L53 and L69), a glyceraldehyde 3-phosphate dehydrogenase (GAPDH, spot L68), a fructose-bisphosphate aldolase (FBPA, spot L66), a putative carbonic anhydrase (spot L67), and two Rubisco activase isoforms (spot L61 and L93). All these enzymes played crucial roles in the Calvin cycle. Rubisco, the rate-limiting enzyme of the Calvin cycle, is a bifunctional enzyme. Besides catalyzing the addition of CO_2_ to RuBP, with the formation of two molecules of glycerate 3-phosphate, Rubisco catalyzes the addition of oxygen to RuBP with the formation of one molecule of glycerate 3-phosphate and one molecule of glycollate 2-phosphate, thereby CO_2_ is as a competitive inhibitor of the oxygenase reaction, and O_2_ as a competitive inhibitor of the carboxylation reaction. As described above, five differentially changed protein spots (L4, L5, L10, L52, and L69) were identified as the RLS with different *Mr* (moleculear wight) and pI (Fig 5, S2 Fig and S4 Table). One spot (L52) was a posttranslational modification (such as phosphorylation) because the observed *M*r was larger than the theoretical values. The other four protein spots were fragmentation of the RLS because the observed *M*r was smaller than the theoretical values (S2 Fig and S4 Table). All RLS fragmentation isoforms detected in drought-tolerant wild wheat exhibited up-regulation and the posttranslational modification isoform down-regulation in response to drought stress at the early stage. This result was in agreement with the discovery of higher levels of RLS fragmentation isoforms in a drought-tolerant wild wheat genotype than in drought-sensitive genotype under drought stress [16]. Rubisco subunits, the most abundant proteins in leaf tissue, are reported to be susceptible to fragmentation under drought stress, possibly leading to isoforms of slightly different *Mr*/pI [59]. Rubisco fragmentation was also interpreted as a protein turnover in response to drought stress [60]. The principal role of Rubisco activase is to release inhibitory sugar phosphates, such as ribulose-1, 5-biphosphate, from the active sites of Rubisco leading to enzyme activation by CO_2_ (via carbamylation) [61]. Rubisco activase also plays the role of a chaperone during stress [62]. The Rubisco activase in the stroma proteins exhibited two isoforms (41kDa to 43 kDa and 45 kDa to 46 kDa), which arise from one alternatively spliced transcript. The larger Rubisco activase isoform contributes to the photosynthetic adaptation to mild heat stress in vivo and is modulated by the redox status in the stroma [63]. The smaller isoform mainly participates in the maintenance of Rubisco’s initial activity under non-stress conditions [64]. In this study, the down-regulation of the two isoforms of Rubisco activase (spot L61 and L93) in the leaves of wild wheat under short-term drought stress suggested the breakdown of the redox homeostasis and the suppressed photosynthesis. Our physiological analysis also showed that closure of stomata to reduce water loss simultaneously resulted in the reduction of CO_2_ assimilation in wild wheat plants exposed to short-term drought stress (Fig 1 and S1 Table). Our proteomic data were consistent with the data obtained from physiological analysis, which stated that the photosynthetic process was inhibited in the leaves of wild wheat under short-term drought stress. Such inhibition might be an active strategy of drought-tolerant wild wheat species to establish a new homeostasis in plant metabolism at the early stages of drought stress; the level of plant stress tolerance increases during acclimation phase [41].

**Fig 6:**
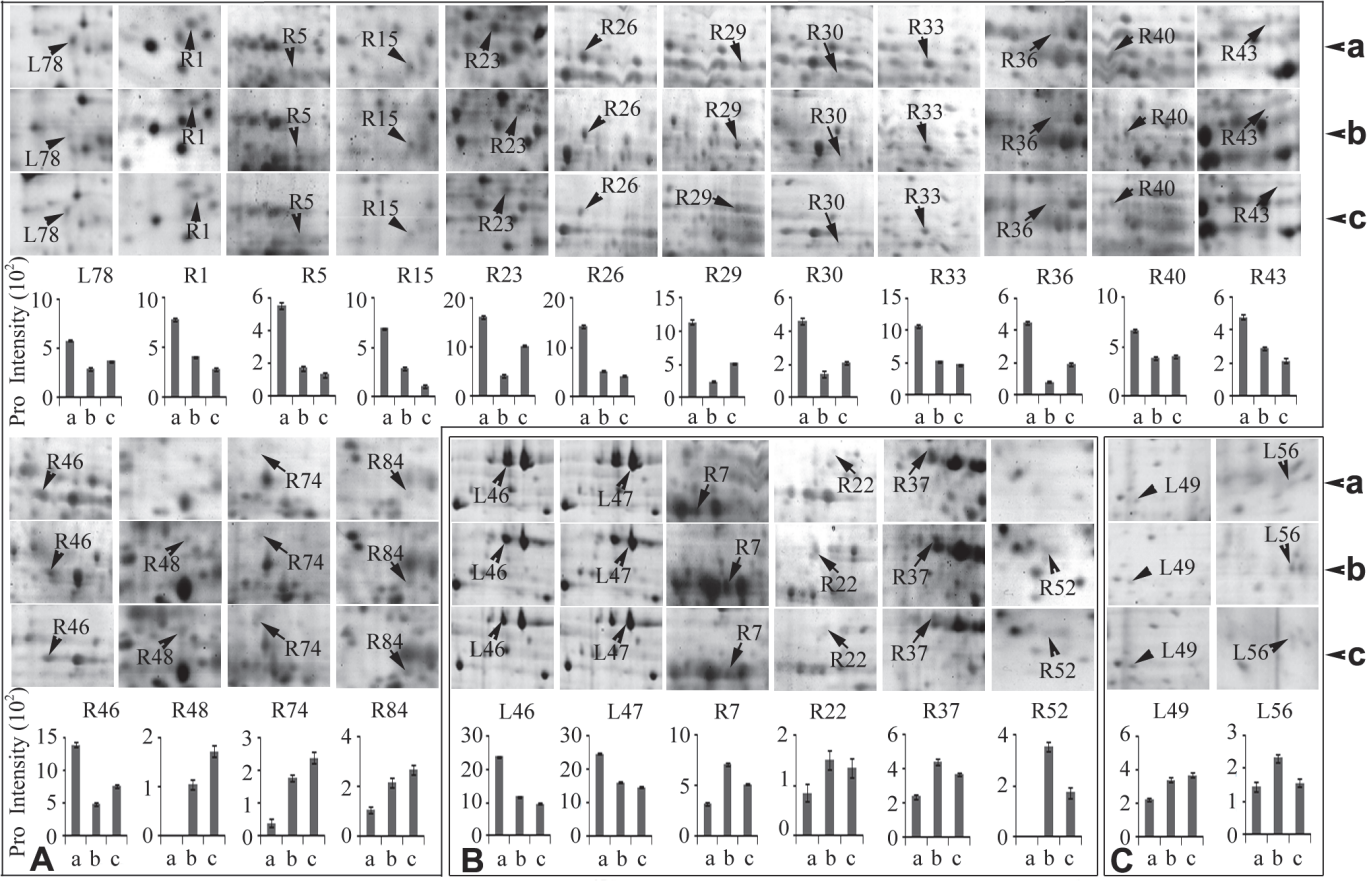
Differentially changed proteins (DEPs) involved carbon metabolism in the roots and the leaves of *T*. *boeoticum* plants exposed to 20% PEG6000 for 48 h to induce drought stress. A, DEPs associated with Embden-Meyerhof-Parnas pathway; B, DEPs associated with pentose phosphate pathway; C, DEPs associated with tricarboxylic acid cycle. All protein spots are enlarged from S1A–S1F Fig and protein intensity values are from S2 and S3 Tables. a, b, c represent 0, 24, and 48 h of drought treatment, respectively. Protein spot identities correspond to those in S1 Fig and S2, S3, S4, and S5 Tables. Error bars represent standard deviation (n = 3).

Besides photosynthesis, carbohydrate catabolism is among the processes that are likely to be most affected by drought stress. The glucose catabolism pathways include the following: Embden-Meyerhof-Parnas pathway (EMP pathway, also known as glycolysis) converts glucose into pyruvate and H^+^, and the free energy released in this process is used to form the high-energy compounds ATP and NADH; and the tricarboxylic acid cycle (TCA cycle), the second stage of cellular respiration by which living cells break down pyruvates (formed during the glycolytic breakdown of glucose) to CO_2_ and H_2_O in the presence of oxygen, with the concomitant production of energy essential for normal growth and differentiation; TCA cycle also plays a role in producing precursors for biosynthetic pathways [65]. Another parallel pathway of glucose catabolism, the pentose phosphate pathway (PPP pathway, also called the hexose monophosphate shunt or the phosphogluconate pathway), generates ribose 5-phosphate (5-carbon sugars) and NADPH supply with reducing power for biosynthesis metabolism. The PPP pathway is involved in oxidation of glucose, and its primary role is anabolic rather than catabolic [65]. In the present study, 15 DEPs involved in EMP pathway and 4 involved in PPP pathway were detected in the roots of wild wheat seedlings in response to short-term drought stress (Fig 6A and 6B). Twelve of the 15 DEPs related to EMP pathway were significantly down-regulated, including phosphoglucomutase (spot R26), pyrophosphate-dependent phosphofructokinase (spot R33), enolase (spot R5), triose-phosphate isomerase (spot R1), phosphoglycerate kinase (spot R23), and glyceraldehyde-3-phosphate dehydrogenase (spot R43), etc. however, 6-phosphofructokinase 2-like (spot R74) and fructose-bisphosphate aldolase (spot R48 and R84) were not down-regulated (Fig 6A), thereby suggesting the suppression of glycolysis in the roots of the wild wheat plants in response to short-term drought stress. This finding verified that in plants, the primary metabolism may be modulated to establish a new homeostasis. It was unexpected that no differentially changed protein (enzyme) involved in TCA cycle was detected in the roots exposed to short-term drought stress (Fig 6C), and this phenomenon may be due to the crucial role of this pathway in living organisms. Another surprise was that all four DEPs involved in PPP pathway, including UDP-glucose/GDP-mannose dehydrogenase (spot R7), transketolase (spot R22), transaldolase-like protein (spot R37), and ribulose-phosphate 3-epimerase (spot R52), were greatly increased in abundance in the roots of the wild wheat seedlings exposed to short-term drought stress. Thus, PPP pathway in the roots might be up-regulated in response to short-term drought stress. All these evidence suggest that the primary metabolism may be modulated to establish a new homeostasis in drought-stressed wild wheat roots. To the best of our knowledge, this is the first time that differentially changed proteins involved in EMP and PPP pathways in root tissue were identified in drought-stressed wheat plants on a large scale. The decrease in the levels of glycolytic enzymes and the increase in the levels of phosphogluconate pathway enzymes might be strategies for tolerant wild wheat to reduce energy consumption. These plants may also accumulate sugars as osmotic substrates in the roots to actively adapt to drought stress.

Unlike in root tissues, only 5 differentially changed proteins involved in glucose catabolism were detected in the leaves of the wild wheat plants subjected to short-term drought stress. Fructokinase (spot L78) and transketolase (spots L46 and L47) were significantly decreased, whereas succinate dehydrogenase (spot L49) and predicted citrate synthase 4 (spot L56) increased in abundance (Fig 6). Fructokinase catalyzes the transfer of a phosphate group from ATP (the substrate) to fructose as the initial step in its utilization, and this enzyme plays a main role in sucrose and fructose metabolism. The decrease in the level of this enzyme implies the inhibition of the sucrose and fructose utilization, which is important in maintaining soluble sugar concentration and water status in response to drought stress. Transketolase is an enzyme in both the PPP (in all organisms) and the Calvin cycle of photosynthesis. Transketolase catalyzes two important reactions that operate in opposite directions in these two pathways. The decrease in transketolase level suggested the suppression of the two pathways in the leaves of drought-stressed wild wheat plants. In contrast, TCA cycle-related enzymes, succinate dehydrogenase, and predicted citrate synthase 4 were increased in abundance in leaves of plants exposed to short-term drought stress (Fig 6C), possibly implying that the enhanced energy production was obtained through the TCA cycle in the leaves of drought-stressed wild wheat. Similar results were previously reported in the leaves of barley [66] and wheat [16].

The responses of root or leaf proteome to abiotic stress, such as drought stress [52, 67], salt stress [14], nitrate availability [68], and cadmium stress [69], have been reported in various plant species, including rice, soybean, sunflower, and maize. The contradictory results were concluded about the alteration of carbohydrate metabolism in various plant species under drought stress. Some researchers reported up-regulation in carbohydrate metabolism [66], whereas others reported down-regulation [67]. These conflicting results may possibly be due to different plant organs used or the different time courses of the drought treatments in different experiments. Results in the present study showed that the majority of the differentially changed enzymes involved in EMP pathway decreased in abundance in roots of the wild wheat under drought stress. This finding agreed with that reported in soybean roots subjected to short-term drought stress [67].

The alterations of carbohydrate metabolism were closely related to the changes of energy metabolism in plants under abiotic stress conditions. A total of seven DEPs related to energy metabolism were detected, including one (spot L85) in the leaves and 6 (spot R3, R4, R27, R72, R79, R81) in the roots of wild wheat seedlings exposed to short-term drought stress (S4 and S5 Tables). Among these DEPs, two isoforms of ATP synthase beta subunit (spot R3 and R27) and an ATP synthase CF1 beta subunit (spot R4) showed greatly decreased expression level. In accordance with these results, Huseynova et al. [70] also found the decreased abundance of ATP synthase beta subunit in wheat cultivars Giymatli-2/17 (drought-sensitive) and Azamatli-95 (drought-tolerant) under water stress. ATP synthase F1-beta-subunit is a specific calcium-binding protein in the mitochondria; this protein coordinates ATP production with the demand for ATP-fueled calcium pump activity and regulates cytosolic calcium concentration [71]. In contrast, the abundance of respiratory-chain NADH dehydrogenase 24 Kd subunit (spot R72), mitchondrial-like ATP synthase subunit d (spot R79), and adenosine kinase 2-like (spot R81 and L85) were significantly increased at 24 and 48 h of drought stress. NADH dehydrogenase (ubiquinone) is located in the inner mitochondrial membrane and is one of the “entry enzymes” of oxidative phosphorylation in the mitochondria that transfer electrons from NADH to coenzyme Q10 [72]. NADH dehydrogenase can translocate four protons across the inner membrane per molecule of oxidized NADH, thereby contributing to the establishment of the electrochemical potential used to produce ATP [73]. Our results suggested the following aspects: the occurrence of complex changes of energy metabolism in wild wheat plants in response to short-term stress; and the establishment of a new homeostasis in root tissues under drought stress.

The comparison of the DEPs related to carbon metabolism and energy metabolism between the leaves and the roots showed only five common unipros (corresponding to 10 identities), each with similar change patterns (S6 Table). This result suggested the occurrence of different primary metabolic responses to short-term drought stress in the two organs.

In general carbohydrate catabolism (glycolysis) was greatly down-regulated, but the PPP was enhanced in the roots of the drought-stressed wild wheat plants. In contrast, photosynthesis was down-regulated in the leaves, concomitant with the occurrence of complex changes of energy metabolism and the establishment of a new homeostasis.

### Amino acid and protein metabolism-related proteins were largely altered in wild wheat roots and leaves

**Fig 7:**
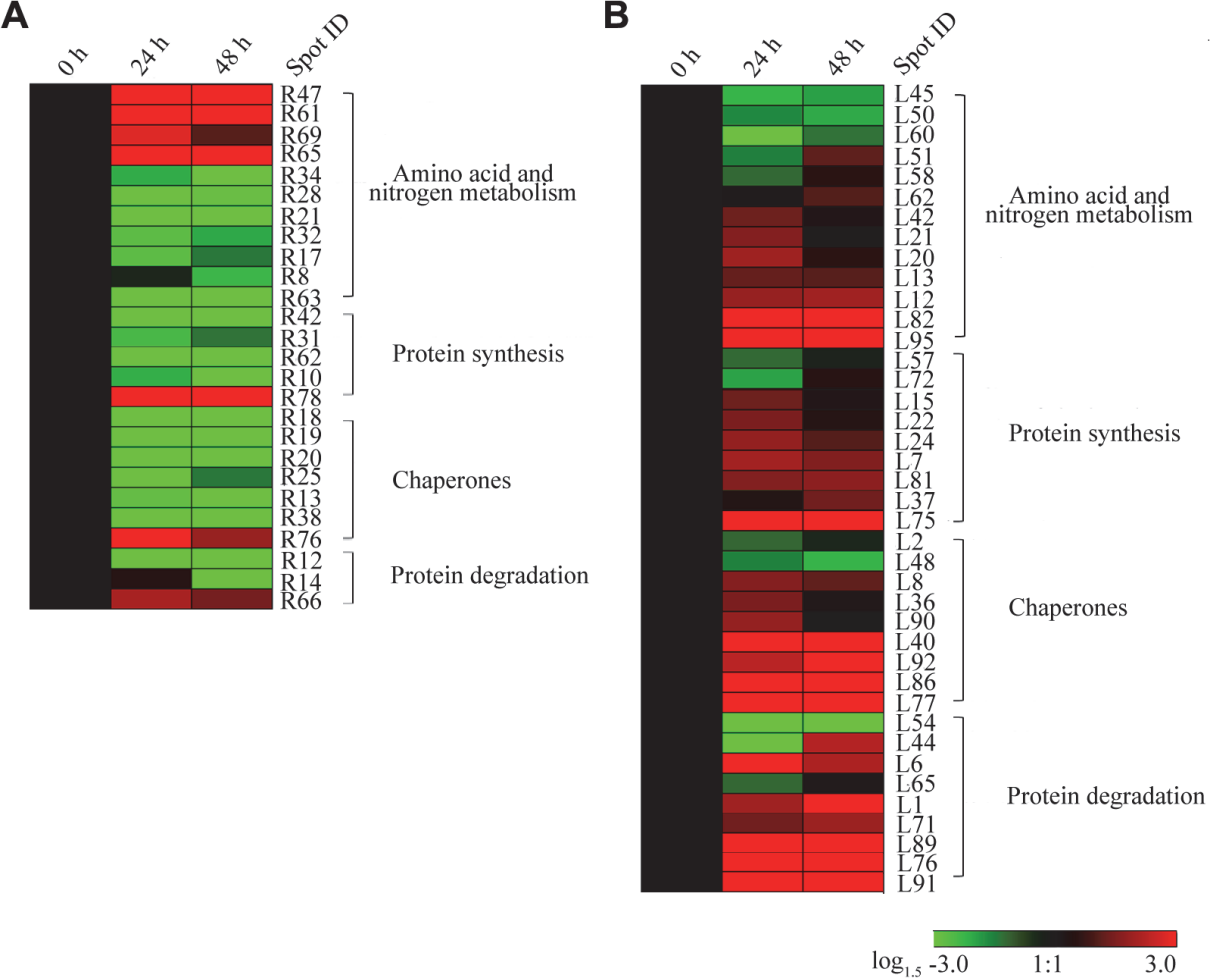
Hierarchical clustering of drought stress-responsive proteins associated with amino acid and protein metabolism in *T*. *boeoticum* roots (A) and leaves (B). A hierarchical cluster analysis was conducted using the Genesis software package version 8.2 1.7.6 (Graz University of Technology, Austria, http://genome.tugraz.at/genesisclient/genesisclient_download.shtml) and the log_1.5_-transformed values of fold-change ratios listed in S7 Table. Protein spot identities correspond to those in S1 Fig and S2, S3, S4, and S5 Tables. The annotations of individual proteins are listed in S4 and S5 Tables.

The metabolism of amino acids and proteins is important in plants, and it is most affected by drought stress besides photosynthesis and carbohydrate metabolism. In the present study, 40 DEPs in leaves and 26 in roots were involved in amino acid and protein metabolism (S4 and S5 Tables). These proteins were separately classified into four groups according to their biological functions, as follows: amino acid and nitrogen metabolism, protein synthesis, chaperone (protein folding), and protein degradation (Fig 7A and 7B). Most of the DEPs in root tissue were down-regulated under drought stress, thereby suggesting the inhibition of the protein metabolism (Fig 7A). Despite that, some of these proteins showed increased levels, such as glutamate decarboxylase (spot R65), proteasome subunit beta type-7-A-like (spot R66), and heat shock protein (Hsp) 70 (spot R76). Glutamate decarboxylase plays a critical role in the decarboxylation of glutamate to produce *γ*-aminobutyrate (GABA) and CO_2_, and GABA was involved in redox homeostasis, energy production, and carbon/nitrogen balance in plants under biotic and abiotic stresses [74]. The increase in abundance of glutamate decarboxylase probably suggested that GABA pathway was enhanced to establish a new homeostasis in wild wheat roots under drought stress. Heat-shock proteins (Hsps) are involved in various intracellular processes and play important roles in protein–protein interactions, folding, assembly, intracellular localization, secretion, transport, prevention of protein aggregation and degradation, and reactivation of damaged proteins [75]. Hsps were increased by drought stress in wheat leaves [76] and rice roots [77]. The increased expression of Hsp70 under drought stress in this study is in line with previous results.

Conversely, nearly all of the DEPs in the last three groups (protein synthesis, chaperone, and protein degradation) were increased in abundance in the leaves of the wild wheat plants under drought stress (Fig 7B), thereby confirming the enhanced protein metabolism. It was recently reported that in the group of protein destination and storage proteins, Hsp90, Hsp70, ATP-dependent Clp protease, and a protein disulfide isomerase were up-regulated by drought stress in the leaves of a tolerant barley accession [66]. This metabolic drought-response mechanism may be consequently followed by plant modulation in expression level of proteins involved in osmoprotector and osmoregulator synthesis, such as proline. As an adaption to drought, plants may increase their accumulation of amino acids and amines, which function as osmoprotector and osmoregulator. It was showed that the expression levels of some proteins related to amino acid biosynthesis processes, such as plastid glutamine synthetase (GS) 2 (spot L12 and L13), cysteine synthase A (spot L21), beta-cyanoalanine synthase (spot L85), and ornithine carbamoyltransferase (spot L95), were increased under drought stress (Fig 7B). Among these proteins, GS serves a key function in nitrogen metabolism and is implicated in the regulation of proline levels in plants. GS participates in the combination of ammonia with glutamate to yield glutamine at the expense of ATP. Our physiological data also showed that the proline level was significantly increased in the leaves of the wild wheat seedlings exposed to water stress. Two different S-adenosyl methionine (SAM) synthases were identified, SAM synthase 3 (spot L62 and L58) was up-regulated, whereas SAM synthase 1 (spot L60) was down-regulated (Fig 7B).

Protein metabolism was down-regulated in the roots, but enhanced in the leaves of the wild wheat seedlings in response to short-term drought stress. The metabolism of amino acids and nitrogen was also differentially modulated in the two organs under drought stress.

### Other functional and unknown proteins

Actins are essential components of cell cytoskeleton and participate in cytoplasmic streaming, cell shape determination, cell division, organelle movement, and extension growth, implicating in the regulation of tip growth of soybean root hairs [78]. In the present study, the abundance of Actin-53 (spot R59) was induced in the roots of wild wheat seedlings exposed to short-term drought stress, thereby indicating that root cell cytoskeleton was altered to actively adapt to the water deficit condition. Moreover, four DEPs in the leaves and 8 in the roots were identified as unknown or hypothetical proteins (S4 and S5 Tables). These proteins might be new drought-related proteins and should be studied further. These proteins may represent the novel mechanisms for drought tolerance in wild wheat.

## Conclusion

In this study, physiological changes and the proteome alterations were determined in the roots and the leaves of the control and the drought-treated wild wheat seedlings to reveal proteomic responses to short-term drought stress in the two organs. The physiological data indicated that the ABA levels were greatly increased in the both organs of the drought-treated plants, however, the increase was much rapid and greater in the leaves than in the roots. The net photosynthetic rate of the wild wheat leaves was significantly decreased under short-term drought stress. The deleterious effect of drought on the studied traits mainly targeted photosynthesis. Comparative proteomic analysis identified 98 and 85 differently changed protein spots (corresponding to 87 and 80 unipros, respectively) in the leaves and in the roots, respectively. Totally, 18 identities (6 unipros) were common in the both organs, thereby indicating the occurrence of tissue-specific responses to drought stress at protein level. The different drought-response proteins between the roots and the leaves of *T*. *boeoticum* seedlings exposed to short-term drought stress were identified on a large scale. The identified DEPs in the leaves and the roots were classified into 15 categories: detoxification and defense, carbon metabolism, amino acid and nitrogen metabolism, proteins metabolism, chaperones, transcription and translation-associated proteins, photosynthesis, nucleotide metabolism, signal transduction-associated proteins, lipid metabolism, energy metabolism, cell wall metabolism, cell membrane development, cytoskeleton, and cytokinesis-associated proteins. An impressive 86% of DEPs were implicated in the first eight functional groups. Further analysis revealed that leaves and roots exhibited some mutual and tissue-specific responses to short-term drought. The levels of antioxidation and defense-related proteins were greatly increased in both the roots and the leaves of wild wheat seedlings under short-term drought stress. Signal sensing and transduction-associated proteins were greatly up-regulated in the roots. Photosynthesis and carbon fixation ability were decreased in the leaves. Glycolysis was down-regulated but PPP pathway was enhanced in the roots, thereby resulting in the occurrence of complex changes in energy metabolism and the establishment of a new homeostasis. Protein metabolism was down-regulated in the roots, but enhanced in the leaves of wild wheat seedlings under short-term drought stress. Amino acid and nitrogen metabolism was also differentially modulated in the roots and the leaves under drought stress. To the best of our knowledge, this is the first proteomic investigation on the drought responses in the roots and the leaves of drought tolerant *T*. *boeoticum*. These results may contribute to the existing knowledge on the complexity of root and leaf protein changes that occur in response to drought. Our results will provide a framework for further functional studies on each identified protein.

## Supporting Information

S1 Fig Representative 2-DE gels of proteins in the leaves (A to C) and the roots (D to F) of *T*. *boeoticum* plants exposed to 20% PEG6000 for 48 h to induce drought stress. A and D, 0 h of drought treatment; B and E, 24 h; C and F, 48 h. Total proteins were extracted by the TCA–acetone precipitation method and separated by IEF/SDS-PAGE. Proteins were stained with Coomassie Brilliant Blue G-250 (leaf proteins) or silver-staining (root proteins). Protein samples (600 μg from leaves or 250 μg from roots) were loaded onto pH 4 to 7 IPG strips (17 cm, linear). SDS-PAGE was performed with 12% and 11.25% (for leaf and root proteins, respectively) gels. A total of 115 differentially changed protein spots in the leaves and 102 in the roots are numbered. Spot identities with ‘L’ and ‘R’ represent the spot from leaf and root samples, respectively. The spot identities correspond to those in S2, S3, S4 and S5 Tables.
(TIF)

S2 Fig Close-up of possible isoforms detected by 2-DE of proteins in the leaves and the roots of *T*.*boeoticum* plants exposed to 20% PEG6000 for 48 h to induce drought stress. A and B, leaf proteins at 24 h and 48 h of drought-treatment, respectively; C and D, root proteins at 0 and 24 h of drought-treatment. A total of 19 and 10 differentially changed protein spots corresponding to 8 and 5 unique proteins in the leaves and the roots, respectively, are shown. The spot identities correspond to those listed in S1 Fig, S4 and S5 Tables. RLS represent ribulose-1,5-bisphosphate carboxylase/oxygenase large subunit.
(TIF)

S3 Fig Pie chart of functional classification of all the differentially changed proteins (DEPs) in the leaves and the roots of T. *boeoticum* plants treated with 20% PEG6000 for 48 h. All the DEPs fifteen protein groups were categorized in to fifteen groups.
(TIF)

S1 Table Physiological measurements of wild wheat (*T*. *boeoticum*) plants exposed to 1/2 Hoagland solution containing 20% PEG6000 (-0.6 Mpa) for 48 h to induce drought stress. (DOC)

S2 Table The intensity of differentially changed protein spots (DEPs) in the leaves of *T*. *boeoticum* plants at different time points of drought stress. (DOC)

S3 Table The intensity of differentially changed protein spots (DEPs) in the roots of *T*. *boeoticum* plants at different time points of drought stress. (DOC)

S4 Table Differentially changed proteins (DEPs) in the leaves of wild wheat (*T*. *boeoticum*) plants under drought stress, as identified by MALDI-TOF-TOF. (DOC)

S5 Table Differentially changed proteins (DEPs) in the roots of wild wheat (*T*. *boeoticum*) plants under drought stress, as identified by MALDI-TOF-TOF. (DOC)

S6 Table The common differentially changed proteins between the leaves and the roots of the wild wheat (*T*. *boeoticum*) plants exposed to 20% PEG6000 for 48 h to induce drought stress. (DOC)

S7 Table Log_1.5_-transformed values of fold-change ratios of differentially changed proteins associated with amino acid and protein metabolism. (DOC)
