# Supplementary figures and images for: Physiological and Comparative Proteomic Analysis Reveals Different Drought Responses in Roots and Leaves of Drought-Tolerant Wild Wheat (Triticum boeoticum)
Source: PLoS One. 2015 Apr 10;10(4):e0121852. doi: 10.1371/journal.pone.0121852 (PMC4393031; doi:10.1371/journal.pone.0121852)

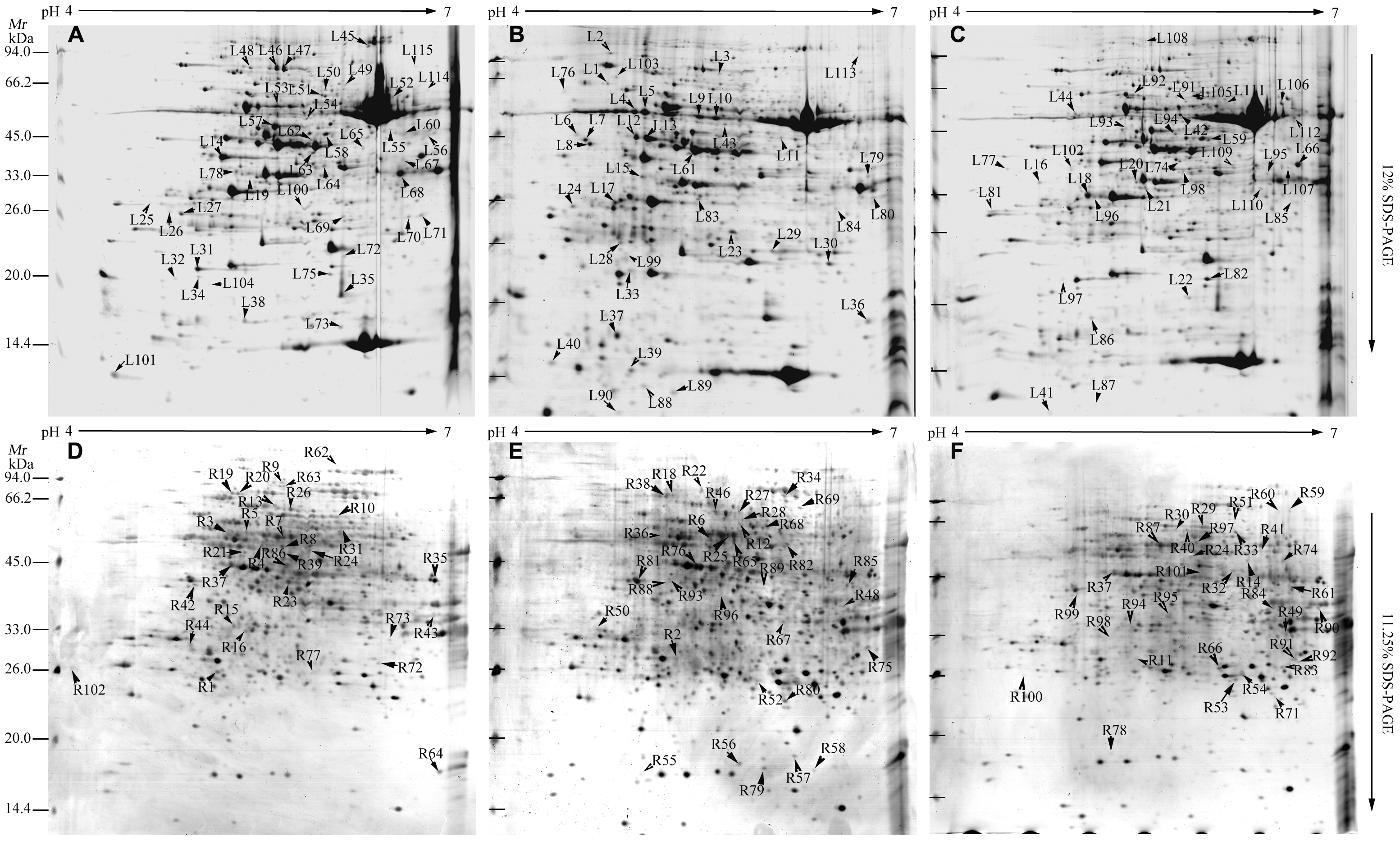

Supplement: S1 Fig — A and D, 0 h of drought treatment; B and E, 24 h; C and F, 48 h. Total proteins were extracted by the TCA–acetone precipitation method and separated by IEF/SDS-PAGE. Proteins were stained with Coomassie Brilliant Blue G-250 (leaf proteins) or silver-staining (root proteins). Protein samples (600 μg from leaves or 250 μg from roots) were loaded onto pH 4 to 7 IPG strips (17 cm, linear). SDS-PAGE was performed with 12% and 11.25% (for leaf and root proteins, respectively) gels. A total of 115 differentially changed protein spots in the leaves and 102 in the roots are numbered. Spot identities with ‘L’ and ‘R’ represent the spot from leaf and root samples, respectively. The spot identities correspond to those in S2, S3, S4 and S5 Tables. (TIF) [file pone.0121852.s001.tif]

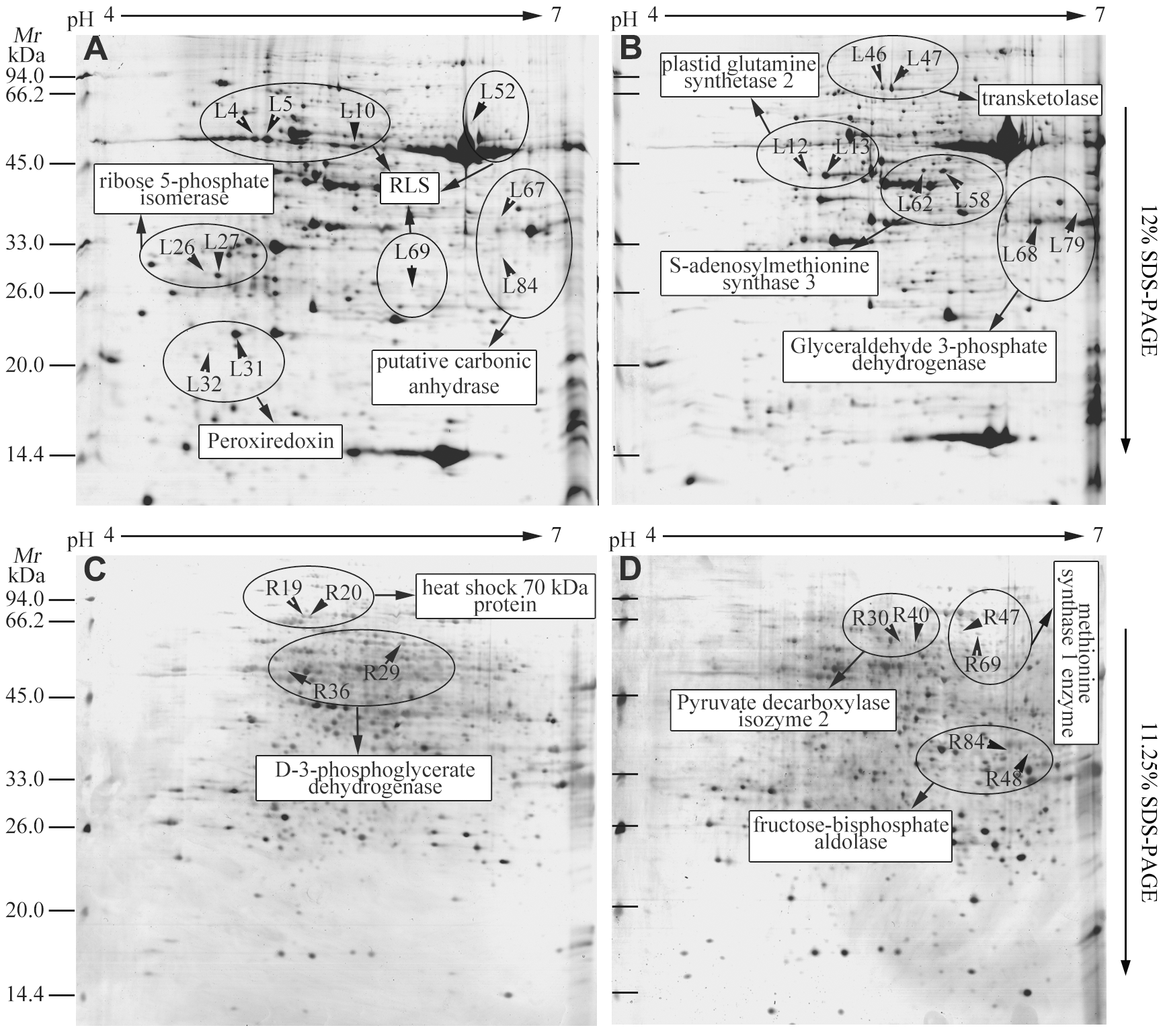

Supplement: S2 Fig — A and B, leaf proteins at 24 h and 48 h of drought-treatment, respectively; C and D, root proteins at 0 and 24 h of drought-treatment. A total of 19 and 10 differentially changed protein spots corresponding to 8 and 5 unique proteins in the leaves and the roots, respectively, are shown. The spot identities correspond to those listed in S1 Fig, S4 and S5 Tables. RLS represent ribulose-1,5-bisphosphate carboxylase/oxygenase large subunit. (TIF) [file pone.0121852.s002.tif]

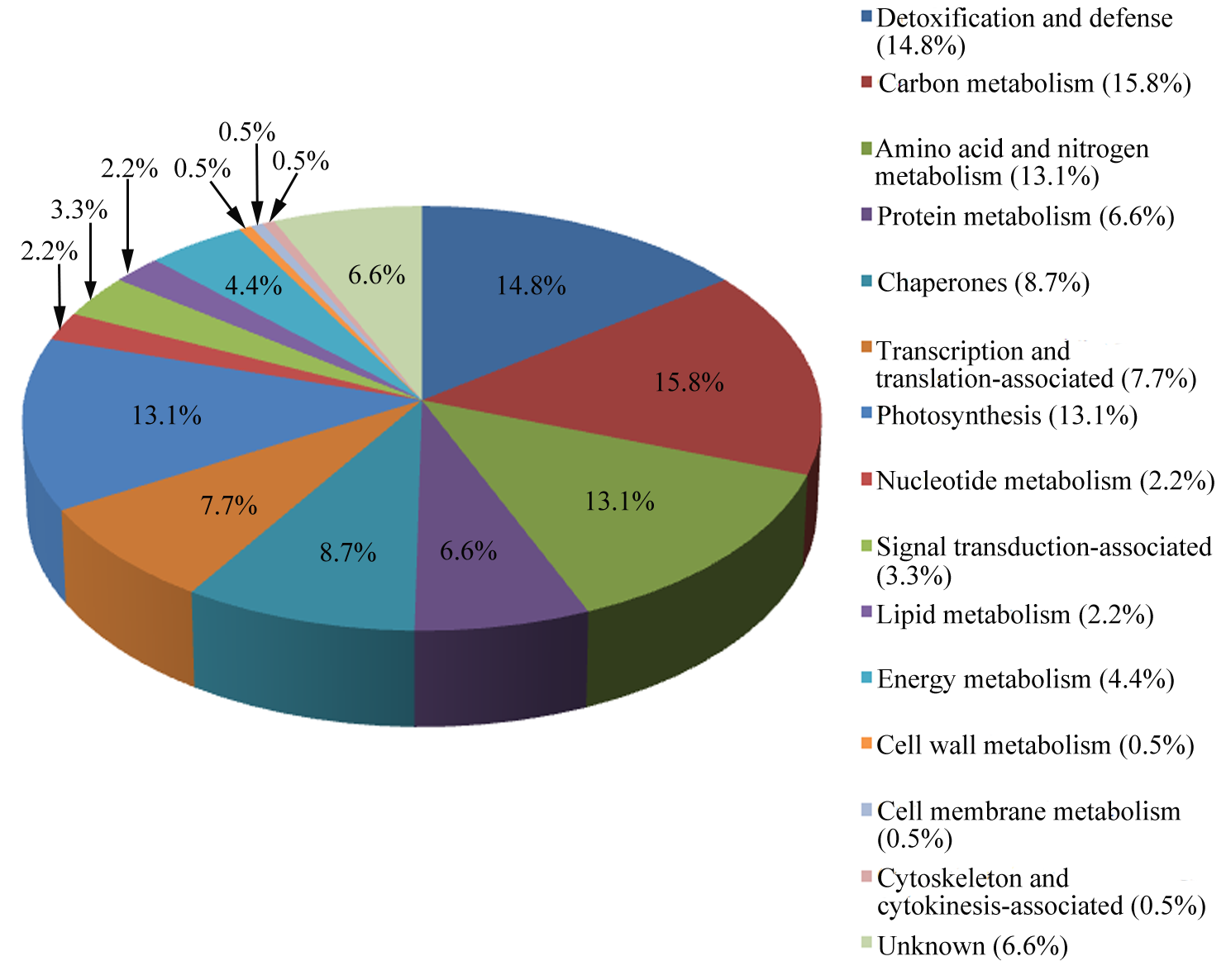

Supplement: S3 Fig — All the DEPs fifteen protein groups were categorized in to fifteen groups. (TIF) [file pone.0121852.s003.tif]
