# Supplementary material for: Physiological and Comparative Proteomic Analysis Reveals Different Drought Responses in Roots and Leaves of Drought-Tolerant Wild Wheat (Triticum boeoticum)
Source: PLoS One. 2015 Apr 10;10(4):e0121852. doi: 10.1371/journal.pone.0121852 (PMC4393031; doi:10.1371/journal.pone.0121852)
Supplement: S1 Table — (DOC) [file pone.0121852.s004.doc]

| **Indices^＊^** | **Time courses of drought-treatment^§^** | | | |  |
| --- | --- | --- | --- | --- | --- |
|  | 0 h | 24 h | 48 h |  | |
| **RWC in leaf ( %)** | 97.24^a^±0.61 | 95.37^b^±0.59 | 90.78^c^±0.31 | | |
| **RWC in root ( %)** | 95.01^a^±0.49 | 86.01^b^±0.73 | 82.03^c^±0.41 | | |
| **Soluble sugar in leaf (mg•g^-1^ FW)** | 32.23^c^±0.72 | 34.53^b^±0.70 | 47.26^a^±1.14 | | |
| **Soluble sugar in root (mg•g^-1^ FW)** | 11.85^c^±0.49 | 25.41^b^±0.72 | 34.35^a^±1.08 | | |
| **Proline content in leaf (μg•g^-1^ FW)** | 23.69^c^±0.62 | 29.14^b^±0.68 | 41.94^a^±0.58 | | |
| **Proline content in root (μg•g^-1^ FW)** | 16.33^b^±0.37 | 16.51^b^±0.40 | 32.27^a^±0.51 | | |
| **MDA in leaf**  **(****μmol•g^-1^ FW)** | 0.017^b^±0.001 | 0.018^b^±0.001 | 0.025^a^±0.002 | | |
| **MDA in root**  **(μmol•g^-1^ FW)** | 0.030^b^±0.001 | 0.031^b^±0.001 | 0.037^a^±0.001 | | |
| **ABA content in leaf**  **(μg.g^-1^ FW)** | 0.093^b^±0.009 | 0.32^a^±0.011 | 0.33^a^±0.010 | | |
| **ABA content in root**  **(μg.g^-1^ FW)** | 0.050^c^±0.007 | 0.087^b^±0.008 | 0.14^a^±0.007 | | |
| **Chlorophyll a**  **(mg•g^-1^ FW)** | 1.53^a^±0.01 | 1.47^b^±0.02 | 1.31^c^±0.02 | | |
| **Chlorophyll b**  **(mg•g^-1^ FW)** | 0.51^a^±0.02 | 0.46^b^±0.03 | 0.39^c^±0.03 | | |
| **Photosynthetic rate (mmol•m^-2^•s^-1^)** | 15.77^a^±1.64 | 6.39^b^±0.82 | 2.97^c^±1.38 | | |
| **Transpiration rate (mmol•m^-2^•s^-1^)** | 3.42^a^±0.24 | 0.75^b^±0.16 | 0.25^c^±0.08 | | |
| **Intercellular CO_2_ concentration (μmol•mol^-1^)** | 739.91^a^±28.71 | 568.00 ^b^±22.42 | 275.50^c^±62.42 | | |
| **Stomatal Conductance (mmol•m^-2^•s^-1^)** | 0.14^a^±0.03 | 0.031^b^±0.01 | 0.011^c^±0.00 | | |

**S1 Table.** Physiological and chemical measurements of wild wheat (*Triticum boeoticum*) exposure to 20% PEG6000 for 48 h of drought-treatment

- RWC, Relative water content; FW, Fresh weight; MDA, Malondialdehyde; ABA, Abscisic acid.

**^§^**mean values with the same letters in the same indices indicate non-signiﬁcant difference, and means with different letters show signiﬁcant difference at *P*<0.05 level.
