# Supplementary material for: Physiological and Comparative Proteomic Analysis Reveals Different Drought Responses in Roots and Leaves of Drought-Tolerant Wild Wheat (Triticum boeoticum)
Source: PLoS One. 2015 Apr 10;10(4):e0121852. doi: 10.1371/journal.pone.0121852 (PMC4393031; doi:10.1371/journal.pone.0121852)
Supplement: S2 Table — (DOC) [file pone.0121852.s005.doc]

**S2 Table.** The intensity of differentially changed protein spots (DEPs) in the leaves of *Triticum boeoticum* plants at different time point of drought-treatment.

| **Spot ID^※^** | **Experimental** | | |  | | **Spots intensity** | | | | | |  |
| --- | --- | --- | --- | --- | --- | --- | --- | --- | --- | --- | --- | --- |
|  | **Mr(kDa)** | **pI** | |  | | **0 h** | | **24 h** | | **48 h** | |  |
| **up regulated 1.5-2 times** | | | | | | | | | | | |  |
| **L8** | 42.73 | 4.70 | |  | | 1538.70±21.25 | | 2904.40±23.43 | | 2465.03±20.89 | |  |
| **L11** | 44.00 | 6.17 | |  | | 358.60 ±13.23 | | 707.50 ±16.12 | | 550.03 ±19.85 | |  |
| **L13** | 43.89 | 5.13 | |  | | 7646.53±34.15 | | 12444.33±41.52 | | 11823.00±43.67 | |  |
| **L14** | 40.04 | 5.11 | |  | | 12238.60±31.41 | | 19536.87±25.35 | | 9998.93±21.25 | |  |
| **L15** | 36.60 | 5.09 | |  | | 446.63±12.45 | | 752.23±17.71 | | 492.07±13.50 | |  |
| **L18** | 33.33 | 4.99 | |  | | 3661.23±27.23 | | 3474.53±21.56 | | 7106.90±29.55 | |  |
| **L21** | 35.59 | 5.40 | |  | | 1230.17±31.13 | | 2310.40±32.58 | | 1188.63±26.52 | |  |
| **L22** | 32.73 | 6.28 | |  | | 1044.80±11.32 | | 1894.10±19.25 | | 1226.03±21.64 | |  |
| **L23** | 27.09 | 5.80 | |  | | 1072.97±14.32 | | 2036.57±24.21 | | 976.20±18.75 | |  |
| **L30** | 23.43 | 6.54 | |  | | 1200.10±14.36 | | 2004.37±19.83 | | 2425.93±11.12 | |  |
| **L31** | 22.43 | 4.92 | |  | | 1547.77±26.66 | | 2797.93±31.18 | | 2358.37±25.51 | |  |
| **L33** | 22.76 | 5.00 | |  | | 304.30±8.75 | | 569.97±11.47 | | 1229.27±21.13 | |  |
| **L36** | 18.34 | 6.83 | |  | | 4856.30±26.70 | | 8774.23±29.01 | | 5264.10±21.89 | |  |
| **L37** | 17.23 | 4.90 | |  | | 3019.40±21.25 | | 3420.07±32.50 | | 5192.03±31.95 | |  |
| **L38** | 16.87 | 5.28 | |  | | 923.28±25.72 | | 1841.14±29.01 | | 1524.41±42.07 | |  |
| **L39** | 14.77 | 5.01 | |  | | 868.07±20.13 | | 1590.97±24.61 | | 1367.93±22.58 | |  |
| **L42** | 50.39 | 5.72 | |  | | 1143.03±21.09 | | 1926.00±21.13 | | 1262.83±15.62 | |  |
| **L43** | 49.67 | 5.66 | |  | | 536.13±21.12 | | 917.33±24.98 | | 869.53 ±21.76 | |  |
| **L49** | 59.73 | 6.02 | |  | | 226.00±12.97 | | 421.73±14.12 | | 467.83±14.32 | |  |
| **L56** | 43.72 | 6.80 | |  | | 145.33 ±15.86 | | 231.30 ±12.12 | | 157.17 ±11.35 | |  |
| **L62** | 43.60 | 5.77 | |  | | 194.07±12.17 | | 203.13±22.43 | | 296.17±15.15 | |  |
| **L63** | 37.01 | 5.88 | |  | | 813.43 ±16.24 | | 1307.47 ±25.89 | | 1385.67 ±25.15 | |  |
| **L70** | 26.05 | 6.63 | |  | | 326.70 ±16.88 | | 315.67±18.46 | | 499.30 ±19.02 | |  |
| **L71** | 26.03 | 6.70 | |  | | 867.77 ±34.33 | | 1494.03 ±25.19 | | 1856.83 ±30.17 | |  |
| **L81** | 31.01 | 4.42 | |  | | 662.50±12.95 | | 1238.88±18.62 | | 1315.50±21.98 | |  |
| **L84** | 31.70 | 6.59 | |  | | 139.3 ±2.32 | | 259.0 ±21.15 | | 363.5 ±15.98 | |  |
| **L94** | 45.47 | 5.72 | |  | | 297.57±17.79 | | 485.18±12.31 | | 516.07±15.24 | |  |
| **L96** | 32.86 | 5.05 | |  | | 1107.77±22.17 | | 2112.97±31.63 | | 1684.20±25.69 | |  |
| **L99** | 24.44 | | 4.97 | |  | | 275.30±18.34 | | 431.97±19.58 | | 470.83±20.28 | |
| **L100** | 28.57 | | 5.71 | |  | | 392.67±16.87 | | 717.13±23.21 | | 728.00±25.64 | |
| **up regulated 2-5 times** | | | | | | | | | | | |  |
| **L1** | 62.71 | 4.83 | |  | | 173.07±19.31 | | 378.67±10.17 | | 586.97±14.32 | |  |
| **L10** | 50.39 | 5.72 | |  | | 695.33±12.01 | | 2214.43±11.25 | | 708.37±15.34 | |  |
| **L4** | 51.80 | 5.03 | |  | | 1177.17±22.32 | | 3462.43±32.34 | | 898.60±16.64 | |  |
| **L7** | 43.55 | 4.67 | |  | | 751.03±21.27 | | 1671.57±23.42 | | 1402.40±22.56 | |  |
| **L9** | 50.12 | 5.53 | |  | | 946.87±15.42 | | 4447.97±29.09 | | 774.70±18.23 | |  |
| **L12** | 44.14 | 5.04 | |  | | 557.47±9.14 | | 1161.77±20.40 | | 1227.67±21.78 | |  |
| **L16** | 35.55 | 4.71 | |  | | 126.40±5.02 | | 411.90±10.21 | | 616.87±16.27 | |  |
| **L17** | 32.25 | 4.89 | |  | | 580.80±7.12 | | 2498.57±22.35 | | 498.83±10.09 | |  |
| **L19** | 35.08 | 5.29 | |  | | 81.53±16.03 | | 179.13±19.87 | | 71.26±13.25 | |  |
| **Spot ID^※^** | **Experimental** | | |  | | **Spots intensity** | | | | | |  |
|  | **Mr(kDa)** | **pI** | |  | | **0 h** | | **24 h** | | **48 h** | |  |
| **L20** | 34.97 | 5.33 | |  | | 1120.20±18.82 | | 2438.97±27.09 | | 1385.77±23.47 | |  |
| **L24** | 31.52 | 4.57 | |  | | 1057.1±13.22 | | 2165.2±21.44 | | 1621.7±21.89 | |  |
| **L26** | 28.38 | 4.73 | |  | | 142.23±14.68 | | 309.86±12.17 | | 279.72±12.32 | |  |
| **L27** | 28.15 | 4.81 | |  | | 1062.73±29.87 | | 2931.60±28.79 | | 1673.67±27.91 | |  |
| **L28** | 25.62 | 4.93 | |  | | 394.67±14.89 | | 824.73±18.90 | | 637.97±10.72 | |  |
| **L29** | 23.9 | 6.01 | |  | | 225.93 ±14.13 | | 601.23 ±18.24 | | 305.10 ±19.42 | |  |
| **L32** | 21.32 | 4.76 | |  | | 104.30±19.17 | | 374.27±18.37 | | 629.27±23.23 | |  |
| **L34** | 21.55 | 4.92 | |  | | 254.47±19.48 | | 999.27±19.72 | | 528.47±19.91 | |  |
| **L35** | 18.61 | 6.05 | |  | | 1087.27±12.02 | | 2789.29±24.67 | | 1089.07±17.02 | |  |
| **L40** | 15.27 | 4.42 | |  | | 407.50±16.03 | | 1427.40±25.25 | | 1858.73±22.93 | |  |
| **L41** | 11.93 | 4.72 | |  | | 379.87±18.82 | | 888.97±23.32 | | 437.1±12.21 | |  |
| **L55** | 43.18 | 6.48 | |  | | 259.00 ±13.12 | | 258.37 ±11.5 | | 577.47 ±16.15 | |  |
| **L59** | 42.38 | 5.97 | |  | | 325.87 ±14.23 | | 246.43 ±19.91 | | 826.33 ±25.12 | |  |
| **L74** | 39.00 | 5.72 | |  | | 183.17±11.92 | | 394.27±11.23 | | 498.73±19.25 | |  |
| **L90** | 12.26 | 4.91 | |  | | 134.88±15.95 | | 278.69±10.01 | | 128.18±16.38 | |  |
| **L92** | 57.45 | 5.31 | |  | | 86.37 ±11.82 | | 218.37 ±17.35 | | 398.93 ±14.05 | |  |
| **L101** | 12.26 | | 4.38 | |  | | 3661.63±45.36 | | 9343.17±52.96 | | 10776.47±59.79 | |
| **L102** | 38.04 | | 4.89 | |  | | 77.57±11.35 | | 230.60±18.09 | | 253.07±19.69 | |
| **L103** | 65.50 | | 4.90 | |  | | 99.17±13.58 | | 273.53±21.02 | | 397.40±25.05 | |
| **up regulated >5 times** | | | | | | | | | | | |  |
| **L5** | 51.67 | 5.11 | |  | | 726.45±17.94 | | 5017.17±34.79 | | 1403.83 ±23.21 | |  |
| **L6** | 44.79 | 4.60 | |  | | 101.97±13.32 | | 794.70±11.02 | | 241.00±19.24 | |  |
| **L25** | 29.97 | 4.63 | |  | | 135.10±11.68 | | 986.53±10.43 | | 382.37±16.89 | |  |
| **L104** | 20.73 | | 5.00 | |  | | 108.53±10.68 | | 569.63±19.49 | | 314.37±18.88 | |
| **L105** | 56.33 | | 5.79 | |  | | 262.20±15.23 | | 1333.70±31.21 | | 704.57±26.78 | |
| **down-regulated 1.5-2 times** | | | | | | | | | | | |  |
| **L2** | 102.46 | 4.85 | |  | | 398.50±17.76 | | 242.67±17.37 | | 336.47±16.85 | |  |
| **L47** | 74.39 | 5.56 | |  | | 2442.60±22.32 | | 1598.63±22.25 | | 1462.50±22.75 | |  |
| **L48** | 74.85 | 5.29 | |  | | 685.43±15.37 | | 364.27±14.17 | | 276.30±12.17 | |  |
| **L50** | 57.86 | 5.90 | |  | | 692.33±22.17 | | 354.41±15.15 | | 298.29±13.42 | |  |
| **L51** | 56.08 | 5.88 | |  | | 421.73±16.12 | | 226.00±13.32 | | 667.83±17.53 | |  |
| **L52** | 53.79 | 6.49 | |  | | 498.30 ±15.12 | | 263.53 ±16.43 | | 215.33 ±15.12 | |  |
| **L53** | 48.50 | 5.51 | |  | | 1526.93 ±26.18 | | 797.90 ±18.67 | | 813.20 ±20.98 | |  |
| **L57** | 45.13 | 5.47 | |  | | 453.07±16.21 | | 272.80±15.14 | | 397.77±15.43 | |  |
| **L58** | 42.69 | 5.91 | |  | | 3440.63±25.37 | | 2082.77±25.33 | | 4210.10±22.12 | |  |
| **L61** | 40.94 | 5.49 | |  | | 13767.69±22.15 | | 8143.55±20.12 | | 8013.58±25.38 | |  |
| **L64** | 39.87 | 5.80 | |  | | 499.93 ±17.25 | | 291.87±16.72 | | 868.43 ±19.25 | |  |
| **L65** | 39.20 | 6.22 | |  | | 1846.16±20.19 | | 1122.12±18.16 | | 1936.42±20.17 | |  |
| **L66** | 37.79 | 6.69 | |  | | 3072.03 ±30.32 | | 1677.97 ±28.43 | | 1926.67 ±30.12 | |  |
| **L67** | 37.29 | 6.57 | |  | | 454.60 ±19.52 | | 234.53±10.24 | | 381.24±15.65 | |  |
| **L69** | 29.43 | 5.95 | |  | | 563.97±16.27 | | 310.00 ±17.18 | | 307.63 ±14.98 | |  |
| **L73** | 15.71 | 6.03 | |  | | 590.47±16.12 | | 385.30 ±14.15 | | 584.40 ±15.14 | |  |
| **L78** | 37.37 | 5.20 | |  | | 591.45±14.36 | | 292.07 ±12.98 | | 373.27 ±10.72 | |  |
| **Spot ID^※^** | **Experimental** | | |  | | **Spots intensity** | | | | | |  |
|  | **Mr(kDa)** | **pI** | |  | | **0 h** | | **24 h** | | **48 h** | |  |
| **L93** | 45.33 | 5.24 | |  | | 353.90±17.56 | | 195.52±14.38 | | 371.60±12.87 | |  |
| **L106** | 51.97 | | 6.58 | |  | | 2558.33±42.32 | | 1445.27±31.25 | | 1581.63±37.49 | |
| **L107** | 41.80 | | 6.03 | |  | | 577.90±23.16 | | 341.67±20.58 | | 343.07±26.66 | |
| **down-regulated 2-5 times** | | | | | | | | | | | |  |
| **L45** | 112.12 | 6.31 | |  | | 2158.73 ±26.23 | | 864.67 ±18.32 | | 981.53 ±19.24 | |  |
| **L46** | 75.97 | 5.49 | |  | | 2365.68 ±24.18 | | 1173.46 ±18.68 | | 967.72 ±19.42 | |  |
| **L68** | 34.75 | 6.57 | |  | | 3534.43 ±34.16 | | 1094.03±24.15 | | 2306.83 ±34.13 | |  |
| **L72** | 22.73 | 6.05 | |  | | 548.37±19.23 | | 246.83 ±4.23 | | 657.50 ±18.91 | |  |
| **L108** | 37.17 | | 6.62 | |  | | 1207.63±25.30 | | 249.00±18.47 | | 377.33±20.09 | |
| **L109** | 127.25 | | 5.44 | |  | | 815.33±28.51 | | 185.80±19.15 | | 136.20±18.18 | |
| **L110** | 32.99 | | 6.44 | |  | | 1109.30±31.06 | | 339.77±20.14 | | 490.40±23.48 | |
| **L111** | 52.44 | | 6.11 | |  | | 2262.33±39.89 | | 869.00±29.09 | | 1101.60±31.49 | |
| **down-regulated >5 times** | | | | | | | | | | | |  |
| **L60** | 42.60 | 6.61 | |  | | 2047.23 ±20.23 | | 303.30 ±17.34 | | 1178.55 ±18.35 | |  |
| **L112** | 47.92 | | 6.66 | |  | | 9013.20±59.38 | | 435.43±21.04 | | 1615.85±37.35 | |
| **Induced or lost spots** | | | | | | | | | | | |  |
| **L3** | 68.33 | 5.69 | |  | | 0.00 | | 312.97 ±14.15 | | 91.87±13.32 | |  |
| **L44** | 51.84 | 4.90 | |  | | 344.47 ±13.15 | | 0.00 | | 840.67 ±18.53 | |  |
| **L54** | 47.70 | 5.73 | |  | | 175.27 ±13.87 | | 0.00 | | 0.00 | |  |
| **L75** | 21.10 | 5.92 | |  | | 0.00 | | 108.50 ±11.89 | | 1741.63±14.53 | |  |
| **L76** | 60.31 | 4.53 | |  | | 0.00 | | 197.90 ±12.01 | | 221.07 ±12.25 | |  |
| **L77** | 38.63 | 4.53 | |  | | 0.00 | | 278.53 ±13.01 | | 205.80 ±12.05 | |  |
| **L79** | 36.67 | 6.83 | |  | | 0.00 | | 379.83±15.98 | | 258.7 ±10.33 | |  |
| **L80** | 34.39 | 6.87 | |  | | 0.00 | | 1115.73 ±23.31 | | 221.07 ±12.98 | |  |
| **L82** | 35.12 | 6.31 | |  | | 0.00 | | 716.33 ±17.23 | | 62.53 ±10.82 | |  |
| **L83** | 32.90 | 5.52 | |  | | 0.00 | | 642.83±18.95 | | 106.57 ±11.03 | |  |
| **L85** | 29.68 | 6.75 | |  | | 118.97±16.97 | | 256.57±18.69 | | 0.00 | |  |
| **L86** | 18.64 | 4.93 | |  | | 0.00 | | 610.20 ±16.45 | | 377.30 ±13.84 | |  |
| **L87** | 12.79 | 5.02 | |  | | 0.00 | | 579.43 ±15.42 | | 89.13 ±10.84 | |  |
| **L88** | 13.77 | 5.10 | |  | | 0.00 | | 770.47 ±17.95 | | 360.63 ±15.32 | |  |
| **L89** | 13.38 | 5.32 | |  | | 0.00 | | 882.70 ±18.43 | | 1123.13 ±13.98 | |  |
| **L91** | 54.92 | 5.76 | |  | | 0.00 | | 88.50 ±17.53 | | 328.97 ±13.07 | |  |
| **L95** | 35.94 | 6.47 | |  | | 0.00 | | 95.57 ±10.76 | | 532.00 ±15.97 | |  |
| **L97** | 22.55 | 4.82 | |  | | 0.00 | | 103.20 ±14.87 | | 73.93 ±12.55 | |  |
| **L98** | 37.46 | 5.74 | |  | | 0.00 | | 88.50 ±10.67 | | 466.97 ±14.92 | |  |
| **L113** | 97.97 | | 6.72 | |  | | 0.00 | | 41.70±8.91 | | 519.90±21.08 | |
| **L114** | 57.8 | | 6.78 | |  | | 177.13±18.16 | | 0.00 | | 0.00 | |
| **L115** | 69.12 | | 6.65 | |  | | 250.47±19.25 | | 0.00 | | 0.00 | |

**^※^** All the protein spot IDs listed is the DEPs in the 2-DE maps of leaf proteome (shown in Figure S1 A, B, C); The protein spots marked with red color indicate the DEPs up-regulated at 24 h of drought-treatment but down-regulated at 48 h; while those with green color down-regulated at 24 h but up-regulated at 48 h.
