# Supplementary material for: Physiological and Comparative Proteomic Analysis Reveals Different Drought Responses in Roots and Leaves of Drought-Tolerant Wild Wheat (Triticum boeoticum)
Source: PLoS One. 2015 Apr 10;10(4):e0121852. doi: 10.1371/journal.pone.0121852 (PMC4393031; doi:10.1371/journal.pone.0121852)
Supplement: S3 Table — (DOC) [file pone.0121852.s006.doc]

**S3 Table.** The intensity of differentially changed protein spots (DEPs) in the roots of *Triticum boeoticum* plants at different time point of drought-treatment.

| **Spot ID^※^** | **Experimental** | |  | **Spots intensity** | | |
| --- | --- | --- | --- | --- | --- | --- |
|  | **Mr(kDa)** | **pI** |  | **0 h** | **24 h** | **48 h** |
| **up regulated 1.5-2 times** | | | | | | |
| **R22** | 71.62 | 5.61 |  | 85.27±17.87 | 150.30±18.67 | 134.50±18.24 |
| **R37** | 46.94 | 5.27 |  | 239.83±12.12 | 438.50±19.34 | 365.67±11.32 |
| **R81** | 40.91 | 5.01 |  | 1276.90±21.67 | 2338.20±29.25 | 2062.20±30.89 |
| **R85** | 40.06 | 6.61 |  | 326.57±16.23 | 544.30±16.63 | 565.87±15.64 |
| **R14** | 47.04 | 6.27 |  | 179.63±16.47 | 211.80±18.53 | 17.83±4.42 |
| **R86** | 44.71 | 5.70 |  | 736.87±25.37 | 1168.20±38.42 | 1389.00±36.29 |
| **R87** | 52.09 | 5.65 |  | 2129.40±41.26 | 3389.40±46.25 | 3468.77±45.53 |
| **up regulated 2-5 times** | | | | | | |
| **R7** | 54.35 | 5.69 |  | 329.10±16.24 | 703.53±13.42 | 513.43±12.45 |
| **R11** | 27.49 | 5.46 |  | 96.7±9.09 | 254.3±17.02 | 196.9±17.54 |
| **R44** | 29.59 | 5.00 |  | 202.67±15.28 | 1047.90±27.69 | 557.20±18.41 |
| **R50** | 32.19 | 4.70 |  | 80.12±6.89 | 380.77±13.19 | 521.50±14.80 |
| **R65** | 50.83 | 5.74 |  | 186.40±11.85 | 930.40±22.33 | 692.60±11.49 |
| **R66** | 25.50 | 6.04 |  | 296.17±11.49 | 679.60±12.32 | 520.37±21.25 |
| **R67** | 33.49 | 6.12 |  | 143.80±17.04 | 396.23±22.43 | 283.60±15.79 |
| **R68** | 54.65 | 5.96 |  | 126.37±18.13 | 286.70±16.46 | 0.00 |
| **R69** | 61.24 | 6.25 |  | 129.13±13.22 | 392.80±21.13 | 199.30±16.75 |
| **R70** | 22.98 | 6.43 |  | 86.30±13.48 | 333.70±14.57 | 366.80±11.45 |
| **R71** | 22.67 | 6.46 |  | 22.20±3.64 | 72.70±5.65 | 171.50±10.52 |
| **R72** | 27.07 | 6.40 |  | 225.00±13.33 | 538.27±14.76 | 566.60±10.05 |
| **R73** | 30.28 | 6.48 |  | 77.70±10.92 | 355.90±19.87 | 0.00 |
| **R74** | 47.50 | 6.54 |  | 41.27±10.76 | 176.50±11.32 | 237.47±16.75 |
| **R75** | 29.25 | 6.76 |  | 202.80±10.86 | 666.00±19.53 | 0.00 |
| **R83** | 25.64 | 6.51 |  | 151.50±15.24 | 734.30±18.96 | 405.57±10.46 |
| **R82** | 49.76 | 6.15 |  | 230.10±13.12 | 165.10±12.31 | 528.93±16.79 |
| **R84** | 36.03 | 6.45 |  | 107.50±15.21 | 214.47±17.85 | 267.70±19.24 |
| **R88** | 40.57 | 5.23 |  | 27.90±4.12 | 127.67±17.21 | 131.43±14.58 |
| **R89** | 39.51 | 5.97 |  | 89.23±11.24 | 189.50±19.08 | 233.23±21.02 |
| **R90** | 36.36 | 6.77 |  | 80.80±9.38 | 302.90±21.36 | 263.40±22.47 |
| **up regulated ＞5 times** | | | | | | |
| **R76** | 43.93 | 5.45 |  | 372.23±15.55 | 2092.40±34.23 | 777.47±13.86 |
| **R77** | 25.82 | 5.87 |  | 43.83±15.64 | 227.70±18.35 | 185.93±17.21 |
| **R78** | 17.40 | 5.34 |  | 17.20±10.77 | 175.80±17.75 | 69.37±15.90 |
| **R79** | 17.47 | 5.97 |  | 11.80±10.25 | 507.10±14.43 | 474.10±16.68 |
| **R80** | 23.08 | 6.13 |  | 66.73±13.43 | 871.70±15.92 | 467.60±10.79 |
| **R91** | 26.70 | 6.61 |  | 26.90±3.58 | 359.03±19.86 | 530.83±23.78 |
| **R92** | 26.30 | 6.64 |  | 20.30±4.21 | 144.03±17.24 | 172.10±19.37 |
| **down-regulated 1.5-2 times** | | | | | | |
| **R1** | 25.85 | 5.20 |  | 778.90±12.12 | 405.70±12.56 | 291.03±14.67 |
| **Spot ID^※^** | **Experimental** | |  | **Spots intensity** | | |
|  | **Mr(kDa)** | **pI** |  | **0 h** | **24 h** | **48 h** |
| **R4** | 49.73 | 5.53 |  | 432.73±15.23 | 248.00±10.03 | 150.13±13.25 |
| **R40** | 57.26 | 5.86 |  | 667.33±15.77 | 380.53±15.26 | 401.77±12.89 |
| **R43** | 35.73 | 6.82 |  | 478.87±15.32 | 290.30±10.23 | 216.30±25.89 |
| **R45** | 25.90 | 5.53 |  | 985.90±14.56 | 1213.40±32.18 | 532.17±14.25 |
| **R93** | 41.34 | 5.25 |  | 140.30±12.46 | 83.60±10.35 | 82.47±9.78 |
| **R94** | 33.57 | 5.42 |  | 363.27±19.23 | 221.07±18.65 | 234.30±18.73 |
| **R95** | 34.65 | 5.69 |  | 722.00±31.22 | 464.90±21.34 | 397.70±20.25 |
| **down-regulated 2-5 times** | | | | | | |
| **R2** | 27.49 | 5.30 |  | 399.20±13.32 | 373.30±14.42 | 108.40±15.05 |
| **R5** | 54.00 | 5.40 |  | 550.10±17.98 | 176.40±15.23 | 134.57±14.12 |
| **R6** | 51.12 | 5.56 |  | 215.00±12.88 | 216.40±19.12 | 84.13±10.45 |
| **R8** | 49.88 | 5.67 |  | 532.50±15.25 | 453.67±14.42 | 208.07±11.12 |
| **R10** | 58.36 | 6.06 |  | 693.30±18.75 | 292.00±15.43 | 195.93±11.45 |
| **R12** | 56.17 | 5.78 |  | 416.30±15.43 | 107.33±15.32 | 42.87±12.98 |
| **R13** | 63.74 | 5.63 |  | 409.70±18.79 | 131.20±11.87 | 24.00±11.92 |
| **R15** | 33.31 | 5.32 |  | 688.60±12.25 | 295.27±17.64 | 118.70±16.34 |
| **R16** | 32.43 | 5.42 |  | 560.23±16.98 | 221.70±15.23 | 247.80±16.42 |
| **R17** | 43.57 | 5.17 |  | 583.13±18.79 | 192.90±14.98 | 327.93±19.34 |
| **R21** | 48.10 | 5.40 |  | 316.70±18.56 | 87.90±14.67 | 61.33±13.23 |
| **R23** | 41.59 | 5.72 |  | 1608.50±28.12 | 428.30±18.23 | 1032.50±30.12 |
| **R24** | 48.55 | 5.85 |  | 426.23±16.45 | 282.27±14.76 | 145.97±15.54 |
| **R25** | 54.52 | 5.73 |  | 334.40±16.78 | 94.83±10.75 | 188.33±15.34 |
| **R26** | 61.69 | 5.74 |  | 1410.70±32.21 | 521.93±17.56 | 424.40±14.56 |
| **R27** | 59.92 | 5.78 |  | 587.40±12.32 | 143.97±12.78 | 126.43±18.45 |
| **R28** | 57.28 | 5.81 |  | 547.80±16.78 | 138.90±17.56 | 168.37±19.67 |
| **R29** | 59.24 | 5.88 |  | 1132.40±27.97 | 243.43±16.25 | 516.43±15.78 |
| **R30** | 58.20 | 5.75 |  | 457.50±17.89 | 143.70±18.87 | 209.90±12.98 |
| **R31** | 55.09 | 6.11 |  | 481.27±17.85 | 177.77±15.34 | 276.00±14.32 |
| **R32** | 44.37 | 6.15 |  | 703.90±18.35 | 234.10±10.21 | 306.00±17.89 |
| **R33** | 61.80 | 5.22 |  | 1070.93±25.55 | 526.03±15.76 | 471.95±16.76 |
| **R34** | 65.50 | 6.13 |  | 1193.40±27.67 | 510.47±12.65 | 175.93±11.24 |
| **R41** | 48.79 | 6.35 |  | 628.60±13.89 | 263.57±17.87 | 210.38±12.42 |
| **R46** | 59.83 | 5.60 |  | 1389.20±34.53 | 489.30±18.31 | 757.40±19.25 |
| **R96** | 38.28 | 5.64 |  | 313.00±18.58 | 130.10±11.09 | 89.70±10.23 |
| **R97** | 53.06 | 5.91 |  | 1074.17±25.24 | 496.00±21.07 | 383.10±18.93 |
| **down-regulated >5 times** | | | | | | |
| **R35** | 42.14 | 6.82 |  | 3160.90±48.77 | 624.90±16.79 | 1255.70±24.52 |
| **R36** | 52.06 | 5.20 |  | 446.90±12.76 | 86.03±14.45 | 195.00±12.12 |
| **R38** | 65.20 | 5.22 |  | 1358.23±26.25 | 254.40±18.64 | 38.40±11.67 |
| **R39** | 48.26 | 5.67 |  | 629.10±18.82 | 94.90±14.67 | 77.60±13.12 |
| **R42** | 40.26 | 5.02 |  | 508.57±14.98 | 25.40±2.29 | 37.30±3.76 |
| **R64** | 17.71 | 6.85 |  | 993.70±20.43 | 115.07±13.14 | 110.23±12.68 |
| **R98** | 31.11 | 5.26 |  | 433.67±25.37 | 53.90±9.64 | 67.80±8.27 |
| **Spot ID^※^** | **Experimental** | |  | **Spots intensity** | | |
|  | **Mr(kDa)** | **pI** |  | **0 h** | **24 h** | **48 h** |
| **R99** | 40.26 | 5.02 |  | 508.57±21.76 | 25.40±7.24 | 37.30±8.31 |
| **Induced or lost spots** | | | | | | |
| **R3** | 53.31 | 5.27 |  | 642.33±19.02 | 0.00 | 239.20±12.21 |
| **R9** | 86.54 | 5.67 |  | 289.30±19.82 | 0.00 | 60.17±10.24 |
| **R18** | 65.81 | 5.27 |  | 1286.63±26.75 | 266.97±12.47 | 0.00 |
| **R19** | 72.58 | 5.29 |  | 737.67±19.54 | 168.10±15.98 | 0.00 |
| **R20** | 71.70 | 5.34 |  | 561.53±15.76 | 145.87±14.23 | 0.00 |
| **R47** | 61.59 | 6.13 |  | 0.00 | 221.30±10.23 | 115.70±15.98 |
| **R48** | 36.28 | 6.58 |  | 0.00 | 105.20±12.67 | 172.20±17.18 |
| **R49** | 31.09 | 6.55 |  | 0.00 | 123.23±18.16 | 288.93±15.19 |
| **R51** | 60.40 | 6.18 |  | 0.00 | 98.40±10.13 | 49.70±10.45 |
| **R52** | 24.93 | 5.91 |  | 0.00 | 350.90±18.12 | 173.33±19.24 |
| **R53** | 24.10 | 6.16 |  | 0.00 | 864.40±12.98 | 754.70±14.25 |
| **R54** | 25.36 | 6.19 |  | 0.00 | 148.40±13.12 | 129.32±13.23 |
| **R55** | 16.94 | 5.04 |  | 0.00 | 151.30±16.35 | 131.02±14.54 |
| **R56** | 17.83 | 5.78 |  | 0.00 | 558.40±18.12 | 223.80±19.15 |
| **R57** | 18.51 | 6.20 |  | 0.00 | 394.00±18.23 | 199.60±16.76 |
| **R58** | 17.26 | 6.35 |  | 0.00 | 176.30±12.12 | 72.30±10.17 |
| **R59** | 64.93 | 6.59 |  | 0.00 | 259.90±12.45 | 189.00±13.12 |
| **R60** | 64.72 | 6.51 |  | 0.00 | 93.00±10.56 | 64.40±10.65 |
| **R61** | 40.68 | 6.57 |  | 0.00 | 76.20±10.76 | 273.07±17.97 |
| **R62** | 120.73 | 6.09 |  | 95.90±10.13 | 0.00 | 0.00 |
| **R63** | 81.85 | 5.69 |  | 230.80±17.78 | 0.00 | 0.00 |
| **R100** | 25.47 | 4.61 |  | 0.00 | 246.40±15.24 | 105.60±11.09 |
| **R101** | 43.94 | 5.92 |  | 0.00 | 108.43±10.98 | 20.97±4.23 |
| **R102** | 25.96 | 4.11 |  | 411.90±25.38 | 0.00 | 0.00 |

**^※^** All theprotein spot IDs listed is the DEPs in the 2-DE maps of root proteome (shown in Figure S1 D, E, F); The protein spots marked with red color indicate the DEPs up-regulated at 24 h of drought-treatment but down-regulated at 48 h.
