# Supplementary material for: Physiological and Comparative Proteomic Analysis Reveals Different Drought Responses in Roots and Leaves of Drought-Tolerant Wild Wheat (Triticum boeoticum)
Source: PLoS One. 2015 Apr 10;10(4):e0121852. doi: 10.1371/journal.pone.0121852 (PMC4393031; doi:10.1371/journal.pone.0121852)
Supplement: S4 Table — (DOC) [file pone.0121852.s007.doc]

**S4 Table.** Differentially changed proteins (DEPs) in the leaves of wild wheat (*T. boeoticum*) plants under drought-treatment identified by MALDI-TOF-TOF

| **Spot ID** | **Protein annotation** | **gi in NCBI** | **Species** | **Ps^a^** | **Ps**  **C. I. %^b^** | **Exp/The^c^** | |  | **Fold-change^#^** | | **L^d^** | **M^e^** | **C^f^ (%)** |
| --- | --- | --- | --- | --- | --- | --- | --- | --- | --- | --- | --- | --- | --- |
|  |  |  |  |  |  | Mr (kDa) | *p*I |  | 24 h | 48 h |  |  |  |
|  |  |  |  |  |  |  |  |  |  |  |  |  |  |
| **UP-regulated** | | | | | | | | | | | | | |
| **Photosynthesis** | | | | | | | | | | | | | |
| **L4** | ribulose-1,5-bisphosphate carboxylase/oxygenase large subunit | gi|144583566 | Crithopsis delileana | 848 | 100.00 | 51.80/52.44 | 5.03/6.04 |  | 2.94 | -1.31 | chl | 24 | 55 |
| **L5** | ribulose-1,5-bisphosphate carboxylase/oxygenase large subunit | gi|144583566 | Crithopsis delileana | 983 | 100.00 | 51.67/52.44 | 5.11/6.04 |  | 6.91 | 1.93 | chl | 33 | 67 |
| **L9** | ribulosebiphosphate carboxylase | gi|1488575 | Leymus erianthus | 680 | 100.00 | 50.12/53.36 | 5.53/5.95 |  | 4.70 | -1.22 | chl | 20 | 48 |
| **L10** | ribulose-1,5-bisphosphate carboxylase/oxygenase large subunit | gi|144583566 | Crithopsis delileana | 1080 | 100.00 | 49.67/52.44 | 5.66/6.04 |  | 3.18 | 1.02 | chl | 27 | 56 |
| **L14** | Phosphoribulokinase | gi|125580 | Triticum aestivum | 506 | 100.00 | 40.04/45.51 | 5.11/5.72 |  | 1.60 | -1.22 | chl | 15 | 49 |
| **L19** | Fructose-1,6-bisphosphate aldolase | [gi|326527757](http://www.matrixscience.com/cgi/protein_view.pl?file=../data/20091222/FtmmfaEEe.dat&hit=1) | Hordeum vulgare subsp. Vulgare | 441 | 100.00 | 35.08/42.16 | 5.29/6.78 |  | 2.20 | -1.14 | chl | 14 | 50 |
| **L26** | ribose 5-phosphate isomerase | gi|326502358 | Hordeum vulgare subsp. Vulgare | 101 | 99.99 | 28.38/28.79 | 4.73/6.00 |  | 2.18 | 1.97 | chl | 5 | 23 |
| **Spot ID** | **Protein annotation** | **gi in NCBI** | **Species** | **Ps^a^** | **Ps**  **C. I. %^b^** | **Exp/The^c^** | |  | **Fold-change^#^** | | **L^d^** | **M^e^** | **C^f^ (%)** |
|  |  |  |  |  |  | Mr (kDa) | *p*I |  | 24 h | 48 h |  |  |  |
| **L27** | ribose 5-phosphate isomerase | [gi|326502358](http://www.matrixscience.com/cgi/protein_view.pl?file=../data/20091222/FtmmfaHnT.dat&hit=1) | Hordeum vulgare subsp. Vulgare | 249 | 100.00 | 28.15/28.79 | 4.81/6.00 |  | 2.76 | 1.57 | chl | 3 | 15 |
| **L28** | 2-polyprenylphenol hydroxylase | [gi|326530368](http://www.matrixscience.com/cgi/protein_view.pl?file=../data/20091222/FtmmfaHaL.dat&hit=1) | Hordeum vulgare subsp. Vulgare | 556 | 100.00 | 25.62/30.17 | 4.93/6.75 |  | 2.09 | 1.62 | chl | 9 | 55 |
| **L39** | PREDICTED: thylakoid lumenal 16.5 kDa protein | [gi|357123664](http://www.matrixscience.com/cgi/protein_view.pl?file=../data/20091222/FtmmfaYae.dat&hit=2) | Brachypodium distachyon | 146 | 100.00 | 14.77/24.79 | 5.01/8.70 |  | 1.83 | 1.58 | chl | 1 | 8 |
| **L41** | PREDICTED: thylakoid membrane phosphoprotein 14 kDa | [gi|357147204](http://www.matrixscience.com/cgi/protein_view.pl?file=../data/20091222/FtmmfaYwE.dat&hit=1) | Brachypodium distachyon | 98 | 99.98 | 11.93/17.80 | 4.72/8.76 |  | 2.34 | 1.15 | chl | 5 | 44 |
| **L79** | Glyceraldehyde 3-phosphate dehydrogenase | gi|326500100 | Hordeum vulgare subsp. Vulgare | 207 | 100.00 | 36.67/43.02 | 6.83/7.60 |  | + | + | chl | 12 | 29 |
| **L80** | ferredoxin-NADP(H) oxidoreductase | gi|20302471 | Triticum aestivum | 580 | 100.00 | 34.39/39.18 | 6.87/8.29 |  | + | + | chl | 18 | 54 |
| **L84** | putative carbonic anhydrase | gi|290875537 | Triticum turgidum subsp.durum x Secale cereale | 113 | 100.00 | 31.70/28.51 | 6.59/8.35 |  | 1.86 | 2.61 | chl | 10 | 45 |
| **L96** | photosystem II oxygen-evolving enhancer protein 1 | gi|326487308 | Hordeum vulgare subsp. vulgare | 162 | 100.00 | 32.86/34.68 | 5.05/5.75 |  | 1.91 | 1.52 | chl | 13 | 50 |
| **L98** | Coproporphyrinogen III oxidase | gi|14030667 | Arabidopsis thaliana | 274 | 100.00 | 37.46/34.44 | 5.74/5.60 |  | + | + | chl | 9 | 26 |
| **Detoxification and defense** | | | | | | | | | | | | | |
| **L23** | Heme-dependent peroxidases | gi|326513514 | Hordeum vulgare subsp. Vulgare | 79 | 98.76 | 27.09/37.71 | 5.80/7.57 |  | 1.90 | -1.10 | chl | 1 | 4 |
| **Spot ID** | **Protein annotation** | **gi in NCBI** | **Species** | **Ps^a^** | **Ps**  **C. I. %^b^** | **Exp/The^c^** | |  | **Fold-change^#^** | | **L^d^** | **M^e^** | **C^f^ (%)** |
|  |  |  |  |  |  | Mr (kDa) | *p*I |  | 24 h | 48 h |  |  |  |
| **L25** | Glutathione S-transferase | [gi|326500472](http://www.matrixscience.com/cgi/protein_view.pl?file=../data/20091222/FtmmfaHtS.dat&hit=5) | Hordeum vulgare subsp. Vulgare | 90 | 99.88 | 29.97/23.38 | 4.63/4.50 |  | 7.30 | 2.83 | nuc | 2 | 11 |
|  |  |  |  |  |  |  |  |  |  |  |  |  |  |
| **L30** | glutathione transferase F5 | [gi|23504745](http://www.matrixscience.com/cgi/protein_view.pl?file=../data/20091222/FtmmfaSTL.dat&hit=1) | Triticum aestivum | 271 | 100.00 | 23.43/23.42 | 6.54/5.78 |  | 1.67 | 2.02 | cyt | 5 | 36 |
| **L31** | Peroxiredoxin | [gi|326496957](http://www.matrixscience.com/cgi/protein_view.pl?file=../data/20091222/FtmmfaHOE.dat&hit=1) | Hordeum vulgare subsp. Vulgare | 907 | 100.00 | 22.43/28.44 | 4.92/6.33 |  | 1.81 | 1.52 | cyt | 12 | 50 |
| **L32** | Peroxiredoxin | [gi|326496957](http://www.matrixscience.com/cgi/protein_view.pl?file=../data/20091222/FtmmfaHTh.dat&hit=1) | Hordeum vulgare subsp. vulgare | 639 | 100.00 | 21.32/28.44 | 4.76/6.33 |  | 3.59 | 6.03 | cyt | 11 | 47 |
| **L34** | Peroxiredoxin | [gi|38344034](http://www.matrixscience.com/cgi/protein_view.pl?file=../data/20091222/FtmmfaSeT.dat&hit=1) | Oryza sativa Japonica Group | 75 | 96.59 | 21.55/18.27 | 4.92/4.67 |  | 3.93 | 2.08 | cyt | 5 | 25 |
| **L38** | Cu/Zn superoxide dismutase | [gi|1568639](http://www.matrixscience.com/cgi/protein_view.pl?file=../data/20091222/FtmmfaSST.dat&hit=2) | Triticum aestivum | 211 | 100.00 | 16.87/20.42 | 5.28/5.35 |  | 1.99 | 1.65 | chl | 5 | 46 |
| **L43** | ADP-heptose:LPS heptosyltransferase II | [gi|326487296](http://www.matrixscience.com/cgi/protein_view.pl?file=../data/20091222/FtmmfaYTe.dat&hit=1) | Hordeum vulgare subsp. Vulgare | 187 | 100.00 | 47.70/52.04 | 5.73/6.00 |  | 1.71 | 1.62 | mit | 14 | 27 |
| **L59** | PREDICTED: GDP-mannose 3,5-epimerase 2-like | gi|357156300 | Brachypodium distachyon | 186 | 100.00 | 42.38/42.45 | 5.97/5.84 |  | -1.32 | 2.54 | cyt | 11 | 40 |
| **L70** | glutathione-S-transferase 19E50 | [gi|22022400](http://www.matrixscience.com/cgi/protein_view.pl?file=../data/20091222/FtmmfaYnt.dat&hit=1) | Triticum aestivum | 179 | 100.00 | 26.05/25.01 | 6.63/5.99 |  | -1.03 | 1.53 | cyt | 7 | 36 |
| **L74** | Flavone O-methyltransferase 1 | gi|75147302 | Triticum aestivum | 152 | 100.00 | 39.00/39.18 | 5.72/5.75 |  | 2.15 | 2.72 | cyt | 7 | 30 |
| **L83** | PREDICTED: lactoylglutathione lyase-like | gi|357144699 | Brachypodium distachyon | 109 | 100.00 | 32.90/32.84 | 5.52/5.34 |  | + | + | cyt | 9 | 41 |
| **L87** | thioredoxin H | gi|16517113 | Triticum aestivum | 156 | 100.00 | 12.79/13.86 | 5.02/5.12 |  | + | + | cyt | 4 | 30 |
|  |  |  |  |  |  |  |  |  |  |  |  |  |  |
| **Spot ID** | **Protein annotation** | **gi in NCBI** | **Species** | **Ps^a^** | **Ps**  **C. I. %^b^** | **Exp/The^c^** | |  | **Fold-change^#^** | | **L^d^** | **M^e^** | **C^f^ (%)** |
|  |  |  |  |  |  | Mr (kDa) | *p*I |  | 24 h | 48 h |  |  |  |
| **L97** | Peroxiredoxin | gi|326496957 | Hordeum vulgare subsp. vulgare | 207 | 100.00 | 22.55/28.44 | 4.82/6.33 |  | + | + | chl | 10 | 54 |
| **Proteins metabolism** | | | | | | | | | | | | | |
| **L1** | Von Willebrand factor type A | gi|326505132 | Hordeum vulgare subsp. vulgare | 238 | 100.00 | 62.71/55.57 | 4.83/4.71 |  | 2.19 | 3.39 | mit | 13 | 27 |
| **L6** | triticain alpha | gi|111073715 | Triticum aestivum | 213 | 100.00 | 44.79/51.57 | 4.60/5.01 |  | 7.79 | 2.36 | vac | 4 | 17 |
| **L71** | proteasome_alpha_type_6 | gi|326495678 | Hordeum vulgare subsp. vulgare | 105 | 100.00 | 26.03/27.55 | 6.70/6.33 |  | 1.72 | 2.14 | cyt | 7 | 34 |
| **L76** | 26S proteasome subunit | gi|326510107 | Hordeum vulgare subsp. Vulgare | 77 | 98.04 | 60.31/42.15 | 4.53/4.40 |  | + | + | nuc | 2 | 7 |
| **L89** | cysteine proteinase inhibitor | gi|222101569 | Triticum aestivum | 124 | 100.00 | 13.38/11.33 | 5.32/5.40 |  | + | + | cyt | 4 | 47 |
| **L91** | PREDICTED: selenium-binding protein 1-like | gi|357126490 | Brachypodium distachyon | 95 | 99.99 | 54.92/53.72 | 5.76/5.59 |  | + | + | cyt | 10 | 30 |
| **Chaperones** | | | | | | | | | | | | | |
| **L8** | PREDICTED: peptidyl-prolyl cis-trans isomerase CYP38 | gi|357147646 | Brachypodium distachyon | 680 | 100.00 | 42.73/46.48 | 4.70/4.83 |  | 1.89 | 1.60 | chl | 12 | 37 |
| **L36** | peptidylprolyl isomerase | [gi|133741925](http://www.matrixscience.com/cgi/protein_view.pl?file=../data/20091222/FtmmfaSSh.dat&hit=1) | Triticum aestivum | 400 | 100.00 | 18.34/26.08 | 6.83/9.40 |  | 1.81 | 1.08 | mit | 8 | 37 |
| **L40** | Chain v protein | [gi|313103619](http://www.matrixscience.com/cgi/protein_view.pl?file=../data/20091222/FtmmfaYwR.dat&hit=2) | Triticum aestivum | 132 | 100.00 | 15.27/11.64 | 4.42/4.37 |  | 3.50 | 4.56 | nuc | 3 | 38 |
| **L77** | PREDICTED: ankyrin repeat domain-containing protein 2-like | gi|357148670 | Brachypodium distachyon | 175 | 100.00 | 38.63/36.50 | 4.53/4.52 |  | + | + | nuc | 7 | 26 |
|  |  |  |  |  |  |  |  |  |  |  |  |  |  |
| **Spot ID** | **Protein annotation** | **gi in NCBI** | **Species** | **Ps^a^** | **Ps**  **C. I. %^b^** | **Exp/The^c^** | |  | **Fold-change^#^** | | **L^d^** | **M^e^** | **C^f^ (%)** |
|  |  |  |  |  |  | Mr (kDa) | *p*I |  | 24 h | 48 h |  |  |  |
| **L86** | putative peptidyl-prolyl cis-trans isomerase | gi|13486733 | Oryza sativa Japonica Group | 107 | 100.00 | 18.64/25.27 | 4.93/8.05 |  | + | + | chl | 4 | 26 |
| **L90** | heat shock protein 70 | gi|56554972 | Medicago sativa | 323 | 100.00 | 12.26/71.35 | 4.91/5.08 |  | 2.07 | -1.05 | cyt | 18 | 33 |
| **L92** | PREDICTED: chaperonin CPN60-2 | gi|357114085 | Brachypodium distachyon | 105 | 100.00 | 57.45/61.36 | 5.31/5.52 |  | + | + | mit | 7 | 12 |
| **Transcription and translation-associated proteins** | | | | | | | | | | | | | |
| **L7** | PREDICTED: 30S ribosomal protein S1 | gi|357112501 | Brachypodium distachyon | 316 | 100.00 | 43.55/43.64 | 4.67/4.68 |  | 2.23 | 1.87 | cyt | 14 | 36 |
| **L15** | 30S ribosomal protein S5 | gi|326506396 | Hordeum vulgare subsp. Vulgare | 231 | 100.00 | 36.60/35.44 | 5.09/5.08 |  | 1.68 | 1.10 | nuc | 10 | 29 |
| **L22** | WD40 repeat protein | [gi|326491885](http://www.matrixscience.com/cgi/protein_view.pl?file=../data/20091222/FtmmfaESE.dat&hit=1) | Hordeum vulgare subsp. Vulgare | 688 | 100.00 | 32.73/36.66 | 6.28/5.97 |  | 1.81 | 1.17 | nuc | 13 | 55 |
| **L24** | putative elongation factor 1 beta | gi|7711024 | Hordeum vulgare subsp. Vulgare | 305 | 100.00 | 31.52/24.72 | 4.57/4.52 |  | 2.05 | 1.53 | nuc | 7 | 38 |
| **L37** | Ribosomal_L7_L12 | [gi|326495080](http://www.matrixscience.com/cgi/protein_view.pl?file=../data/20091222/FtmmfaSSL.dat&hit=1) | Hordeum vulgare subsp. Vulgare | 517 | 100.00 | 17.23/18.14 | 4.90/5.60 |  | 1.13 | 1.72 | nuc | 5 | 44 |
| **L75** | Ribosomal protein L10 | gi|326507838 | Hordeum vulgare subsp. Vulgare | 721 | 100.00 | 21.10/24.20 | 5.92/8.31 |  | + | + | mit | 14 | 69 |
| **L81** | nascent polypeptide-associated complex alpha subunit-like protein | gi|195639862 | Zea mays | 228 | 100.00 | 31.01/14.67 | 4.42/5.21 |  | 1.87 | 1.99 | nuc | 5 | 50 |
| **Amino acid and nitrogen metabolism** | | | | | | | | | | | | | |
| **Spot ID** | **Protein annotation** | **gi in NCBI** | **Species** | **Ps^a^** | **Ps**  **C. I. %^b^** | **Exp/The^c^** | |  | **Fold-change^#^** | | **L^d^** | **M^e^** | **C^f^ (%)** |
|  |  |  |  |  |  | Mr (kDa) | *p*I |  | 24 h | 48 h |  |  |  |
| **L12** | plastid glutamine synthetase 2 | gi|251832993 | Triticum aestivum | 365 | 100.00 | 44.14/46.99 | 5.04/5.75 |  | 2.08 | 2.20 | chl | 10 | 39 |
| **L13** | plastid glutamine synthetase 2 | gi|251832993 | Triticum aestivum | 792 | 100.00 | 43.89/46.99 | 5.13/5.75 |  | 1.63 | 1.49 | chl | 15 | 51 |
| **L20** | putative mitchondrial cysteine synthase precursor | gi|213958273 | Aegilops speltoides | 714 | 100.00 | 34.97/22.58 | 5.33/5.36 |  | 2.18 | 2.14 | mit | 11 | 72 |
| **L21** | Cysteine synthase A | [gi|585032](http://www.matrixscience.com/cgi/protein_view.pl?file=../data/20091222/FtmmfaEET.dat&hit=1) | Triticum aestivum | 120 | 100.00 | 35.59/34.21 | 5.40/5.48 |  | 1.88 | -1.04 | cyt | 7 | 33 |
| **L42** | glutamate decarboxylase, putative, expressed | [gi|300681536](http://www.matrixscience.com/cgi/protein_view.pl?file=../data/20091222/FtmmfaYOe.dat&hit=1) | Triticum aestivum | 204 | 100.00 | 50.39/54.47 | 5.72/5.55 |  | 1.68 | 1.10 | cyt | 13 | 35 |
| **L62** | S-adenosylmethionine synthase 3 | gi|122220777 | Hordeum vulgare | 719 | 100.00 | 43.60/43.14 | 5.77/5.51 |  | 1.05 | 1.53 | cyt | 19 | 65 |
| **L82** | beta-cyanoalanine synthase | gi|295917894 | Triticum aestivum | 233 | 100.00 | 35.12/40.22 | 6.31/6.35 |  | + | + | mit | 12 | 26 |
| **L95** | ornithine carbamoyltransferase | gi|326497643 | Hordeum vulgare subsp. Vulgare | 242 | 100.00 | 35.94/40.40 | 6.47/6.87 |  | + | + | chl | 7 | 26 |
| **Carbon metabolism** | | | | | | | | | | | | | |
| **L29** | Carbonic anhydrase | gi|729003 | Hordeum vulgare subsp. Vulgare | 410 | 100.00 | 23.90/35.74 | 6.16/8.93 |  | 2.66 | 1.55 | mit | 8 | 44 |
| **L49** | Succinate dehydrogenase | gi|326503994 | Hordeum vulgare subsp. vulgare | 97 | 99.98 | 59.73/68.84 | 6.02/6.08 |  | 1.51 | 1.63 | mit | 9 | 21 |
| **L56** | PREDICTED: citrate synthase 4 | gi|357139257 | Brachypodium distachyon | 199 | 100.00 | 43.72/52.85 | 6.80/6.62 |  | 1.59 | 1.08 | mit | 13 | 31 |
|  |  |  |  |  |  |  |  |  |  |  |  |  |  |
| **Spot ID** | **Protein annotation** | **gi in NCBI** | **Species** | **Ps^a^** | **Ps**  **C. I. %^b^** | **Exp/The^c^** | |  | **Fold-change^#^** | | **L^d^** | **M^e^** | **C^f^ (%)** |
|  |  |  |  |  |  | Mr (kDa) | *p*I |  | 24 h | 48 h |  |  |  |
| **L78** | Fructokinases (FRKs) | gi|326513418 | Hordeum vulgare subsp. Vulgare | 259 | 100.00 | 37.37/35.91 | 5.20/5.06 |  | -1.99 | -1.56 | mit | 13 | 37 |
| **L94** | Rossmann-fold NAD(P)(+)-binding proteins | gi|326516286 | Hordeum vulgare subsp. Vulgare | 172 | 100.00 | 45.47/33.68 | 5.72/5.80 |  | 1.93 | -1.14 | cyt | 11 | 34 |
| **Nucleotide metabolism** | | | | | | | | | | | | | |
| **L16** | Proliferating Cell nuc Antigen (PCNA) domain protein | gi|326512374 | Hordeum vulgare subsp. vulgare | 274 | 100.00 | 35.55/29.44 | 4.71/4.62 |  | 3.26 | 4.88 | nuc | 9 | 53 |
| **L17** | RNA/DNA binding protein | gi|326496905 | Hordeum vulgare subsp. Vulgare | 146 | 100.00 | 32.25/28.89 | 4.89/5.41 |  | 4.30 | -1.16 | nuc | 4 | 21 |
| **Signal transduction-associated proteins** | | | | | | | | | | | | | |
| **L35** | auxin-binding protein ABP20 precursor | [gi|195616892](http://www.matrixscience.com/cgi/protein_view.pl?file=../data/20091222/FtmmfaSEO.dat&hit=1) | Zea mays | 93 | 99.95 | 18.61/20.55 | 6.05/6.01 |  | 2.57 | 1.00 | nuc | 2 | 16 |
| **Cell wall metabolism** | | | | | | | | | | | | | |
| **L63** | reversibly glycosylated polypeptide | gi|4158232 | Triticum aestivum | 540 | 100.00 | 37.01/41.99 | 5.88/5.82 |  | 1.61 | 1.70 | GA | 24 | 70 |
| **Cell membrane development** | | | | | | | | | | | | | |
| **L18** | PREDICTED: salt stress root protein RS1-like | gi|357128825 | Brachypodium distachyon | 74 | 96.26 | 33.33/21.09 | 4.99/4.96 |  | -1.05 | 1.94 | cyt | 3 | 14 |
| **Lipid metabolism** | | | | | | | | | | | | | |
|  | | | | | | | | | | | | | |
| **Spot ID** | **Protein annotation** | **gi in NCBI** | **Species** | **Ps^a^** | **Ps**  **C. I. %^b^** | **Exp/The^c^** | |  | **Fold-change^#^** | | **L^d^** | **M^e^** | **C^f^ (%)** |
|  |  |  |  |  |  | Mr (kDa) | *p*I |  | 24 h | 48 h |  |  |  |
| **L55** | PREDICTED: 3-oxoacyl-[acyl-carrier-protein] synthase I | [gi|357124929](http://www.matrixscience.com/cgi/protein_view.pl?file=../data/20091222/FtmmfaSTO.dat&hit=1) | Brachypodium distachyon | 94 | 99.96 | 43.18/49.02 | 6.48/6.72 |  | -1.00 | 2.23 | chl | 7 | 33 |
| **Down-regulated** | | | | | | | | | | | | | |
| **Photosynthesis** | | | | | | | | | | | | | |
| **L52** | ribulose-1,5-bisphosphate carboxylase/oxygenase large subunit | [gi|144583566](http://www.matrixscience.com/cgi/protein_view.pl?file=../data/20091222/FtmmfaSmS.dat&hit=1) | Crithopsis delileana | 598 | 100.00 | 53.79/52.44 | 6.49/6.04 |  | -1.89 | -2.31 | chl | 22 | 43 |
| **L53** | ribulosebiphosphate carboxylase | [gi|1488577](http://www.matrixscience.com/cgi/protein_view.pl?file=../data/20091222/FtmmfaTaR.dat&hit=4) | Elymus glaucescens | 335 | 100.00 | 48.50/53.20 | 5.51/5.95 |  | -1.91 | -1.88 | chl | 20 | 50 |
| **L61** | chlroplast ribulose-1,5-bisphosphate carboxylase activase | gi|115392208 | Triticum aestivum | 600 | 100.00 | 40.94/40.26 | 5.49/6.52 |  | -1.69 | 1.72 | chl | 13 | 40 |
| **L66** | Fructose-1,6-bisphosphate aldolase | gi|326493652 | Hordeum vulgare subsp. Vulgare | 750 | 100.00 | 37.79/38.10 | 6.69/6.06 |  | -1.83 | -1.59 | cyt | 14 | 48 |
| **L67** | putative carbonic anhydrase | [gi|290875537](http://www.matrixscience.com/cgi/protein_view.pl?file=../data/20091222/FtmmfaEmE.dat&hit=2) | Triticum turgidum subsp durum x Secale cereale | 74 | 95.99 | 37.29/28.51 | 6.57/8.35 |  | -1.94 | -1.19 | mit | 6 | 39 |
| **L68** | Glyceraldehyde 3-phosphate dehydrogenase | [gi|326500100](http://www.matrixscience.com/cgi/protein_view.pl?file=../data/20091222/FtmmfaEOR.dat&hit=1) | Hordeum vulgare subsp. Vulgare | 617 | 100.00 | 34.75/43.02 | 6.57/7.60 |  | -3.23 | -1.53 | chl | 16 | 43 |
|  |  |  |  |  |  |  |  |  |  |  |  |  |  |
| **Spot ID** | **Protein annotation** | **gi in NCBI** | **Species** | **Ps^a^** | **Ps**  **C. I. %^b^** | **Exp/The^c^** | |  | **Fold-change^#^** | | **L^d^** | **M^e^** | **C^f^ (%)** |
|  |  |  |  |  |  | Mr (kDa) | *p*I |  | 24 h | 48 h |  |  |  |
| **L69** | ribulose-1,5-bisphosphate carboxylase/oxygenase large subunit | [gi|144583566](http://www.matrixscience.com/cgi/protein_view.pl?file=../data/20091222/FtmmfaEme.dat&hit=1) | Crithopsis delileana | 310 | 100.00 | 29.43/52.44 | 5.95/6.04 |  | -1.82 | -1.83 | chl | 15 | 36 |
| **L93** | ribulose bisphosphate carboxylase/oxygenase activase | gi|326494300 | Hordeum vulgare subsp. Vulgare | 244 | 100.00 | 45.33/47.28 | 5.24/7.59 |  | -1.81 | 1.05 | chl | 12 | 42 |
| **Amino acid and nitrogen metabolism** | | | | | | | | | | | | | |
| **L45** | glycine decarboxylase P subunit | [gi|2565305](http://www.matrixscience.com/cgi/protein_view.pl?file=../data/20091222/FtmmfaYmT.dat&hit=2) | x Tritordeum sp | 401 | 100.00 | 112.12/111.97 | 6.31/6.32 |  | -2.50 | -2.20 | mit | 26 | 39 |
| **L50** | 5,10-methylene-tetrahydrofolate reductase | [gi|115589742](http://www.matrixscience.com/cgi/protein_view.pl?file=../data/20091222/FtmmfaHmT.dat&hit=1) | Triticum monococcum | 291 | 100.00 | 57.86/65.46 | 5.90/5.86 |  | -1.95 | -2.32 | cyt | 19 | 37 |
| **L58** | S-adenosylmethionine synthase 3 | gi|122220777 | Hordeum vulgare | 443 | 100.00 | 42.69/43.14 | 5.91/5.51 |  | -1.65 | 1.22 | cyt | 16 | 51 |
| **L60** | S-adenosylmethionine synthase 1 | gi|223635282 | Triticum monococcum | 279 | 100.00 | 42.60/43.25 | 6.61/5.61 |  | -6.75 | -1.74 | cyt | 15 | 52 |
| **Carbon metabolism** | | | | | | | | | | | | | |
| **L46** | transketolase | [gi|326533372](http://www.matrixscience.com/cgi/protein_view.pl?file=../data/20091222/FtmmfaYEe.dat&hit=1) | Hordeum vulgare subsp. Vulgare | 524 | 100.00 | 75.97/74.03 | 5.49/5.45 |  | -2.02 | -2.44 | cyt | 21 | 37 |
| **L47** | transketolase | [gi|326533372](http://www.matrixscience.com/cgi/protein_view.pl?file=../data/20091222/FtmmfaYSm.dat&hit=2) | Hordeum vulgare subsp. Vulgare | 319 | 100.00 | 74.39/74.03 | 5.56/5.45 |  | -1.53 | -1.67 | cyt | 16 | 40 |
| **Detoxification and defense** | | | | | | | | | | | | | |
| **L73** | USP family protein | gi|60100214 | Triticum aestivum | 380 | 100.00 | 15.71/17.97 | 6.03/5.78 |  | -1.53 | -1.01 | cyt | 7 | 55 |
| **Spot ID** | **Protein annotation** | **gi in NCBI** | **Species** | **Ps^a^** | **Ps**  **C. I. %^b^** | **Exp/The^c^** | |  | **Fold-change^#^** | | **L^d^** | **M^e^** | **C^f^ (%)** |
|  |  |  |  |  |  | Mr (kDa) | *p*I |  | 24 h | 48 h |  |  |  |
| **Proteins metabolism** | | | | | | | | | | | | | |
| **L54** | Predicted Zn-dependent peptidases | gi|255076635 | Micromonas sp. RCC299 | 74 | 95.99 | 47.70/115.17 | 5.73/4.97 |  | - | - | mit | 17 | 24 |
| **L65** | Ricin-type beta-trefoil lectin domain-like protein | gi|326517467 | Hordeum vulgare subsp. Vulgare | 109 | 100.00 | 39.20/35.76 | 6.22/5.71 |  | -1.64 | 1.05 | nuc | 6 | 29 |
| **Transcription and translation-associated proteins** | | | | | | | | | | | | | |
| **L57** | PREDICTED: elongation factor Tu | gi|357149925 | Brachypodium distachyon | 877 | 100.00 | 45.13/50.64 | 5.47/5.88 |  | -1.66 | -1.14 | chl | 16 | 46 |
| **L72** | Retrotransposon gag protein | gi|116317857 | Oryza sativa Indica Group | 81 | 99.14 | 22.73/187.01 | 6.05/9.04 |  | -4.65 | 1.27 | mit | 23 | 13 |
| **Chaperones** | | | | | | | | | | | | | |
| **L2** | heat shock protein 90 | gi|556673 | Secale cereale | 137 | 100.00 | 102.46/88.40 | 4.85/4.90 |  | -1.64 | -1.18 | mit | 13 | 22 |
| **L48** | heat shock 70 kDa protein 5 | [gi|326495158](http://www.matrixscience.com/cgi/protein_view.pl?file=../data/20091222/FtmmfaSTR.dat&hit=1) | Hordeum vulgare subsp. Vulgare | 1020 | 100.00 | 74.85/73.30 | 5.29/5.11 |  | -1.88 | -2.48 | ER | 27 | 40 |
| **Up-regulated at 24 h but down-regulated at 48 h** | | | | | | | | | | | | | |
| **Energy metabolism** | | | | | | | | | | | | | |
| **L85** | Adenylate kinase (ADK) | gi|326517593 | Hordeum vulgare subsp. Vulgare | 122 | 100.00 | 29.68/26.64 | 6.75/6.54 |  | 2.16 | - | cyt | 7 | 35 |
| **Down-regulated at 24 h but up-regulated at 48 h** | | | | | | | | | | | | | |
| **Amino acid and nitrogen metabolism** | | | | | | | | | | | | | |
|  | | | | | | | | | | | | | |
| **Spot ID** | **Protein annotation** | **gi in NCBI** | **Species** | **Ps^a^** | **Ps**  **C. I. %^b^** | **Exp/The^c^** | |  | **Fold-change^#^** | | **L^d^** | **M^e^** | **C^f^ (%)** |
|  |  |  |  |  |  | Mr (kDa) | *p*I |  | 24 h | 48 h |  |  |  |
| **L51** | Ketol-acid reductoisomerase | [gi|326523455](http://www.matrixscience.com/cgi/protein_view.pl?file=../data/20091222/FtmmfaSmh.dat&hit=1) | Hordeum vulgare subsp. Vulgare | 362 | 100.00 | 56.08/62.51 | 5.88/6.05 |  | -1.87 | 1.58 | mit | 14 | 31 |
| **Detoxification and defense** | | | | | | | | | | | | | |
|  | | | | | | | | | | | | | |
| **L64** | PREDICTED: glyoxylate reductase-like isoform 1 | gi|357128497 | Brachypodium distachyon | 263 | 100.00 | 39.87/34.60 | 5.80/5.76 |  | -1.71 | 1.74 | mit | 7 | 30 |
| **Proteins metabolism** | | | | | | | | | | | | | |
| **L44** | 26S protease regulatory subunit-like protein | [gi|283777738](http://www.matrixscience.com/cgi/protein_view.pl?file=../data/20091222/FtmmfaYme.dat&hit=1) | Lolium perenne | 125 | 100.00 | 51.84/48.22 | 4.90/4.84 |  | - | 2.44 | cyt | 7 | 21 |
| **Unknown proteins** | | | | | | | | | | | | | |
| **L3** | hypothetical protein | gi|307110690 | chlrella variabilis | 75 | 96.82 | 68.33/99.10 | 5.69/5.33 |  | + | + | cyt | 16 | 21 |
| **L11** | hypothetical protein | gi|125580806 | Oryza sativa Japonica | 74 | 96.17 | 44.00/46.73 | 6.17/8.11 |  | 1.97 | 1.53 | nuc | 12 | 21 |
| **L33** | unknown | [gi|238014852](http://www.matrixscience.com/cgi/protein_view.pl?file=../data/20091222/FtmmfaHTO.dat&hit=1) | Zea mays | 74 | 96.34 | 22.76/13.87 | 5.00/4.93 |  | 1.87 | 4.04 | mit | 8 | 43 |
| **L88** | Pentapeptide repeats | gi|326523645 | Hordeum vulgare subsp. Vulgare | 99 | 100.00 | 13.77/21.02 | 5.10/6.06 |  | + | + | chl | 6 | 43 |

^a^ Ps indicates protein score.

^b^ C.I. % indicates the Confidence interval.

^c^ “Exp” indicates experimental, “The” refers to Theoretical.

^d^ L, subcellular location; chl, chloroplast; cyt, cytoplasm; mit, mitochondrial; nuc,: nuclear; ER, Edoplasmic reticulum; GA, Golgi apparatus.;

^e^ M refers to number of peptides matched.

^f^ C indicates coverage rate;

**^#^** 24 h and 48 h represent the drought-treatment time. The fold change were calculated as the intensity of a DEP at 24 h or 48 h of drought-treatmet diveded by that at 0 h (non-treatment).

“-” represents protein spots detected only in the leaves of the control plants (0 h of drought-treatment)but absent in the drought-treated plants (24 h and 48 h of drought-treatment), “+” represents protein spots detected only in the leaves of drought-treated plants but not in the control ones.
