# Supplementary material for: Physiological and Comparative Proteomic Analysis Reveals Different Drought Responses in Roots and Leaves of Drought-Tolerant Wild Wheat (Triticum boeoticum)
Source: PLoS One. 2015 Apr 10;10(4):e0121852. doi: 10.1371/journal.pone.0121852 (PMC4393031; doi:10.1371/journal.pone.0121852)
Supplement: S5 Table — (DOC) [file pone.0121852.s008.doc]

**S5 Table.** Differentially changed proteins in the roots of wild wheat (*T. boeoticum*) plants under drought-treatment identified by MALDI-TOF-TOF

| **Spot ID** | **Protein annotation** | **gi in NCBI** | **Species** | **Ps^a^** | **Ps C.I. %^b^** | **Exp/The^c^** | |  | **Fold-change^#^** | | **L^d^** | **M^e^** | **C^f^ (%)** |
| --- | --- | --- | --- | --- | --- | --- | --- | --- | --- | --- | --- | --- | --- |
|  |  |  |  |  |  | **Mr (kDa)** | **p*I*** |  | **24 h** | **48 h** |  |  |  |
| **Up-regulated** | | | | | | | | | | | | | |
| **Carbon metabolism** | | | | | | | | | | | | | |
| R7 | UDP-glucose/GDP-mannose dehydrogenase | gi|326509649 | Hordeum vulgare subsp. vulgare | 227 | 100.00 | 54.35/44.92 | 5.69/5.70 |  | 2.14 | 1.56 | cyt | 6 | 17 |
| R22 | Transketolase | gi|326533372 | Hordeum vulgare subsp. vulgare | 924 | 100.00 | 71.62/74.03 | 5.61/5.45 |  | 1.76 | 1.58 | cyt | 23 | 45 |
| R37 | Transaldolase-like protein | gi|326513012 | Hordeum vulgare subsp. vulgare | 929 | 100.00 | 46.94/46.50 | 5.27/5.96 |  | 1.83 | 1.52 | cyt | 17 | 46 |
| R48 | fructose-bisphosphate aldolase | gi|226316439 | Secale cereale | 94 | 99.96 | 36.28/39.23 | 6.58/6.39 |  | + | + | cyt | 5 | 18 |
| R52 | PREDICTED: ribulose-phosphate 3-epimerase | gi|357113872 | Brachypodium distachyon | 116 | 100.00 | 24.93/29.44 | 5.91/7.70 |  | + | + | mit | 3 | 17 |
| R74 | PREDICTED: 6-phosphofructokinase 2-like | gi|357164069 | Brachypodium distachyon | 95 | 99.97 | 47.50/51.05 | 6.54/5.87 |  | 4.28 | 5.75 | mit | 7 | 15 |
| R84 | fructose-bisphosphate aldolase | gi|226316439 | Secale cereale | 88 | 99.82 | 36.03/39.23 | 6.45/6.39 |  | 2.00 | 2.34 | cyt | 4 | 18 |
| **Amino acid metabolism** | | | | | | | | | | | | | |
| R47 | methionine synthase 1 enzyme | gi|68655495 | Hordeum vulgare subsp. Vulgare | 197 | 100.00 | 61.59/84.85 | 6.13/5.74 |  | + | + | cyt | 7 | 15 |
| R61 | Aspartate aminotransferase | gi|75104955 | Pinus pinaster | 105 | 100.00 | 40.68/53.45 | 6.57/7.49 |  | + | + | mit | 5 | 12 |
| **Spot ID** | **Protein annotation** | **gi in NCBI** | **Species** | **Ps^a^** | **Ps C.I. %^b^** | **Exp/The^c^** | |  | **Fold-change^#^** | | **L^d^** | **M^e^** | **C^f^ (%)** |
|  |  |  |  |  |  | **Mr (kDa)** | **p*I*** |  | **24 h** | **48 h** |  |  |  |
| R65 | glutamate decarboxylase, putative, expressed | gi|300681536 | Triticum aestivum | 152 | 100.00 | 50.83/54.47 | 5.74/5.55 |  | 4.99 | 3.72 | cyt | 10 | 28 |
| R69 | methionine synthase 1 enzyme | gi|68655495 | Hordeum vulgare subsp. Vulgare | 538 | 100.00 | 61.24/84.85 | 6.25/5.74 |  | 3.04 | 1.54 | cyt | 16 | 26 |
| **Detoxification and defense** | | | | | | | | | | | | | |
| R49 | Horseradish peroxidase | gi|326521510 | Hordeum vulgare subsp. vulgare | 172 | 100.00 | 31.09/37.34 | 6.55/5.11 |  | + | + | mit | 4 | 17 |
| R53 | PREDICTED: germin-like protein 1-1-like | gi|357131697 | Brachypodium distachyon | 243 | 100.00 | 24.10/22.57 | 6.16/8.62 |  | + | + | mit | 3 | 26 |
| R54 | glutathione S-transferase-N | gi|326490674 | Hordeum vulgare subsp. vulgare | 76 | 97.10 | 25.36/25.41 | 6.19/5.22 |  | + | + | mit | 5 | 25 |
| R55 | pathogenesis-related protein | gi|162414848 | Triticum aestivum | 81 | 99.24 | 16.94/17.14 | 5.04/5.19 |  | + | + | nuc | 5 | 45 |
| R67 | thioredoxin-disulfide reductase | gi|326493572 | Hordeum vulgare subsp. Vulgare | 180 | 100.00 | 33.49/35.03 | 6.12/5.83 |  | 2.76 | 1.97 | cyt | 6 | 34 |
| R71 | thaumatin-like protein TLP5 | gi|326508997 | Hordeum vulgare subsp. vulgare | 233 | 100.00 | 22.67/25.79 | 6.46/6.04 |  | 3.27 | 7.73 | vac | 4 | 28 |
| R77 | ascorbate peroxidase | gi|15808777 | Hordeum vulgare subsp. vulgare | 102 | 99.99 | 25.82/16.91 | 5.87/5.97 |  | 5.19 | 4.24 | mit | 5 | 33 |
| R83 | glutathione S-transferase | gi|326529393 | Hordeum vulgare subsp. vulgare | 108 | 100.00 | 25.64/25.54 | 6.51/6.46 |  | 4.85 | 2.68 | cyt | 3 | 21 |
| R85 | class III peroxidase | gi|204309003 | Triticum aestivum | 235 | 100.00 | 40.06/6.61 | 6.61/6.45 |  | 1.67 | 1.73 | mit | 9 | 37 |
| **Chaperone** | | | | | | | | | | | | | |
| R76 | HSP70 | gi|326494024 | Hordeum vulgare subsp. vulgare | 482 | 100.00 | 43.93/42.02 | 5.45/5.30 |  | 5.62 | 2.09 | cyt | 11 | 44 |
| **Energy metabolism** | | | | | | | | | | | | | |
| **Spot ID** | **Protein annotation** | **gi in NCBI** | **Species** | **Ps^a^** | **Ps C.I. %^b^** | **Exp/The^c^** | |  | **Fold-change^#^** | | **L^d^** | **M^e^** | **C^f^ (%)** |
|  |  |  |  |  |  | **Mr (kDa)** | **p*I*** |  | **24 h** | **48 h** |  |  |  |
| R72 | Respiratory-chain NADH dehydrogenase 24 Kd subunit | gi|326502384 | Hordeum vulgare subsp. vulgare | 334 | 100.00 | 27.07/30.55 | 6.40/7.56 |  | 2.39 | 2.52 | mit | 12 | 46 |
| R79 | PREDICTED: ATP synthase subunit d,mitchondrial-like | gi|357122954 | Brachypodium distachyon | 100 | 99.99 | 17.47/19.61 | 5.97/5.32 |  | 42.97 | 40.18 | mit | 5 | 32 |
| R81 | PREDICTED: adenosine kinase 2-like | gi|357150251 | Brachypodium distachyon | 74 | 96.34 | 40.91/37.81 | 5.01/5.01 |  | 1.83 | 1.62 | cyt | 4 | 20 |
| **Signal transduction-associated proteins** | | | | | | | | | | | | | |
| R11 | PREDICTED: probable calcium-binding protein CML49-like isoform 1 | gi|357161047 | Brachypodium distachyon | 285 | 100.00 | 35.60/33.92 | 5.44/5.75 |  | 2.63 | 2.04 | nuc | 5 | 18 |
| R44 | Pyrabactin resistance 1 (PYR1) | gi|326506676 | Hordeum vulgare subsp. vulgare | 215 | 100.00 | 29.59/22.86 | 5.00/4.99 |  | 2.7 | 5.2 | EM | 6 | 34 |
| R50 | PREDICTED: 14-3-3-like protein GF14-B-like | gi|357163980 | Brachypodium distachyon | 151 | 100.00 | 32.19/29.77 | 4.70/4.71 |  | 4.75 | 6.51 | nuc | 9 | 44 |
| R80 | PREDICTED: probable calcium-binding protein CML28-like | gi|357154924 | Brachypodium distachyon | 75 | 96.96 | 23.08/19.28 | 6.13/4.45 |  | 13.06 | 7.01 | cyt | 10 | 36 |
| **Protein metabolism** | | | | | | | | | | | | | |
| R66 | PREDICTED: proteasome subunit beta type-7-A-like | gi|357125970 | Brachypodium distachyon | 189 | 100.00 | 25.50/29.40 | 6.04/6.60 |  | 2.29 | 1.76 | mit | 3 | 15 |
| **Spot ID** | **Protein annotation** | **gi in NCBI** | **Species** | **Ps^a^** | **Ps C.I. %^b^** | **Exp/The^c^** | |  | **Fold-change^#^** | | **L^d^** | **M^e^** | **C^f^ (%)** |
|  |  |  |  |  |  | **Mr (kDa)** | **p*I*** |  | **24 h** | **48 h** |  |  |  |
| **Lipid metabolism** | | | | | | | | | | | | | |
| R57 | temperature-induced lipocalin-2 | gi|77744851 | Triticum aestivum | 371 | 100.00 | 18.51/20.94 | 6.20/5.96 |  | + | + | cyt | 10 | 47 |
| **Transcription and translation-associated proteins** | | | | | | | | | | | | | |
| R78 | Alba protein | gi|326492075 | Hordeum vulgare subsp. vulgare | 151 | 100.00 | 17.40/14.62 | 5.34/5.04 |  | 10.22 | 4.03 | nuc | 3 | 27 |
| **Nucleotide metabolism** | | | | | | | | | | | | | |
| R60 | PREDICTED: putative nuclease HARBI1-like | gi|357111421 | Brachypodium distachyon | 73 | 95.07 | 64.72/46.36 | 6.51/6.57 |  | + | + | mit | 12 | 34 |
| R82 | inosine monophosphate dehydrogenase | gi|28413147 | Vigna unguiculata | 123 | 100.00 | 49.76/53.53 | 6.15/6.34 |  | -1.45 | 2.20 | mit | 5 | 18 |
| **cyto and cytkinesis-associated proteins** | | | | | | | | | | | | | |
| R59 | Actin-53 | gi|3219763 | Nicotiana tabacum | 79 | 98.70 | 64.93/37.46 | 6.59/5.39 |  | + | + | cyto | 3 | 17 |
| **Down-regulated** | | | | | | | | | | | | | |
| **Carbon metabolism** | | | | | | | | | | | | | |
| R1 | Triose-phosphate isomerase | gi|1174745 | Secale cereale | 85 | 99.68 | 25.85/31.96 | 5.20/6.00 |  | -1.92 | -2.68 | EM | 4 | 23 |
| R5 | Enolase, putative, expressed | gi|110288669 | Oryza sativa Japonica Group | 137 | 100.00 | 54.00/51.83 | 5.40/5.84 |  | -3.12 | -4.09 | cyt | 3 | 11 |
| R6 | Glycosyltransferases | gi|326525745 | Hordeum vulgare subsp. vulgare | 112 | 100.00 | 51.12/51.47 | 5.56/5.26 |  | 1.01 | -2.56 | mit | 4 | 14 |
| R15 | PEP phosphonomutase | gi|326499686 | Hordeum vulgare subsp. vulgare | 137 | 100.00 | 33.31/34.17 | 5.32/5.41 |  | -2.33 | -5.80 | mit | 4 | 20 |
| **Spot ID** | **Protein annotation** | **gi in NCBI** | **Species** | **Ps^a^** | **Ps C.I. %^b^** | **Exp/The^c^** | |  | **Fold-change^#^** | | **L^d^** | **M^e^** | **C^f^ (%)** |
|  |  |  |  |  |  | **Mr (kDa)** | **p*I*** |  | **24 h** | **48 h** |  |  |  |
| R23 | Phosphoglycerate kinase (PGK) | gi|326522650 | Hordeum vulgare subsp. vulgare | 333 | 100.00 | 41.59/40.22 | 5.72/5.39 |  | -3.76 | -1.56 | cyt | 12 | 38 |
| R26 | phosphoglucomutase | gi|18076790 | Triticum aestivum | 277 | 100.00 | 61.69/62.98 | 5.74/5.66 |  | -2.70 | -3.32 | cyt | 13 | 28 |
| R29 | D-3-phosphoglycerate dehydrogenase | gi|326505978 | Hordeum vulgare subsp. vulgare | 142 | 100.00 | 59.24/64.30 | 5.88/6.41 |  | -4.65 | -2.19 | mit | 7 | 17 |
| R30 | Pyruvate decarboxylase isozyme 2 | gi|158513193 | Oryza sativa Indica Group | 320 | 100.00 | 58.20/65.89 | 5.75/5.60 |  | -3.18 | -2.18 | cyt | 10 | 21 |
| R33 | pyrophosphate-dependent phosphofructokinase | gi|115467370 | Oryza sativa Japonica Group | 180 | 100.00 | 61.80/61.91 | 5.22/6.01 |  | -1.20 | -1.85 | cyt | 10 | 22 |
| R35 | formate dehydrogenase | gi|326507324 | Hordeum vulgare subsp. vulgare | 431 | 100.00 | 42.14/41.69 | 6.82/6.51 |  | -5.06 | -2.52 | mit | 11 | 29 |
| R36 | D-3-phosphoglycerate dehydrogenase | gi|326505978 | Hordeum vulgare subsp. vulgare | 350 | 100.00 | 52.06/64.30 | 5.20/6.41 |  | -5.19 | -2.29 | mit | 15 | 29 |
| R40 | Pyruvate decarboxylase isozyme 2 | gi|158513193 | Oryza sativa Indica Group | 300 | 100.00 | 57.26/65.89 | 5.86/5.60 |  | -1.75 | -1.66 | cyt | 8 | 18 |
| R41 | ribulose-1,5-bisphosphate carboxylase/oxygenase large subunit | gi|144583566 | Crithopsis delileana | 836 | 100.00 | 48.79/52.44 | 6.35/6.04 |  | -2.38 | -2.99 | cyt | 27 | 53 |
| R43 | PREDICTED: glyceraldehyde-3-phosphate dehydrogenase | gi|357117328 | Brachypodium distachyon | 201 | 100.00 | 35.73/43.00 | 6.82/8.44 |  | -1.65 | -2.21 | cyt | 10 | 40 |
| R46 | phosphoglycerate mutase | gi|32400802 | Triticum aestivum | 301 | 100.00 | 59.83/29.62 | 5.60/5.43 |  | -2.84 | -1.83 | cyt | 9 | 56 |
| **Amino acid metabolism** | | | | | | | | | | | | | |
| **Spot ID** | **Protein annotation** | **gi in NCBI** | **Species** | **Ps^a^** | **Ps C.I. %^b^** | **Exp/The^c^** | |  | **Fold-change^#^** | | **L^d^** | **M^e^** | **C^f^ (%)** |
|  |  |  |  |  |  | **Mr (kDa)** | **p*I*** |  | **24 h** | **48 h** |  |  |  |
| R8 | S-adenosyl-L-homocysteine hydrolase | gi|68655456 | Hordeum vulgare subsp. vulgare | 482 | 100.00 | 49.88/49.96 | 5.67/5.81 |  | -1.38 | -2.56 | mit | 16 | 40 |
| R17 | plastid glutamine synthetase 2 | gi|251832986 | Triticum aestivum | 349 | 100.00 | 43.57/47.00 | 5.17/5.75 |  | -3.02 | -1.78 | mit | 9 | 31 |
| R21 | 5-enolpyruvylshikimate 3-phosphate synthase | gi|13375567 | Lolium rigidum | 362 | 100.00 | 48.10/37.10 | 5.40/5.59 |  | -3.60 | -5.16 | mit | 8 | 25 |
| R28 | 5,10-methylene-tetrahydrofolate reductase | gi|115589742 | Triticum monococcum | 308 | 100.00 | 57.28/65.46 | 5.81/5.86 |  | -3.94 | -3.25 | cyt | 16 | 42 |
| R32 | Aspartate aminotransferase | gi|326513056 | Hordeum vulgare subsp. vulgare | 191 | 100.00 | 44.37/45.36 | 6.15/5.75 |  | -3.01 | -2.30 | mit | 9 | 32 |
| R34 | Phenylalanine ammonia-lyase | gi|326526355 | Hordeum vulgare subsp. vulgare | 650 | 100.00 | 65.50/76.20 | 6.13/5.89 |  | -2.34 | -6.78 | mit | 16 | 30 |
| R63 | Serine proteases of the peptidase family S9A | gi|326517006 | Hordeum vulgare subsp. vulgare | 105 | 100.00 | 81.85/87.58 | 5.69/5.87 |  | - | - | cyt | 10 | 16 |
| **Detoxification and defense** | | | | | | | | | | | | | |
| R16 | Glyoxalase I | gi|326493416 | Hordeum vulgare subsp. vulgare | 110 | 100.00 | 32.43/32.81 | 5.42/5.34 |  | -2.53 | -2.26 | cyt | 5 | 19 |
| R64 | cytosolic glutathione peroxidase | gi|34334012 | Triticum monococcum | 339 | 100.00 | 17.71/18.61 | 6.85/6.73 |  | -8.64 | -9.01 | cyt | 13 | 82 |
| **Chaperone** | | | | | | | | | | | | | |
| R13 | 70 kDa peptidyl-prolyl isomerase | gi|3023751 | Triticum aestivum | 377 | 100.00 | 63.74/62.25 | 5.63/5.30 |  | -3.12 | -17.07 | cyt | 14 | 26 |
| R18 | heat shock cognate protein HSC70 | gi|2655420 | Brassica napus | 629 | 100.00 | 65.81/71.13 | 5.27/5.08 |  | -4.82 | - | cyt | 17 | 36 |
| R19 | 70-kDa heat shock protein 5 | gi|326495158 | Hordeum vulgare subsp. Vulgare | 818 | 100.00 | 72.58/73.30 | 5.29/5.11 |  | -4.39 | - | ER | 23 | 36 |
| **Spot ID** | **Protein annotation** | **gi in NCBI** | **Species** | **Ps^a^** | **Ps C.I. %^b^** | **Exp/The^c^** | |  | **Fold-change^#^** | | **L^d^** | **M^e^** | **C^f^ (%)** |
|  |  |  |  |  |  | **Mr (kDa)** | **p*I*** |  | **24 h** | **48 h** |  |  |  |
| R20 | 70-kDa heat shock protein 5 | gi|326495158 | Hordeum vulgare subsp. vulgare | 354 | 100.00 | 71.70/73.30 | 5.34/5.11 |  | -3.85 | - | ER | 14 | 25 |
| R25 | TCP-1 (CTT or eukaryotic type II) chaperonin | gi|326501026 | Hordeum vulgare subsp. vulgare | 435 | 100.00 | 54.52/60.96 | 5.73/5.45 |  | -3.53 | -1.78 | cyt | 12 | 24 |
| R38 | hsp70 (AA 6 - 651) | gi|20559 | Petunia x hybrida | 219 | 100.00 | 65.20/71.14 | 5.22/5.07 |  | -5.34 | -35.37 | cyt | 13 | 30 |
| **Energy metabolism** | | | | | | | | | | | | | |
| R3 | ATP synthase beta subunit | gi|110915684 | Poa billardierei | 514 | 100.00 | 53.31/53.03 | 5.27/5.17 |  | - | -2.69 | cyt | 15 | 50 |
| R4 | ATP synthase CF1 beta subunit | gi|108773085 | Scenedesmus obliquus | 391 | 100.00 | 49.73/51.91 | 5.53/5.25 |  | -1.74 | -2.88 | cyt | 9 | 28 |
| R27 | ATP synthase beta subunit | gi|525291 | Triticum aestivum | 696 | 100.00 | 59.92/59.33 | 5.78/5.56 |  | -4.08 | -4.65 | mit | 15 | 37 |
| **Signal transduction-associated proteins** | | | | | | | | | | | | | |
| R24 | rab-GDP dissociation inhibitor | gi|326529099 | Hordeum vulgare subsp. vulgare | 227 | 100.00 | 48.55/50.17 | 5.85/5.51 |  | -1.51 | -2.92 | cyt | 13 | 35 |
| **Protein metabolism** | | | | | | | | | | | | | |
| R12 | Predicted Zn-dependent peptidases | gi|326513540 | Hordeum vulgare subsp. vulgare | 221 | 100.00 | 56.17/58.53 | 5.78/6.05 |  | -3.88 | -9.71 | mit | 12 | 19 |
| R14 | 26S protease regulatory subunit 7 | gi|307109661 | Chlorella variabilis | 212 | 100.00 | 47.04/47.08 | 6.27/8.09 |  | 1.18 | -10.07 | cyt | 9 | 19 |
| **Lipid metabolism** | | | | | | | | | | | | | |
| R2 | PREDICTED: protein usf-like | gi|357135121 | Brachypodium distachyon | 149 | 100.00 | 27.49/26.49 | 5.30/5.03 |  | -1.07 | -3.68 | cyt | 3 | 16 |
| R9 | putative phospholipase D alpha 1 precursor | gi|209944121 | Triticum monococcum | 511 | 100.00 | 86.54/62.28 | 5.67/5.40 |  | - | -4.81 | cyt | 18 | 44 |
| **Spot ID** | **Protein annotation** | **gi in NCBI** | **Species** | **Ps^a^** | **Ps C.I. %^b^** | **Exp/The^c^** | |  | **Fold-change^#^** | | **L^d^** | **M^e^** | **C^f^ (%)** |
|  |  |  |  |  |  | **Mr (kDa)** | **p*I*** |  | **24 h** | **48 h** |  |  |  |
| **Transcription and translation-associated proteins** | | | | | | | | | | | | | |
| R10 | asparaginyl-tRNA synthetase | gi|326498091 | Hordeum vulgare subsp. vulgare | 74 | 95.71 | 58.36/63.67 | 6.06/5.65 |  | -2.37 | -3.54 | cyt | 9 | 19 |
| R31 | eukaryotic translation initiation factor 3 subunit A | gi|162459484 | Zea mays | 76 | 97.29 | 55.09/111.89 | 6.11/9.29 |  | -2.71 | -1.74 | cyt | 17 | 18 |
| R42 | PREDICTED: eukaryotic translation initiation factor 3 subunit H-like | gi|357162977 | Brachypodium distachyon | 254 | 100.00 | 40.26/39.24 | 5.02/4.87 |  | -20.02 | -13.63 | cyt | 8 | 23 |
| R62 | Alanyl-tRNA synthetase | gi|326519084 | Hordeum vulgare subsp. vulgare | 156 | 100.00 | 120.73/110.55 | 6.09/5.90 |  | - | - | mit | 11 | 15 |
| **Up-regulated at 24h but down-regulated at 48h** | | | | | | | | | | | | | |
| **Energy metabolism** | | | | | | | | | | | | | |
| R75 | Adenylate kinase (ADK) | gi|326517593 | Hordeum vulgare subsp. vulgare | 285 | 100.00 | 29.25/26.64 | 6.76/6.54 |  | 3.28 | - | cyt | 9 | 48 |
| **Unknown proteins** | | | | | | | | | | | | | |
| R39 | hypothetical protein | gi|145349320 | Ostreococcus lucimarinus CCE9901 | 77 | 97.80 | 48.26/29.63 | 5.67/9.11 |  | -6.63 | -8.11 | mit | 10 | 34 |
| R45 | hypothetical protein | gi|242049774 | Sorghum bicolor | 92 | 99.94 | 25.90/24.21 | 5.53/5.54 |  | 1.23 | -1.85 | mit | 2 | 14 |
| R51 | hypothetical protein | gi|302762607 | Selaginella moellendorffii | 73 | 95.07 | 60.40/38.22 | 6.18/5.14 |  | + | + | cyt | 9 | 48 |
| R56 | hypothetical protein | gi|242066452 | Sorghum bicolor | 77 | 98.04 | 17.83/27.69 | 5.78/9.43 |  | + | + | mit | 5 | 21 |
| R58 | hypothetical protein | gi|125547139 | Oryza sativa Indica Group | 80 | 98.97 | 17.26/50.51 | 6.35/5.04 |  | + | + | nuc | 13 | 34 |
| **Spot ID** | **Protein annotation** | **gi in NCBI** | **Species** | **Ps^a^** | **Ps C.I. %^b^** | **Exp/The^c^** | |  | **Fold-change^#^** | | **L^d^** | **M^e^** | **C^f^ (%)** |
|  |  |  |  |  |  | **Mr (kDa)** | **p*I*** |  | **24 h** | **48 h** |  |  |  |
| R68 | hypothetical protein | gi|242091237 | Sorghum bicolor | 85 | 99.68 | 54.65/61.65 | 5.96/6.34 |  | 2.27 | - | mit | 11 | 22 |
| R70 | hypothetical protein | gi|255081574 | Micromonas sp. RCC299 | 78 | 98.17 | 22.98/108.58 | 6.43/8.74 |  | 3.87 | 4.25 | mit | 20 | 21 |
| R73 | Uncharacterized protein | gi|51969886 | Arabidopsis thaliana | 79 | 98.58 | 30.28/33.71 | 6.48/6.05 |  | 4.58 | - | EM | 3 | 21 |

^a^ Ps indicates protein score.

^b^ C.I. % indicates the Confidence interval.

^c^ “Exp” indicates experimental, “The” refers to Theoretical.

^d^ L,subcellular location; chl, chloroplast; cyt, cytoplasm; cyto, cytoskeleton; mit, mitochondria; nuc,: nuclear; ER, Edoplasmic reticulum; vac, vacuole.; EM, Extracellular matrix

^e^ M refers to number of peptides matched.

^f^ C indicates coverage rate;

**^#^** 24 h and 48 h represent the drought treatment time. The fold change were calculated by the spot intensity of 24 h or 48 h diving the value at 0 h(non-treatment).

“-” represents protein spots detected only in the roots of control plants (0 h of drought-treatment) but absent in the roots of the drought-treated plants (24 h and 48 h of drought-treatment), “+” represents protein spots detected only in the roots of the drought-treated plants but not in the roots of the control ones.
