# Supplementary material for: Physiological and Comparative Proteomic Analysis Reveals Different Drought Responses in Roots and Leaves of Drought-Tolerant Wild Wheat (Triticum boeoticum)
Source: PLoS One. 2015 Apr 10;10(4):e0121852. doi: 10.1371/journal.pone.0121852 (PMC4393031; doi:10.1371/journal.pone.0121852)
Supplement: S6 Table — (DOC) [file pone.0121852.s009.doc]

**S6 Table.** The common differentially expressed proteins identified in the leaves and the roots of the wild wheat (*T. boeoticum*) plants exposure to 20% PEG6000 for 48 h of drought-treatment.

| **Organs^＊^** | **Accession** | **Spot ID** | **Protein annotation** | **Possible function** | **24 h** | **48 h** |
| --- | --- | --- | --- | --- | --- | --- |
| **R** | gi|115589742 | R28 | 5,10-methylene-tetrahydrofolate reductase | Amino acid metabolism | -3.94 | -3.25 |
| **L** | gi|115589742 | L50 | 5,10-methylene-tetrahydrofolate reductase | Amino acid metabolism | -1.88 | -2.48 |
| **R** | gi|144583566 | R41 | ribulose-1,5-bisphosphate carboxylase/oxygenase large subunit | photosynthesis | -2.38 | -2.99 |
| **L** | gi|144583566 | L4 | ribulose-1,5-bisphosphate carboxylase/oxygenase large subunit | photosynthesis | 2.94 | -1.31 |
| **L** | gi|144583566 | L5 | ribulose-1,5-bisphosphate carboxylase/oxygenase large subunit | photosynthesis | 6.91 | 1.93 |
| **L** | gi|144583566 | L10 | ribulose-1,5-bisphosphate carboxylase/oxygenase large subunit | photosynthesis | 3.18 | 1.02 |
| **L** | gi|144583566 | L52 | ribulose-1,5-bisphosphate carboxylase/oxygenase large subunit | photosynthesis | -1.89 | -2.31 |
| **L** | gi|144583566 | L69 | ribulose-1,5-bisphosphate carboxylase/oxygenase large subunit | photosynthesis | -1.82 | -1.83 |
| **R** | gi|300681536 | R65 | glutamate decarboxylase, putative, expressed | Amino acid metabolism | 4.99 | 3.72 |
| **L** | gi|300681536 | L42 | glutamate decarboxylase, putative, expressed | Amino acid metabolism | 1.68 | 1.10 |
| **R** | gi|326495158 | R19 | 70-kDa heat shock protein 5 | Chaperone | -4.39 | L |
| **R** | gi|326495158 | R20 | 70-kDa heat shock protein 5 | Chaperone | -3.85 | L |
| **L** | gi|326495158 | L48 | 70-kDa heat shock protein 5 | Chaperone | -1.88 | -2.48 |
| **R** | gi|326517593 | R75 | Adenylate kinase (ADK) | Energy metabolism | 3.28 | L |
| **Organs^＊^** | **Accession** | **Spot ID** | **Protein annotation** | **Possible function** | **24 h** | **48 h** |
| **L** | gi|326517593 | L85 | Adenylate kinase (ADK) | Energy metabolism | 2.16 | L |
| **R** | gi|326533372 | R22 | transketolase | Carbon metabolism | 1.76 | 1.58 |
| **L** | gi|326533372 | L46 | transketolase | Carbon metabolism | -2.02 | -2.44 |
| **L** | gi|326533372 | L47 | transketolase | Carbon metabolism | -1.53 | -1.67 |

**^＊^**R represents root; L represents leaf.
