# Supplementary material for: Physiological and Comparative Proteomic Analysis Reveals Different Drought Responses in Roots and Leaves of Drought-Tolerant Wild Wheat (Triticum boeoticum)
Source: PLoS One. 2015 Apr 10;10(4):e0121852. doi: 10.1371/journal.pone.0121852 (PMC4393031; doi:10.1371/journal.pone.0121852)
Supplement: S7 Table — (DOC) [file pone.0121852.s010.doc]

**S7 Table.** log_1.5_-transformed values of -fold change ratios of differential expressed proteins associated with amino acid and protein metabolism.

| **Leaves** | | | |  | **Roots** | | | |
| --- | --- | --- | --- | --- | --- | --- | --- | --- |
| **Spot ID** | **0 h** | **24h** | **48h** |  | **Spot ID** | **0 h** | **24h** | **48h** |
| **Amino acid metabolism** | | | | | | | | |
| L12 | 0.00 | 1.81 | 1.95 |  | R8 | 0.00 | -0.40 | -2.32 |
| L13 | 0.00 | 1.20 | 1.07 |  | R17 | 0.00 | -2.73 | -1.42 |
| L20 | 0.00 | 1.92 | 0.52 |  | R21 | 0.00 | -3.16 | -4.05 |
| L21 | 0.00 | 1.55 | -0.08 |  | R28 | 0.00 | -3.38 | -2.91 |
| L42 | 0.00 | 1.29 | 0.25 |  | R32 | 0.00 | -2.72 | -2.05 |
| L45 | 0.00 | -2.26 | -1.94 |  | R34 | 0.00 | -2.09 | -4.72 |
| L50 | 0.00 | -1.65 | -2.08 |  | R47 | 0.00 | 36.03 | 34.43 |
| L51 | 0.00 | -1.54 | 1.13 |  | R61 | 0.00 | 33.40 | 36.55 |
| L58 | 0.00 | -1.24 | 0.50 |  | R63 | 0.00 | -36.14 | -36.14 |
| L60 | 0.00 | -4.71 | -1.36 |  | R65 | 0.00 | 3.97 | 3.24 |
| L62 | 0.00 | 0.11 | 1.04 |  | R69 | 0.00 | 2.74 | 1.07 |
| L82 | 0.00 | 38.93 | 32.92 |  |  |  |  |  |
| L95 | 0.00 | 33.96 | 38.20 |  |  |  |  |  |
| **Protein synthesis** | | | | | | | | |
| L7 | 0.00 | 1.97 | 1.54 |  | R10 | 0.00 | -2.13 | -3.12 |
| L15 | 0.00 | 1.29 | 0.24 |  | R31 | 0.00 | -2.46 | -1.37 |
| L22 | 0.00 | 1.47 | 0.39 |  | R42 | 0.00 | -7.39 | -6.44 |
| L24 | 0.00 | 1.77 | 1.06 |  | R62 | 0.00 | -33.97 | -33.97 |
| L37 | 0.00 | 0.31 | 1.34 |  | R78 | 0.00 | 5.73 | 3.44 |
|  |  |  |  |  |  |  |  |  |
| L57 | 0.00 | -1.25 | -0.32 |  |  |  |  |  |
| L72 | 0.00 | -1.97 | 0.45 |  |  |  |  |  |
| L75 | 0 | 34.27 | 41.12 |  |  |  |  |  |
| L81 | 0.00 | 1.54 | 1.69 |  |  |  |  |  |
| **Chaperones** | | | | | | | | |
| L2 | 0.00 | -1.22 | -0.42 |  | R13 | 0.00 | -2.81 | -7.00 |
| L8 | 0.00 | 1.57 | 1.16 |  | R18 | 0.00 | -3.88 | -40.37 |
| L36 | 0.00 | 1.46 | 0.20 |  | R19 | 0.00 | -3.65 | -39.00 |
| L40 | 0.00 | 3.09 | 3.74 |  | R20 | 0.00 | -3.32 | -38.33 |
| L48 | 0.00 | -1.56 | -2.24 |  | R25 | 0.00 | -3.11 | -1.42 |
| L77 | 0.00 | 36.60 | 35.85 |  | R38 | 0.00 | -4.13 | -8.79 |
| L86 | 0.00 | 38.53 | 37.35 |  | R76 | 0.00 | 4.26 | 1.82 |
| L90 | 0.00 | 1.79 | -0.13 |  |  |  |  |  |
| L92 | 0.00 | 2.29 | 3.77 |  |  |  |  |  |
| **Proteins degradation** | | | | | | | | |
| L1 | 0.00 | 1.93 | 3.01 |  | R12 | 0.00 | -3.34 | -5.61 |
| L6 | 0.00 | 5.06 | 2.12 |  | R14 | 0.00 | 0.41 | -5.70 |
| L44 | 0.00 | -37.12 | 2.20 |  | R66 | 0.00 | 2.05 | 1.39 |
| L54 | 0.00 | -35.46 | -35.46 |  |  |  |  |  |
| L65 | 0.00 | -1.23 | 0.12 |  |  |  |  |  |
| L71 | 0.00 | 1.34 | 1.88 |  |  |  |  |  |
| **Leaves** | | | |  | **Roots** | | | |
| **Spot ID** | **0 h** | **24h** | **48h** |  | **Spot ID** | **0 h** | **24h** | **48h** |
| L76 | 0.00 | 35.76 | 36.03 |  |  |  |  |  |
| L89 | 0.00 | 39.44 | 40.04 |  |  |  |  |  |
| L91 | 0.00 | 33.77 | 37.01 |  |  |  |  |  |
